# Knockdown of SUCLG2 inhibits glioblastoma proliferation and promotes apoptosis through LMNA acetylation and the mediation of H4K16la lactylation

**DOI:** 10.1038/s41420-025-02856-4

**Published:** 2025-11-17

**Authors:** Wenshan Li, Qingqing Zhang, Hang Yin, Qiao Li, Shangyu Liu, Juncheng Wang, Guoqiang Yuan, Yawen Pan

**Affiliations:** 1 The Second Hospital of Lanzhou University, Lanzhou, Gansu China; 2 Department of Neurosurgery, Qinghai Provincial People’s Hospital, Xining, Qinghai China; 3 Department of Respiratory and Critical Care Medicine, Qinghai University Affiliated Hospital, Xining, Qinghai China; 4 Key Laboratory of Neurology of Gansu Province, The Second Hospital of Lanzhou University, Lanzhou, Gansu China

**Keywords:** Post-translational modifications, CNS cancer

## Abstract

Glioblastoma (GBM) is the most aggressive primary tumour in the central nervous system, and dynamic clonal evolution and interactions within the microenvironment cause its significant spatiotemporal heterogeneity. These interactions primarily manifest as metabolic reprogramming, mitochondrial dynamic imbalance, and epigenetic remodelling. SUCLG2 has been implicated in the progression of GBM; however, the underlying mechanism is unclear. This study aimed to investigate the role of SUCLG2 in the proliferation and apoptosis of GBM cells. SUCLG2 was found to interact with LMNA, leading to acetylation modification of its amino acid residue K470 and affecting limited oxidative phosphorylation levels and mitochondrial damage. SUCLG2 interacted with DLAT, reducing the binding of lactate-regulated protein H4K16la to promoter regions and cis-regulatory elements. This suppressed the expression of *BEST1*, *GRAMD4*, and *MBD6*, affecting the proliferation and apoptosis of GBM cells. These findings reveal a new SUCLG2-mediated mechanism in lactate metabolism and mitochondrial apoptosis in GBM and offer novel therapeutic and preventive targets for GBM.

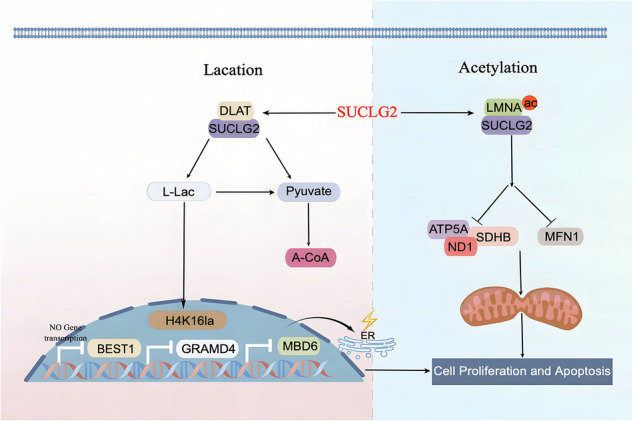

## Introduction

As the most aggressive primary malignant brain tumour in the central nervous system, glioblastoma (GBM) exhibits a significantly increasing incidence with age, particularly peaking at 75–84 years [1, 2]. Despite the recent advances in anti-tumour drugs, molecular targeted therapies, and immunotherapies, the survival rates among patients with GBM remain low, having 1- and 2-year survival rates of <40% and <18%, respectively [3, 4]. Notably, the heterogeneity of the tumour microenvironment (TME) and interactions between GBM cells substantially limit treatment efficacy [5]. Consequently, there is an urgent need for therapeutic strategies targeting specific molecular markers in the TME and tumour cells.

Succinate-coenzyme A (CoA) ligase GDP-forming subunit beta (SUCLG2) is crucial in the tricarboxylic acid (TCA) cycle, constituting the GTP-specific β-subunit of succinyl-CoA synthetase that catalyses the reversible conversion of succinyl-CoA to succinate. Notably, the enzyme is barely expressed in normal brain tissue [6, 7]. Metabolic tumour cell programming in the TME considerably influences tumour cell growth, maintenance, and proliferation [8]. During hypoxia, pyruvate is converted to lactate by lactate dehydrogenase (LDH), a reaction regenerating nicotinamide adenine dinucleotide, an essential compound for sustained glycolysis [9]. Even in normoxic conditions, cells may convert pyruvate to lactate. Warburg first described lactate production by tumour tissues and its release into the extracellular space [10]. The development of novel anti-tumour therapeutic strategies inhibiting glycolysis and induce mitochondrial damage has clinical significance for GBM prevention and treatment.

Post-translational histone modifications are crucial in the epigenetic regulatory mechanism [11]. Various histone acylation marks derived from cellular metabolites have been revealed, including acetylation, propionylation, butyrylation, 2-hydroxyisobutyrylation, succinylation, malonylation, glutarylation, crotonylation, and β-hydroxybutyrylation [12]. Additionally, glycolysis-derived lactate was recently identified as a histone lactylation substrate, and lactate-derived histone lactylation (e.g., at the H3K18la site) directly stimulates gene transcription [13, 14]. Moreover, histone lactylation regulates macrophage M1/2 polarisation, cellular metabolic reprogramming, and tumourigenesis [15, 16]. Furthermore, SUCLG2 deletion in T cells may cause heightened inflammatory responses, indicating its possible role in immune regulation [17]. These findings suggest that SUCLG2 is closely associated with proliferation and apoptosis in GBM, indicating its potential as a promising molecular target in GBM treatment.

Here, knockdown of SUCLG2 suppressed cell proliferation and regulated apoptosis by inhibiting mitochondrial oxidative phosphorylation and lactate metabolism. The interaction and acetylation of SUCLG2 and lamin A (LMNA) promoted MFN1 degradation, disrupting mitochondrial fusion and triggering restricted cell-cycle progression and cell death. Additionally, the interaction between SUCLG2 and dihydrolipoamide acetyltransferase (DLAT) promoted lactate reduction, affecting H4K16la-regulated transcription of *BEST1*, *GRAMD4*, and *MBD6*. This inhibited the secretion of cytokines interleukin (IL)-6 and IL-8, suppressing GBM cell proliferation, increasing endoplasmic reticulum stress, leading to cell injury, mediating mitochondrial apoptosis, and resulting in increased cell death. These findings provide a new theoretical basis for targeting SUCLG2 as a potential treatment for GBM.

## Results

### Increased SUCLG2 expression predicts glioma progression and poor prognosis

**Fig. 1:**
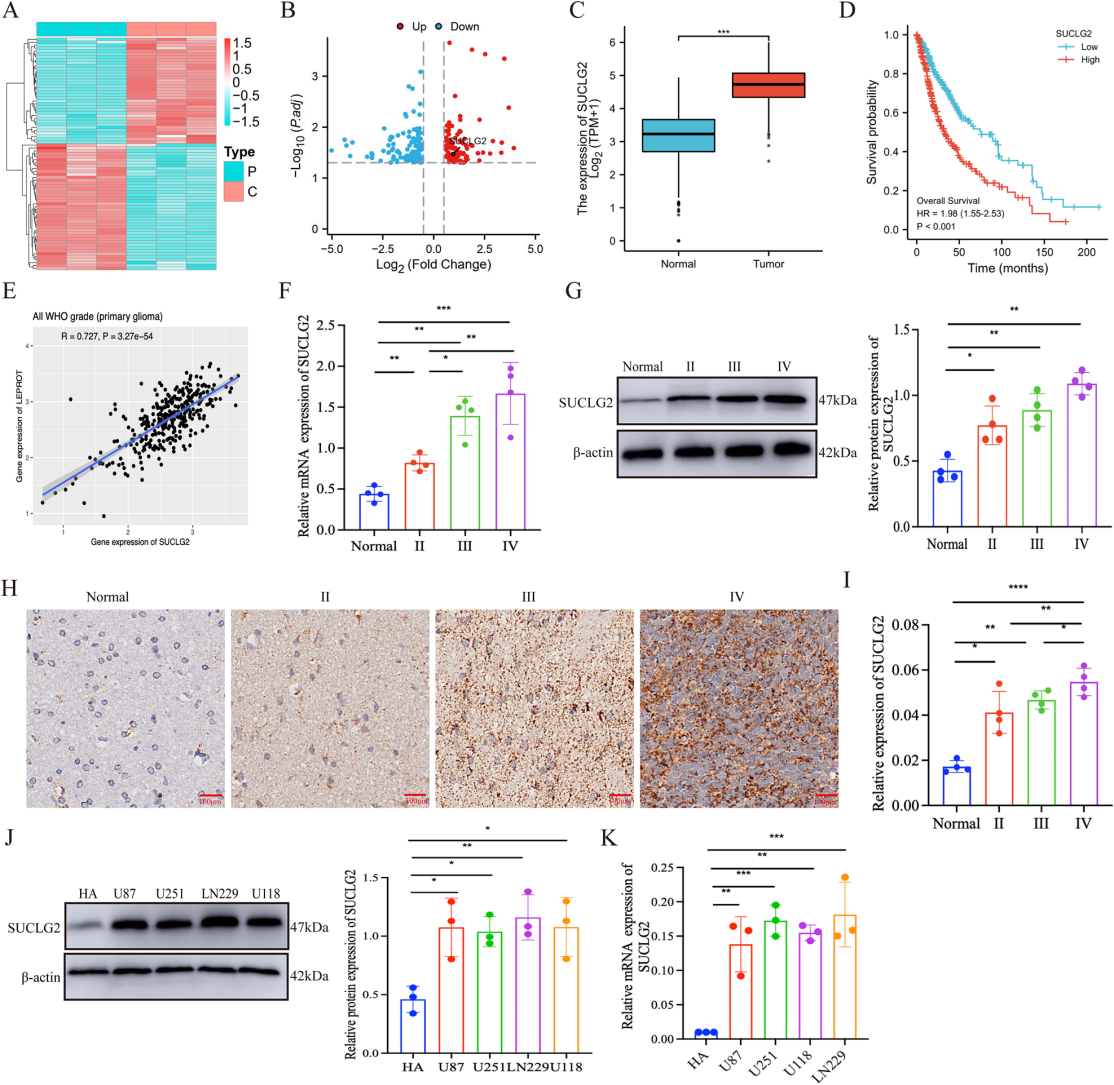
Increased SUCLG2 expression predicts glioma progression and poor prognosis. **A** Proteomics-based analysis of glioblastoma (GBM) core tissues with the differential protein expression in peritumour tissue, as shown in a heatmap analysis. **B** Volcano plot showing proteomic sequencing analysis of GBM core tissue and surrounding tumour tissue, showing high expression of SUCLG2. **C** Comparison of SUCLG2 expression in GBM and normal tissue samples using The Cancer Genome Atlas (TCGA) databases. **D** In patients grouped by high or low SUCLG2 expression, Kaplan–Meier curves were plotted for prognostic analyses. **E** Correlation analysis of SUCLG2 expression in all WHO grade tissues based on the TCGA dataset. **F**, **G** SUCLG2 expression levels revealed by real-time quantitative polymerase chain reaction (RT-qPCR) and western blotting in the tissues of different clinically-graded gliomas and normal brain injury tissue. Western blot analysis of SUCLG2 expression in glioma Grades 2 (*n* = 4), 3 (*n* = 4), and 4 (*n* = 4) and normal brain injury tissue (*n* = 4); β-Actin was used as a control. **H**, **I** Representative immunohistochemistry plots showing SUCLG2 expression in clinical glioma tissues and normal brain injury tissue. **J** Expression levels of SUCLG2 were assessed via western blotting in different glioma cell lines and HA. **K** Expression of SUCLG2 in different GBM cell lines relative to HA, as revealed by RT-qPCR; *P < 0.05, **P < 0.01, ***P < 0.001.

We performed paired proteomic analysis on tumour cores and surrounding tissues surgically resected from three patients diagnosed with high-grade glioma to investigate the differences in protein expression. We identified 102 significantly downregulated and 109 significantly upregulated proteins. Compared with the tumour periphery, SUCLG2 protein expression was significantly elevated in the core GBM region (fold change >2.5; false discovery rate ≤0.001) (Fig. 1A, B). We conducted an in-depth analysis using public databases to further validate the clinical significance of SUCLG2. The Cancer Genome Atlas database and Gene Expression Profile Interaction Analysis 2 results showed that SUCLG2 expression levels were significantly higher in GBM samples than in normal tissues (Figs. 1C, S1A). Additionally, the Kaplan–Meier survival and correlation analyses indicated that elevated SUCLG2 expression levels were significantly positively correlated with poor overall survival rates in patients with glioma (Fig. 1D, E). Furthermore, SUCLG2 expression was significantly elevated in various cancer tissues, including those of GBM, breast cancer, cholangiocarcinoma, bladder cancer, and lung cancer (Fig. S1B).

Using the injured brain as control, we conducted a multidimensional analysis on various grades of GBM tissues to clarify the clinical relevance of SUCLG2 in GBM progression. We examined the SUCLG2 expression levels in 12 glioma tissue samples (four each from Grades II, III, and IV) and four samples of injured brain tissues using western blot analysis, real-time quantitative polymerase chain reaction (RT-qPCR), and immunohistochemistry (IHC). SUCLG2 expression was significantly higher in GBM tissues than in injured brain tissues, with markedly elevated levels in the pseudofenestrated necrotic regions. Furthermore, SUCLG2 expression was significantly and positively correlated with the tumour pathological grade (Fig. 1F–I). We assessed SUCLG2 expression in the normal glial cell line HA and various GBM cell lines (U87, U251, LN229, and U118) to further validate these findings. Western blot analysis and RT-qPCR demonstrated that SUCLG2 expression was significantly higher in GBM cell lines than in HA (Fig. 1J, K). These results, in conjunction with the bioinformatic analysis findings, underscore that elevated SUCLG2 expression is closely associated with malignant progression and poor prognosis in GBM.

### Knockdown of SUCLG2 modulates proliferation and apoptosis in GBM cells

**Fig. 2:**
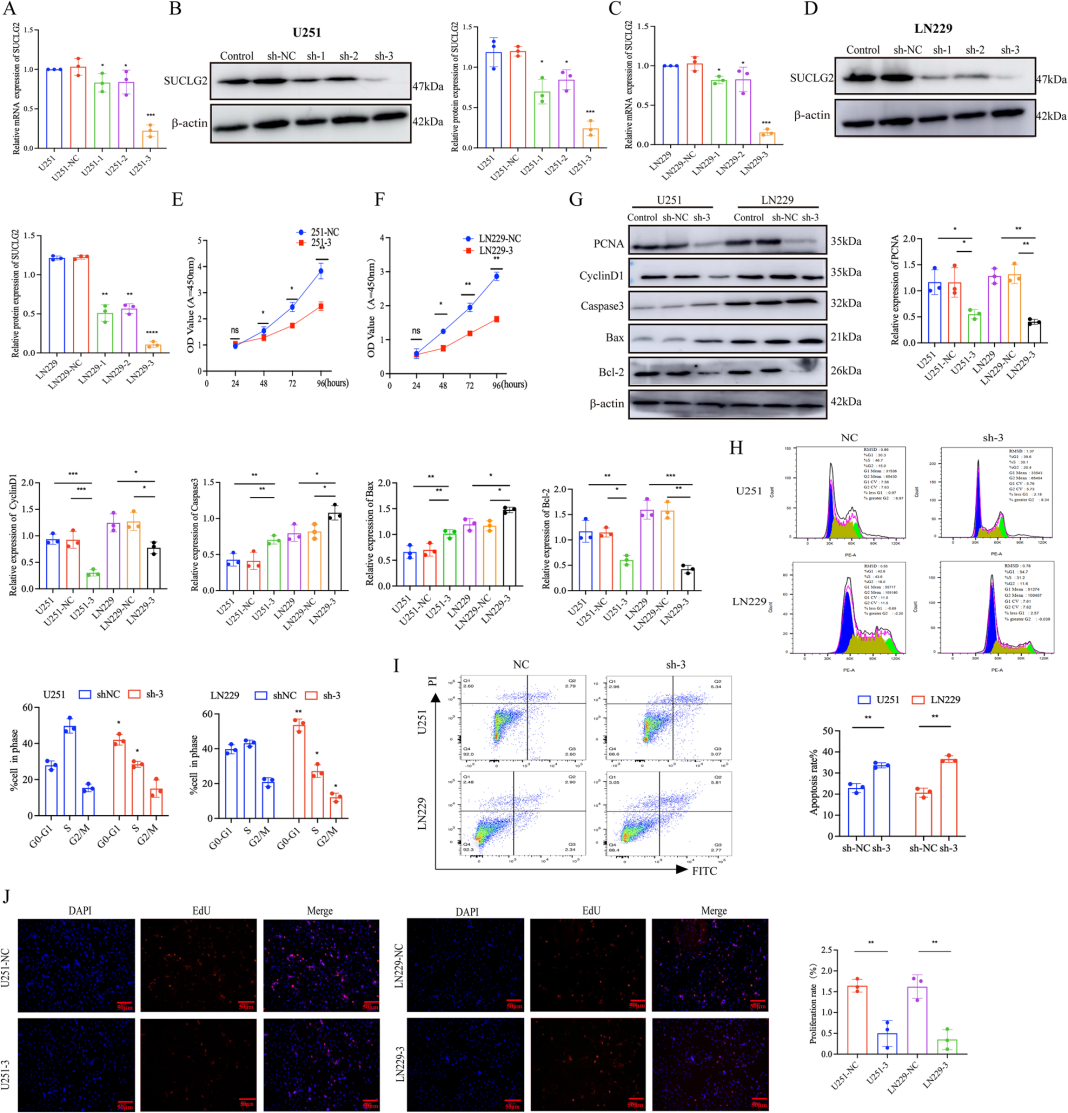
Knockdown of SUCLG2 modulates proliferation and apoptosis in GBM cells. **A**–**D** Extracts from U251 and LN229 cells stably expressing shNC or three independent shRNAs targeting SUCLG2 were used for western blotting and RT-qPCR. **E**, **F** The proliferative status of cells from U251 and LN229 stable cell lines expressing shNC or shRNAs, as revealed by CCK-8 assay. **G** Expression levels of PCNA, Cyclin D1, Bcl-2, Bax, and Caspase-3 in cells expressing shNC or shRNAs in U251 and LN229 stable cell lines, as revealed by the western blotting analysis. **H** Cell cycle changes were evaluated using flow cytometry on stable cell lines U251 and LN229 expressing shNC or shRNA. **I** Apoptosis levels in U251 and LN229 cells expressing shNC or shRNA were analysed using flow cytometry. **J** Stable expression of shNC or shRNA in U251 and LN229 cells was assayed for the level of DNA replication using EdU. *P < 0.05, **P < 0.01, ***P < 0.001.

We systematically evaluated SUCLG2’s effects on the proliferation and apoptosis of GBM cell lines U251 and LN229 to investigate its biological role in GBM progression. We first constructed SUCLG2-knockdown GBM cell lines using specific short hairpin RNAs (shRNAs) to deplete the SUCLG2 protein in LN229 and U251 cells. RT-qPCR and western blot analysis demonstrated that SUCLG2 expression levels were significantly reduced in SUCLG2-knockdown U251 and LN229 cells compared to those in wild-type cells (Fig. 2A–D).

Using the Cell Counting Kit-8 (CCK-8) assay, we assessed the cell viability of SUCLG2-knockdown LN229 and U251 cells to investigate the effects of SUCLG2 on cell proliferation. SUCLG2 knockdown significantly inhibited the proliferative capacity of U251 and LN229 cells (Fig. 2E, F). Western blot analysis revealed that silencing SUCLG2 affected the expression of cell proliferation and apoptosis-related molecules in SUCLG2-knockdown and wild-type LN229 and U251 cells. The SUCLG2-knockdown group exhibited a significant reduction in the expression of proliferation markers (such as PCNA and cyclin D1) and the Bcl-2, whereas the Bax and Caspase-3 were upregulated in the group (Fig. 2G). Additionally, flow cytometry indicated that SUCLG2 knockdown led to a significant increase in the proportion of cells in the G1 phase, a shift in the G2/M phase, and a decrease in the S phase in U251 and LN229 cells (Fig. 2H). Apoptosis detection via flow cytometry further revealed that SUCLG2 silencing significantly increased the apoptosis rate in U251 and LN229 cells (Fig. 2I). We used the 5-ethynyl-2-deoxyuridine (EdU) assay to further investigate the effect of SUCLG2 on DNA replication and found that SUCLG2 knockdown markedly inhibited the DNA replication capacity of U251 and LN229 cells (Fig. 2J). These findings suggest that silencing SUCLG2 significantly impedes cell cycle progression in GBM cells, slowing tumour progression.

### SUCLG2 knockdown inhibits GBM growth in vivo

We conducted further studies using an intracranial in situ xenograft model with LN229 cells to validate the role of SUCLG2 in vivo. Compared with that in the wild type, silencing SUCLG2 significantly inhibited tumour growth (Fig. S2A, S2B) and prolonged the survival time of the mice (Fig. S2C). IHC revealed that the expression levels of Ki-67 and SUCLG2 were significantly higher in wild-type tumour tissues than in SUCLG2-silenced tissues. This finding substantiates the role of SUCLG2 in promoting tumour cell proliferation (Fig. S2D).

### Effect of SUCLG2 knockdown on mitochondrial metabolism and function in GBM

**Fig. 3:**
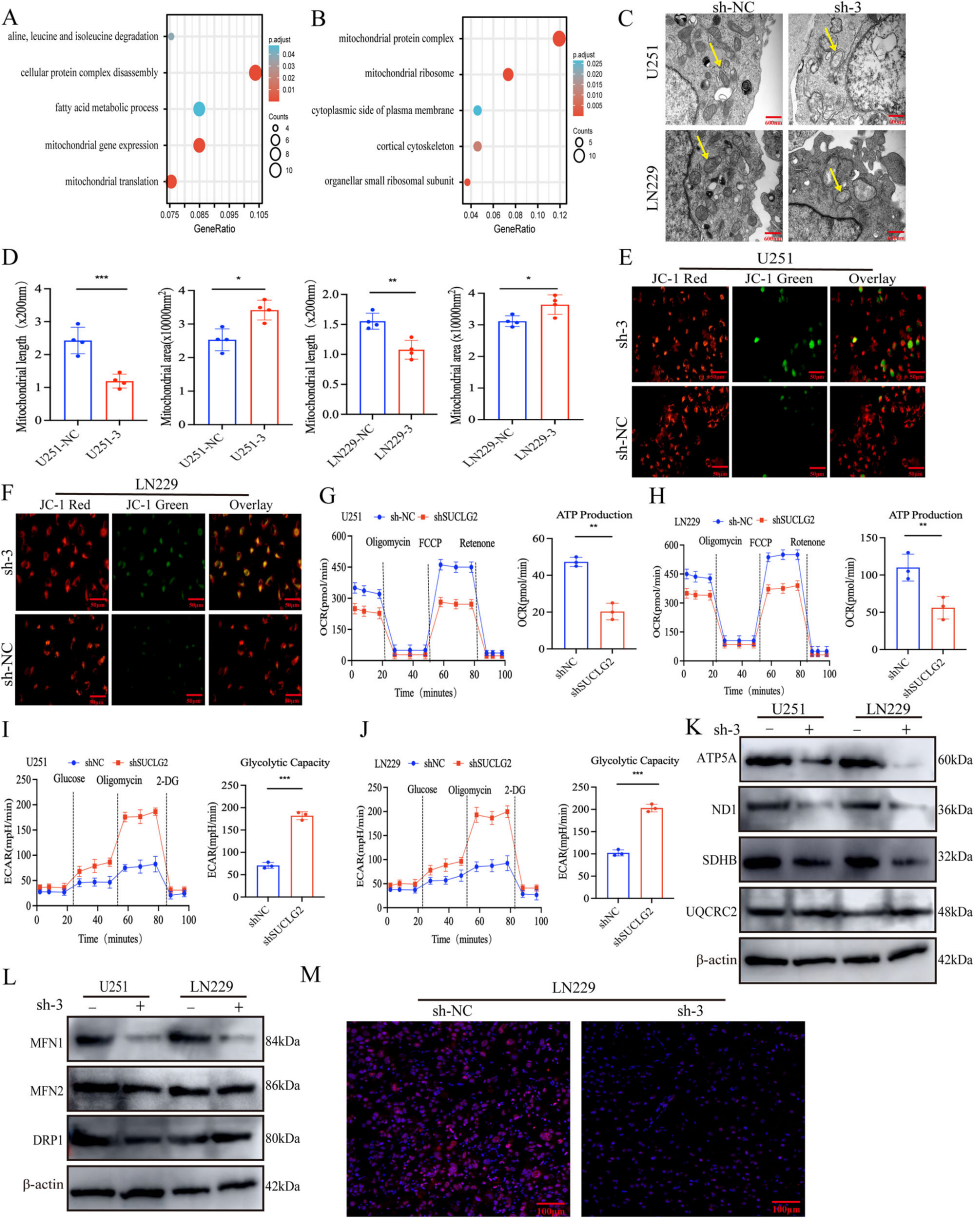
Effect of SUCLG2 knockdown on mitochondrial metabolism and function in GBM. **A** The Kyoto Encyclopaedia of Genes and Genomes (KEGG) pathway enrichment analysis of differentially expressed proteins in proteomics data from tumour periphery and tumour core. **B** Intracellular functions of differentially expressed proteins in the proteomics data of the tumour periphery and tumour core were analysed via Gene Ontology (GO) analysis. **C** Mitochondrial morphology changes were observed via electron microscopy in U251 and LN229 (SUCLG2-knockdown and control) cells. The mitochondria are indicated by yellow arrows. **D** Mitochondrial length and area were quantified using ImageJ software. **E**, **F** Fluorescence microscopy was performed to measure mitochondrial membrane potential changes after treatment with JC-1 solution. **G**–**J** Extracellular acidification rate (ECAR) and oxygen consumption rate (OCR) were measured in shNC- or sh-3-transfected U251 and LN229 cells (oligo: oligomycin; FCCP: carbonyl cyanide 4-(trifluoromethoxy)phenylhydrazone; Rot/Anti: rotenone and antimycin A). **K** Expression levels of the mitochondrial respiratory chain protein complexes ATP5A, SDHB, ND1, and UQCRC2 were analysed using western blotting. **L** Detection of MFN1, MFN2, and DPR1 expression using western blotting in SUCLG2-knockdown and wild-type U251 and LN229 cell lines. **M** L-lac staining and immunofluorescence distribution patterns of shNC versus sh-3 in intracranial tumour formation in LN229 cells. *P < 0.05, **P < 0.01, ***P < 0.001.

We analysed the differentially expressed proteins identified through proteomic analysis of the tumour periphery and core tissues to explore the anti-tumour mechanism of SUCLG2 in promoting the malignant progression of GBM. KEGG pathway analysis showed that the differentially expressed proteins were significantly enriched in biological processes such as cell protein complex degradation, fatty acid metabolism, and mitochondrial gene expression (Fig. 3A). Additionally, Gene Ontology analysis indicated that the upregulated proteins were significantly enriched in the mitochondrial protein complex pathway (Fig. 3B). These findings suggest that SUCLG2 may influence GBM progression through regulation of mitochondrial metabolism and function.

We examined the ultrastructures of the mitochondria in U251 and LN229 cells using transmission electron microscopy to assess the impact of SUCLG2 knockdown on the mitochondria. The mitochondrial volume and perimeter were significantly reduced in SUCLG2-knockdown cells, accompanied by small mitochondrial morphology, vacuolation, disorganised cristae structures, and mitochondrial disappearance (Fig. 3C, D). We used the JC-1 fluorescent probe to measure the mitochondrial membrane potential for further validation of the alterations in mitochondrial function. In the SUCLG2 knockdown group, green fluorescence signals significantly increased, indicating a significant decrease in mitochondrial membrane potential (Fig. 3E, F).

We used a Seahorse XF96 analyser to evaluate the metabolic levels of the cells for a comprehensive assessment of the impact of SUCLG2 knockdown on cellular energy metabolism. SUCLG2-knockdown LN229 and U251 cells exhibited significantly decreased oxygen consumption rate (OCR). OCR analysis revealed decreased basal and maximal mitochondrial respiratory capacities, accompanied by a gradual decline in adenosine triphosphate (ATP) production (Fig. 3G, H). Conversely, the extracellular acidification rate (ECAR) showed enhanced glycolytic and glycolytic reserve capacities (Fig. 3I, J). Consequently, we hypothesised that mitochondrial fragmentation causes metabolic dysfunction. Furthermore, examination of the expression levels of the mitochondrial core protein oxidative phosphorylation complex revealed that the mitochondrial proteins succinate dehydrogenase complex iron sulfur subunit B (SDHB), ND1, and ATP5A exhibited significantly lower expression in SUCLG2-knockdown cells than in the control group (Figs. 3K, S2E). MFN1, MFN2, and Drp1 analyses demonstrated that the expression of the mitochondrial fusion protein MFN1 was decreased in the SUCLG2-knockdown group, triggering mitochondrial apoptosis (Figs. 3L, S2F). These results indicate that SUCLG2 knockout leads to mitochondrial fragmentation by affecting MFN1 and the oxidative phosphorylation level. Immunofluorescence analysis revealed a marked reduction in lactate levels within tumour tissues following SUCLG2 knockdown, further elucidating the impact of SUCLG2 knockdown on mitochondrial metabolism (Fig. 3M).

### SUCLG2 causes limited oxidative phosphorylation and mitochondrial dysfunction through LMNA acetylation modification

**Fig. 4:**
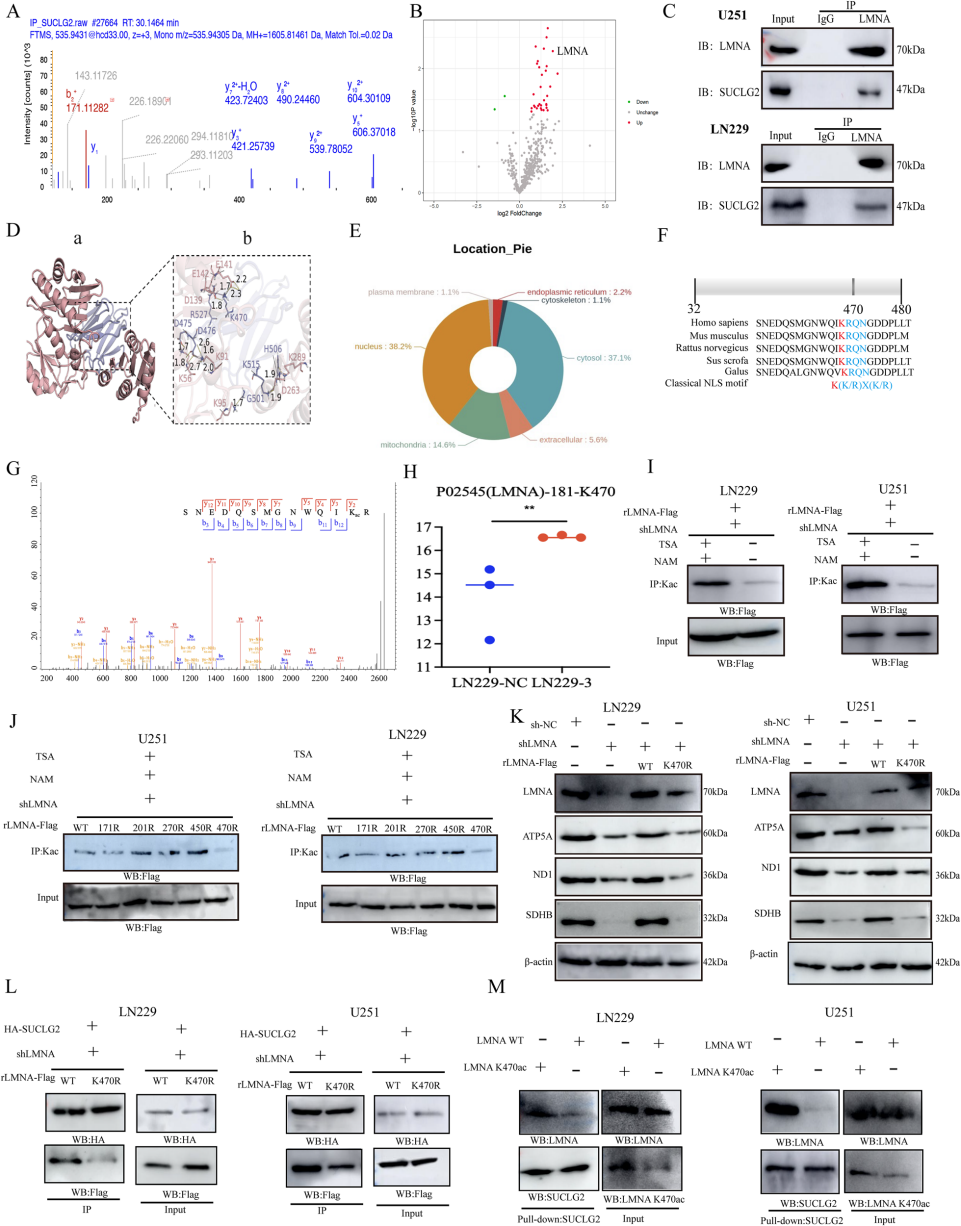
SUCLG2 causes limited oxidative phosphorylation and mitochondrial dysfunction through LMNA acetylation modification. **A** Representative mass spectrometry (MS) spectrum obtained by SUCLG2 immunoprecipitation using LN229 cells. **B** 4D acetylation modification proteomics using shNC or shRNA in LN229 cells to analyse differentially upregulated or downregulated proteins by acetylation modification. **C** Endogenous analysis of LMNA interaction with SUCLG2 using immunoprecipitation (IP) and immunoblotting (IB) assays. **D** a. Molecular docking of compound SUCLG2 with the structural domain of the LMNA enzyme. b. Generated a two-dimensional interaction map showing hydrogen bonding (yellow dashed line). **E** Subcellular localisation of acetylation-modified LMNA in cells with analysis of mitochondrial, cytoplasmic, and nuclear localisation. **F** The putative nuclear localisation signal (NLS) of LMNA is aligned across species. The putative NLS is highlighted in blue and red, with the strictly conserved residue K470 shown in red. **G** MS showing the amino acid sites at which LMNA undergoes acetylation modification. **H** Differential analysis of shNC versus shRNA in LN229 cells for acetylation proteomics analysis of LMNA acetylation at the K470 site. **I** LN229 cells stably expressing shLMNA were infected with lentiviruses expressing the rLMNA-FLAG WT, and lysates were immunoprecipitated with an anti-FLAG antibody, followed by western blotting. **J** LN229 cells were depleted of LMNA, and single-amino acid residues mutated in LMNA (K171, K201, K270, K450, K470) were analysed via protein blotting to detect LMNA protein expression. **K** LMNA was depleted in LN229 and U251 cells, and the expression of rLMNA-FLAG WT or K470R was rescued for western blotting analysis. **L** LMNA-depleted LN229 and U251 cells were transiently transfected with HA-SUCLG2 and rLMNA-FLAG WT or K470 R, followed by IP with an anti-FLAG antibody and western blotting. **M** LMNA K470 acetylated and LMNA WT were immunoprecipitated and purified from LN229 and U251 cells with an anti-LMNA K470ac or anti-SUCLG2 antibody, respectively. FLAG-SUCLG2 in vitro pull-down assays were performed using equal amounts of K470 acetylated LMNA K470 and LMNA WT. Cells were treated with tretinoin A (TSA, 10 mM) and nicotinamide (NAM, 10 mM) for 16 h. *P* < 0.05, **P < 0.01, ***P < 0.001.

To investigate the molecular mechanisms through which SUCLG2 influences mitochondrial dysfunction in GBM, we used immunoprecipitation (IP) to enrich SUCLG2-related proteins and subsequently identified their interacting partners using mass spectrometry (MS). We identified 164 proteins interacting with SUCLG2 (Fig. 4A). Subsequently, we conducted 4D label-free quantitative acetylation proteomics analysis on wild-type and SUCLG2-knockdown LN229 cells. The analysis revealed 34 proteins with significant acetylation modifications (fold change > 1.5, *P* < 0.05) in three replicate samples from the SUCLG2-knockdown group compared with those in the shNC group (Fig. 4B). Based on the IP-MS and acetylated proteomics results, we selected LMNA as a candidate target protein because of its detection in SUCLG2 IP-MS but not in control IP-MS and its identification in IP-MS and 4D acetylation proteomics; moreover, enrichment analysis of proteins with upregulated acetylation modifications in 4D acetylation proteomics showed that these proteins mainly affected ATPase activity in the TCA, playing a key role in maintaining cell survival and tumour progression (Fig. S3A). Furthermore, a Co-IP assay confirmed the endogenous interaction between SUCLG2 and LMNA (Fig. 4C), which was not observed with control IgG. Additionally, confocal microscopy demonstrated that SUCLG2 and LMNA were intracellularly colocalised (Fig. S3B).

Semi-flexible docking was conducted using HADDOCK 2.4 in the molecular docking model of SUCLG2 and LMNA. The binding interface included the α3 helix of LMNA (residues Lys470-Arg527) and β2 lamellae of SUCLG2 (residues Asp139-Glu142). The basic patches of LMNA (Arg527, Lys470) formed a charge-complementary interface with the acidic grooves of SUCLG2 (Asp141, Glu129, and Asp142), resulting in four pairs of salt bridges (e.g. Arg23-Asp101 and Lys47-Glu104) and a network of four hydrogen bonds (e.g. Lys470-Asp141) (Fig. 4D).

Lysine acetylation, as a reversible post-translational modification, regulates intermediate metabolic-enzyme function and participates in the core regulation of metabolic pathways by altering enzyme activity, stability, subcellular localisation, and protein–protein interactions [18, 19]. We analysed the subcellular localisation of LMNA and found that it was abundant in the cytoplasm (37.1%), nucleus (38.2%), and mitochondria (14.6%) (Fig. 4E). Quantitative and differential analyses of 4D acetylation sites revealed LMNA acetylation at the amino acid residues K171, K201, K270, K450, and K470, with significantly elevated acetylation observed at K470. Additionally, the amino acid sequence of the acetylated region was presented via secondary MS (Fig. 4G). The analyses of wild-type and SUCLG2-knockdown cells revealed that the K470 site of LMNA exhibited significantly elevated acetylation under SUCLG2 knockdown (Fig. 4H). To confirm this, we treated LN229 and U251 cells with histone deacetylase inhibitors and observed enhanced LMNA acetylation (Fig. 4I). Further comparative analysis of the amino acid sites and sequences near the MS-acquired amino acid residue K470 among different species demonstrated a high degree of consistency in this sequence region, indicating that the amino acid sequences perform the same function across species (Fig. 4F).

Subsequently, we generated constructs with mutations in each of the acetylated lysine residues (K171, K201, K270, K450, and K470) in LMNA. Protein blotting indicated that the K470R mutation significantly reduced LMNA acetylation (Fig. 4J). Next, we investigated whether K470 acetylation would influence the expression levels of mitochondrial proteins MFN1, ATP5A, SDHB, and ND1. Western blot analysis revealed that the rLMNA K470R mutation markedly decreased MFN1, ATP5A, SDHB, and ND1 levels in U251 and LN229 cells (Fig. 4K). Additionally, IP demonstrated that the K470R mutation disrupted the interaction between SUCLG2 and LMNA (Fig. 4L); the interaction was enhanced following treatment with the histone deacetylase inhibitors nicotinamide (NAM) and tretinoin A (Fig. S3C).

This result was corroborated by an in vitro FLAG-SUCLG2 pull-down assay, demonstrating that, in the immunoprecipitated complexes, the SUCLG2 isolated using a specific antibody targeting LMNA K470ac was more prevalent than that isolated with an anti-LMNA antibody (Fig. 4M). Our findings indicate that LMNA K470ac is essential for the formation of the SUCLG2/LMNA complex, regulation of oxidative phosphorylation levels in the inner mitochondrial membrane, and suppression of MFN1 expression in the outer membrane.

### SUCLG2 knockdown reduces lactate metabolism in GBM

Following intracranial implantation of LN229 cells, it was demonstrated that knocking down SUCLG2 significantly reduced lactate concentrations within tumour tissue (Fig. 3M). Moreover, hypoxia-inducible factor 1-alpha (HIF-1α) facilitates glucose uptake and lactate production by binding to glycolysis-related genes (e.g., *Glut1*, *HK2*, *LDHA*, and *PKM2*), enhancing glycolytic activity [20].

**Fig. 5:**
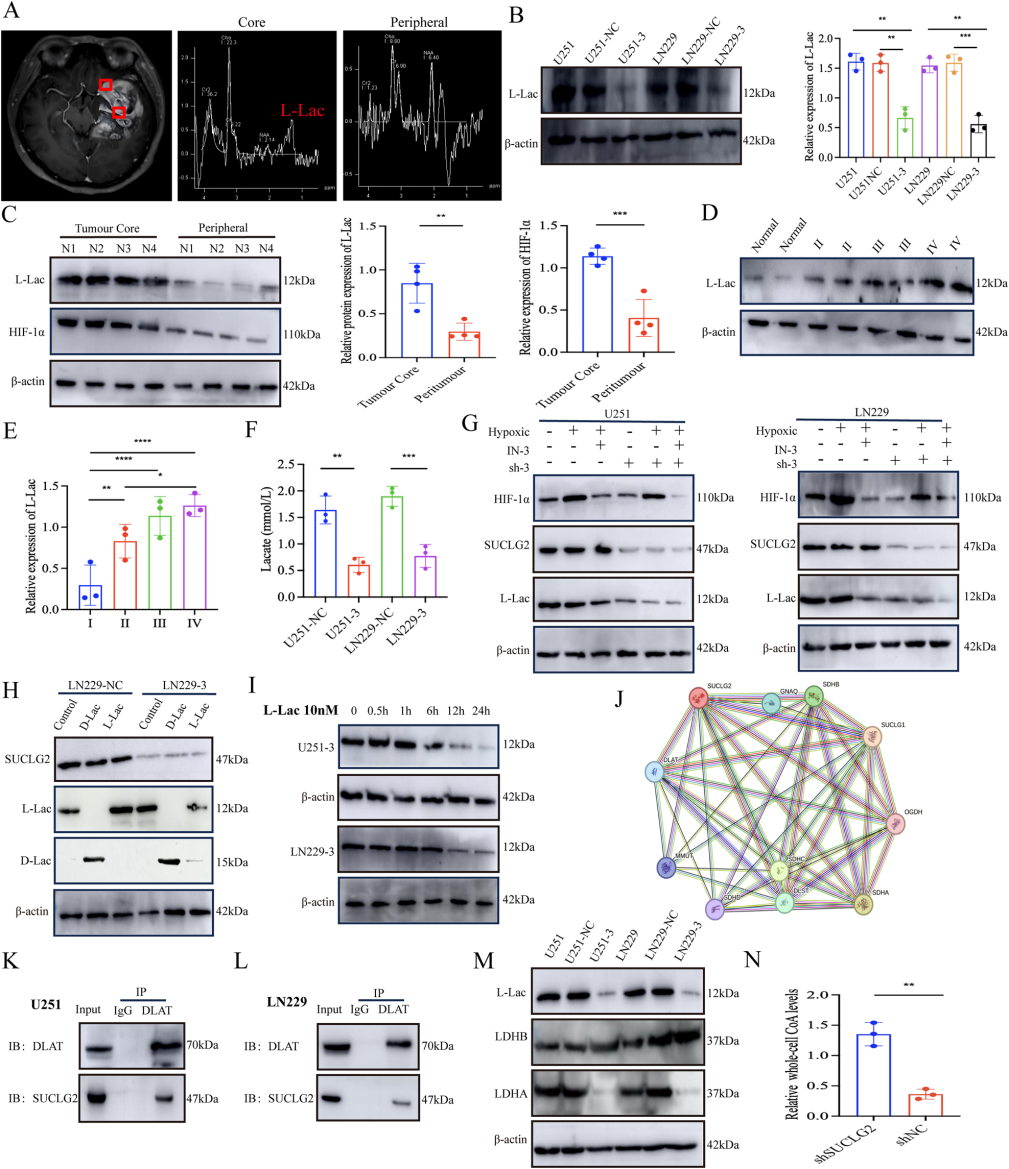
SUCLG2 knockdown reduces lactate metabolism in GBM. **A** Clinical GBM magnetic resonance imaging shows the lactate peak spectrum analysis of the tumour periphery and core. **B** U251 and LN229 showing shRNA vs. shNC in the western blot analysis of L-Lac expression. **C** L-Lac and HIF-1α expression levels were analysed in GBM clinical peripheral and core tissue specimens using western blotting (*n* = 4). **D**, **E** Brain injury tissue specimens and those of different GBM grades were analysed for L-Lac expression using western blotting, normal (*n* = 4), Grade II (*n* = 4), Grade III (*n* = 4), and Grade IV (*n* = 4). **F** L-Lac content was determined in U251 and LN229 shNC or shRNA cells via enzyme-linked immunosorbent assay. **G** shRNA or shNC of U251 and LN229 cells were subjected to hypoxic intervention for 72 h and treated with the HIF-1α inhibitor IN-3 to evaluate the expression of HIF-1α, SUCLG2, and L-lac using western blotting. **H** LN229 cells transfected with shNC or shRNA were treated with 10 nM L-Lac and 10 nM D-Lac for 12 h, and the expression levels of SUCLG2, L-Lac, and D-Lac were assessed via western blotting. **I** U251 and LN229 cells transfected with shSUCLG2 were treated with 10 nM L-Lac, and cell pellets were collected at 0.5, 1, 6, 12, and 24 h. L-Lac expression levels were detected using western blotting. **J** The prediction of protein–protein interaction (PPI) network with SUCLG2 was performed through online PPI analysis. Each node in the figure represents a protein. Different isoforms from the same gene were merged. The spiral structure inside the nodes indicates the three-dimensional structure of the protein. Red represents the query protein. As shown in the figure, the differently coloured lines between two proteins represent different types of interaction evidence. Among the interactions, some are experimentally validated, while red, blue, and green represent predicted interaction results. **K**, **L** Analysis of the interaction between DLAT and SUCLG2 through endogenous Co-IP. **M** The expression of DLAT, L-Lac, LDHA, and LDHB was analysed by Western blotting in LN229 and U251 cells containing shNC and shRNA. **N** The content of acetyl-CoA in LN229 cells containing shNC and shRNA was determined by mass spectrometry. *P < 0.05, **P < 0.01, ***P < 0.001.

We used GBM magnetic resonance spectroscopy imaging to compare the metabolic characteristics of the tumour core and peripheral regions for further probing of the regulatory role of SUCLG2 in metabolism. The lactate peak was significantly higher in the core region than in the peripheral region (Fig. 5A), suggesting a greater degree of hypoxia and lactate accumulation in the core region. Subsequently, we examined the expression levels of L-Lac in SUCLG2-knockdown and wild-type U251 and LN229 cell lines via western blot analysis and found significant reduction in L-Lac levels in the SUCLG2-knockdown cells (Fig. 5B). Western blot analysis of GBM clinical samples, tumour core tissues, and peritumoural tissues showed high concordance with the magnetic resonance spectroscopic findings (Fig. 5C). Furthermore, lactate assays conducted on different grades of GBM tissues revealed no significant differences between Grades II and III; however, both grades exhibited significantly higher levels than did normal tissues (Fig. 5D, E).

We cultured SUCLG2-knockdown LN229 and U251 cells and wild-type controls for 96 h to investigate the role of L-Lac in GBM. We measured the cytoplasmic lactate levels using an enzyme-linked immunosorbent assay and observed a significant reduction in L-Lac levels in the knockdown group (Fig. 5F). To elucidate the mechanism underlying lactate reduction following SUCLG2 knockdown, SUCLG2-knockdown LN229 and U251 cells and wild-type controls were treated with IN-3 and subjected to hypoxic conditions for 72 h, followed by western blot analysis. HIF-1α expression was significantly elevated under hypoxic conditions, whereas L-Lac expression did not exhibit significant changes after SUCLG2 knockdown (Fig. 5G). This finding suggests that HIF-1α regulates L-Lac production but does not influence SUCLG2 expression. The observed decrease in L-Lac levels may be attributed to the enhanced metabolic consumption of L-Lac due to SUCLG2 knockdown. To verify this, we administered L-Lac and D-Lac to LN229 and U251 cells in the control and knockdown groups. D-Lac levels were significantly higher than L-Lac levels, suggesting that SUCLG2-knockdown facilitated the metabolism of L-Lac but not that of D-Lac (Fig. 5H). To further elucidate the temporal dynamics of L-Lac metabolism, SUCLG2-knockdown LN229 and U251 cells were treated with 10 nM L-Lac, and cellular protein samples were collected at 0.5,1, 6, 12, and 24 h. Western blot analysis revealed a significant decrease in L-Lac levels at the 12-h mark (Fig. 5I). These findings indicate that SUCLG2 knockdown effectively inhibits tumour progression by reducing lactate levels in tumour cells.

A protein interaction network diagram was constructed for the SUCLG2 target protein based on the STING database to extensively investigate the mechanism of lactate metabolism. SUCLG2 is primarily enriched in mitochondrial metabolic enzymes associated with the TCA cycle, with which it interacts. Based on functional predictions, DLAT catalyses the conversion of pyruvate to acetyl-CoA, and SUCLG2 may exert its lactate-lowering effect through DLAT interaction (Fig. 5J). Endogenous validation further confirmed the binding of SUCLG2 to DLAT (Fig. 5K, L). Additionally, western blot analysis, conducted to assess the L-Lac, LDHA, and LDHB levels in wild-type and SUCLG2-knockdown LN229 and U251 cells, revealed a significant decrease in LDHA expression and an increase in LDHB expression. Notably, DLAT expression in LN229 cells was significantly increased, and LDHB showed increased expression (Figs. 5M, S3D). Quantitative analysis of whole-cell acetyl-CoA via MS in LN229 cells indicated that the shNC group exhibited significantly lower levels of acetyl-CoA than did the SUCLG2-knockdown cells (Fig. 5N). These results suggest that SUCLG2 plays a crucial role in regulating lactate metabolism and participates in the metabolic reprogramming of GBM, potentially inhibiting the progression of GBM by affecting DLAT expression and acetyl-CoA production.

### L-Lac from anaerobic glycolysis promotes GBM malignant progression through metabolic reprogramming

**Fig. 6:**
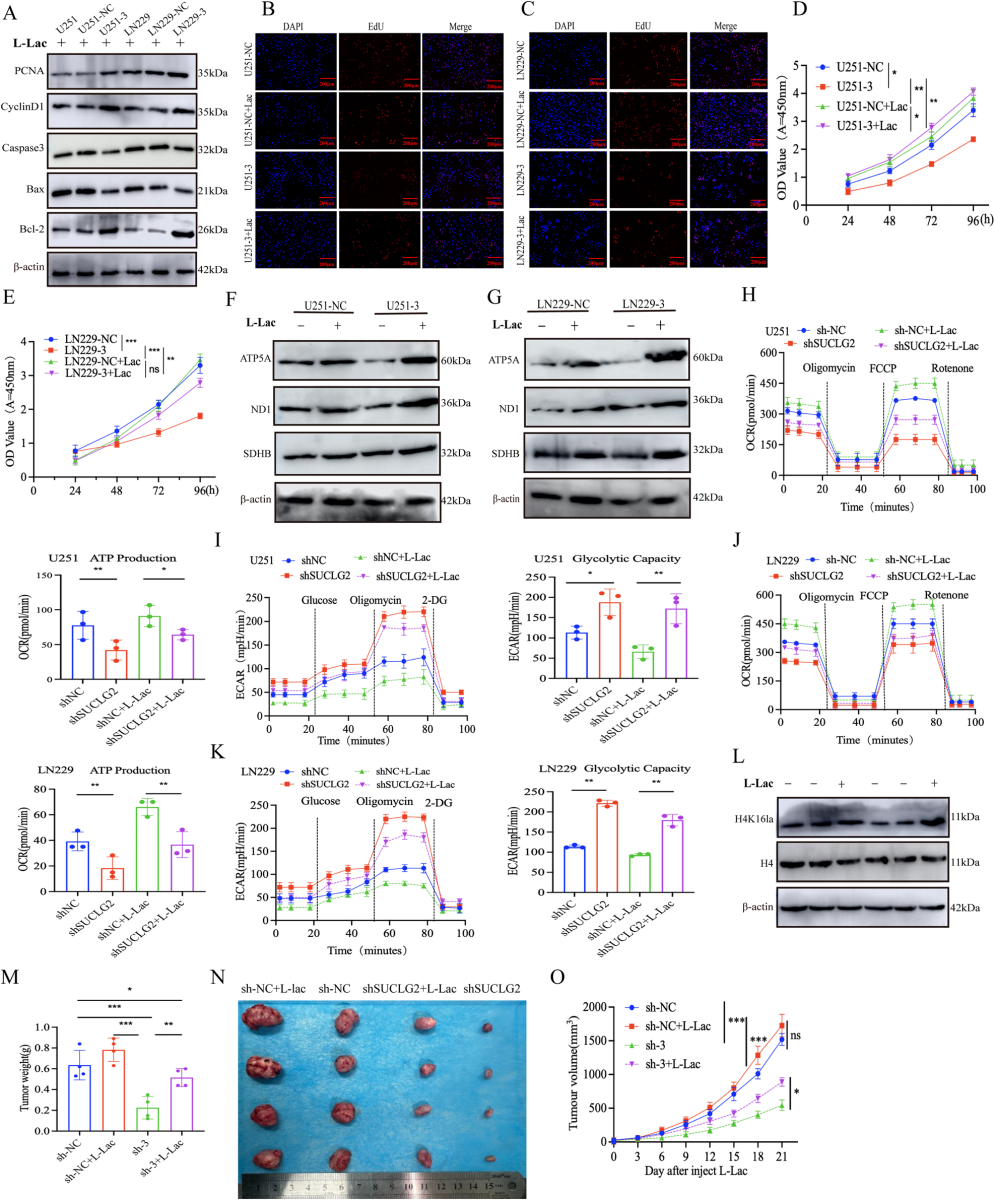
L-Lac from anaerobic glycolysis promotes GBM malignant progression through metabolic reprogramming. **A** U251 and LN229 cells containing shNC and shRNA were cultured with 10 nM L-Lac for 6 h, and the expression levels of PCNA, Bcl-2, Caspase-3, Bax, and CyclinD1 were analysed by Western blotting. **B**, **C** U251 and LN229 cells containing shNC and shRNA were subjected to EdU experiments 6 h after intervention with 10 nM L-Lac. **D**, **E** U251 and LN229 cells containing shNC and shRNA were treated with 10 nM L-Lac in excess, and the optical density change was measured using CCK-8 six hours after intervention. **F**, **G** U251 and LN229 cells containing shNC and shRNA were treated with 10 nM L-Lac for 6 h, followed by western blotting analysis of changes in ATP5A, ND1, and SDHB. **H**–**K** Cell culture procedure over the addition of 10 nM L-Lac for 6 h. The ECAR and OCR of shNC- or sh-3-transfected U251 and LN229 cells were measured using the Mito Stress Test kit (*N* = 3) (Oligo: Oligomycin; FCCP: Carbonyl Cyanide 4-(Trifluoromethoxy)Phenylhydrazone; Rot/Anti: rotenone and antimycin A). **L** Following the addition of 10 nM L-lac, Western blotting analysis was performed to detect the expression of H4 and H4K16la. **M**–**O** After subcutaneous implantation of LN229 cells in nude mice, shNC and shRNA were treated with L-Lac combination therapy, and tumour volume, weight, volume, and growth rate were measured at the time of euthanasia (*n* = 4 per group). *P < 0.05, **P < 0.01, ***P < 0.001.

GBMs utilise several carbon compounds, with glucose being the primary source. Lactic acid was initially considered a waste product of glucose catabolism; however, it readily accumulates in tumour tissues, reaching ~40 mM [21, 22]. Notably, 10–15 mM lactate significantly reverses the loss of cell viability under low-glucose conditions [23, 24]. We treated the control and SUCLG2-knockdown groups of U251 and LN229 cells with 10 nM L-Lac to investigate the metabolic profile of L-Lac in the TME. Western blot analysis showed that following L-Lac treatment, the expression levels of proliferation markers PCNA and cyclin D1 and of the Bcl-2 in GBM cells were significantly upregulated, whereas those of the Bax and Caspase-3 were significantly downregulated (Figs. 6A, S3E). Additionally, cell cycle analysis indicated that the control and SUCLG2-knockdown LN229 and U251 cells treated with L-Lac exhibited a significant increase in the proportion of S-phase cells. Furthermore, after L-lactate treatment, the EdU fluorescence intensity was significantly higher in the SUCLG2-knockdown group than in the knockdown group without lactic acid addition, as revealed by the EdU assay (Fig. 6B, C). The CCK-8 assay further confirmed that L-Lac treatment significantly promoted the proliferation of the control cells (Fig. 6D, E). Additionally, L-Lac effectively reversed nutrient deficiency-induced apoptosis in a concentration-dependent manner. Under L-Lac treatment, metabolism significantly promoted the production of citrate, succinate, fumarate, and malate, as demonstrated by the U-13C-L-Lac carbon-tracking assay [21]. Therefore, L-Lac, as a significant source of mitochondrial energy, participates in the energy metabolism of tumour tissues, promotes tumour cell proliferation, and inhibits apoptosis by altering the TME.

Following SUCLG2 protein knockdown, the expression levels of ATP5A, ND1, and SDHB proteins were significantly lower in LN229 and U251 cells than in the knockdown group supplemented with lactic acid (Figs. 6F, G, and S3F). Furthermore, we administered L-Lac within 6 h and assessed the metabolic levels of SUCLG2-knockdown LN229 and U251 cells using a Seahorse XF96 analyser. The ECAR, glycolytic capacity, and glycolytic reserve capacity were significantly reduced compared with those in the group without L-Lac treatment. Conversely, the OCR was significantly higher than that in the group without L-Lac, indicating elevated basal and maximal mitochondrial respiratory capacities, alongside increased ATP production (Fig. 6H–K). Furthermore, the addition of lactic acid resulted in elevated levels of H4K16la, a lactate-modified histone, while no significant alterations were observed in H4 (Fig. 6L). The mitochondrial membrane potential was evaluated using the JC-1 fluorescent probe, which revealed that the membrane potential in SUCLG2-knockdown cells was diminished in the green channel and enhanced in the red channel following L-Lac treatment (Fig. S4A). Notably, MFN1 expression was significantly elevated in the shSUCLG2 group after L-Lac treatment (Fig. S4B). We hypothesised that following SUCLG2 knockdown, the addition of L-Lac acid would promote mitochondrial fusion by activating alternative metabolic pathways.

We administered L-Lac for 21 days after subcutaneous tumour inoculation in nude mice to investigate the role of L-Lac in vivo. The results indicated that the tumour growth rate, weight, and volume in the shSUCLG2+L-Lac group were significantly higher than those in the shSUCLG2+saline group (Fig. 6M, N). Conversely, no significant differences were found between the shNC+L-Lac and shNC+saline groups (Fig. 6O). These findings suggest that L-Lac significantly promotes the malignant progression of GBM through metabolic reprogramming, providing a critical foundation for the further exploration of therapeutic strategies that target lactate metabolism.

### Lactate regulates H4K16la binding to promoter regions and cis-regulatory elements and affects chromatin accessibility

**Fig. 7:**
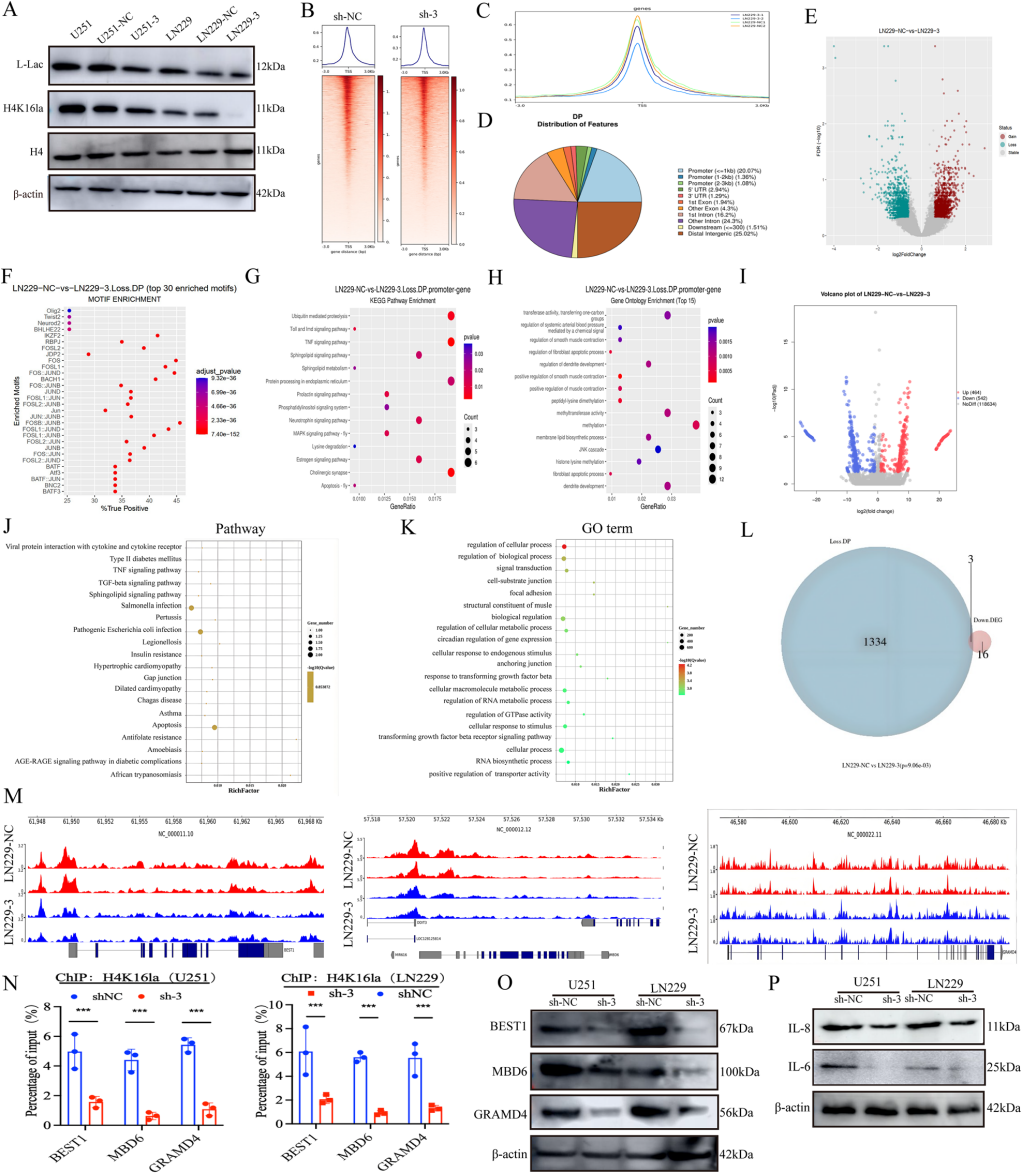
Lactate regulates H4K16la binding to promoter regions and cis-regulatory elements and affects chromatin accessibility. **A** H4K16la and L-Lac expression patterns in shNC and shSUCLG2 cells of U251 and LN229 were determined using western blotting. **B** Cell precipitates were collected for cleavage under targets and tagmentation (CUT&Tag) assay to identify H4K16la binding peaks, and the heatmap shows the distribution of H4K16la peaks near the translation start site (TSS). **C** Comparison of shNC and shSUCLG2 peaks in LN229 cells. **D** Genomic distribution of H4K16la, which was annotated for its localisation (promoter, exon, intron, or intergenic) and quantified in relative and absolute amounts. **E** Volcano plots of upregulated versus downregulated genes after CUT&Tag assay for H4K16la binding. **F** The heat map shows genes downregulated around TSS in LN229 cells containing shNC and shSUCLG2 as detected by the CUT&Tag assay. **G** KEGG analysis of CUT&Tag-detected downregulated genes at H4K16la. **H** GO analysis of CUT&Tag-detected downregulated genes. **I** Volcano plot of differential gene expression in RNA-seq. **J** KEGG analysis of downregulated genes in shSUCLG2 versus shNC using RNA-seq. **K** GO analysis of shSUCLG2 versus shNC downregulated genes using RNA-seq. **L** Combined CUT&Tag and RNA-seq analyses were performed to identify potential downstream target intersections of H4K16la. **M** Integrated genomics viewer trajectory track of CUT&Tag data showing enrichment of H4K16la at BEST1, GRAMD4H, and MBD6 promoter regions. **N** LN229 and U251 cells were treated with shNC or shRNA, and DNA fragments were immunoprecipitated with H4K16la antibody and analysed by qPCR. **O** Representative western blotting images show the quantification of BEST1, MBD6, and GRAMD4 protein levels. **P** Western blotting of IL-6 and IL-8 in shNC or shRNA lines of LN229 and U251. *P < 0.05, **P < 0.01, ***P < 0.001; ns, not significant.

We first assessed the level of histone lactylation modification (H4K16la) using western blot analysis to further investigate the mechanism through which SUCLG2 regulates L-Lac and subsequently affects GBM progression through epigenetic modification. The results indicated a significant reduction in H4K16la expression in the shSUCLG2 group (Figs. 7A, S4C). We conducted cleavage under targets and tagmentation (CUT&Tag) experiments using H4K16la antibodies to further elucidate the role of H4K16la in gene regulation. Analysing the sequencing data from the shSUCLG2 group and mapping them to the genome revealed a marked decrease in H4K16la binding at the transcription start site. We annotated and compared the peaks under both conditions. We found that the number of peaks in SUCLG2-knockdown cells was significantly lower (Fig. 7B, C), with ~20.07% enrichment observed in the promoter region (Fig. 7D).

CUT&Tag analysis demonstrated that SUCLG2-knockdown LN229 cells exhibited significant downregulation of differential genes at the H4K16la binding site (Fig. 7E, log2-fold change > 0.58, false discovery rate <0.05). We screened potential target genes for lactylation regulation and identified 29,523 genes in LN229 cells, among which 36 were significantly downregulated (Fig. 7F). KEGG pathway analysis revealed that gene functions in the H4K16la promoter region related to the apoptosis pathway were significantly enriched (Fig. 7G). Additionally, Gene Ontology functional enrichment analysis indicated that these downregulated genes are primarily associated with apoptosis (Fig. 7H).

We conducted RNA sequencing on SUCLG2-knockdown and control LN229 cells to validate these findings. We detected 118,634 genes, among which 542 were significantly downregulated in the shSUCLG2 group (Fig. 7I). This finding aligns with the CUT&Tag analysis results. KEGG analysis demonstrated their association with the regulation of cellular processes (Fig. 7J), whereas Gene Ontology analysis indicated that the downregulated genes are primarily involved in apoptotic processes (Fig. 7K). By integrating CUT&Tag and RNA-sequencing data enriched in the apoptotic pathway, we identified three differentially expressed genes (*BEST1*, *GRAMD4*, and *MBD6*) significantly downregulated following H4K16la regulation (Fig. 7L). These genes exhibited reduced mRNA levels and a significant decrease in H4K16la signalling enrichment in their promoter regions in LN229 cells, whereas they were upregulated in the shNC group (Fig. 7M). GRAMD4 acts as a mitochondrial effector and is crucial in E2F1-induced apoptosis, particularly in the context of p53/TP53 deletion [25]. BEST1 participates in glioma proliferation by regulating synaptic plasticity, potentially enhancing neuronal signal reception and tumour cell proliferation through synaptic remodelling [26]. MBD6, as a non-catalytic component of the polycomb repressive deubiquitinating enzyme complex, participates in the epigenetic regulation of gene expression, influencing cell proliferation and survival and promoting cancer cell growth [27].

We examined the enrichment of H4K16la in the promoter regions of *BEST1*, *GRAMD4*, and *MBD6* using ChIP-qPCR and western blot analysis to verify the regulatory mechanisms of these genes (Figs. 7N, O, S4D). The peak of H4K16la in the promoter regions of these genes was significantly reduced following SUCLG2 knockdown. This indicates that the lactylation modification of H4K16la inhibits the transcription of *BEST1*, *GRAMD4*, and *MBD6*. Additionally, western blot analysis revealed that the expression levels of IL-6 and IL-8 were significantly reduced after SUCLG2 knockdown, which affected GBM (Figs. 7P, S4E). These results suggest that lactic acid influences chromatin accessibility and gene transcriptional activity by regulating the binding of H4K16la to promoter regions and cis-regulatory elements, modulating the proliferation and apoptosis of GBM cells.

## Discussion

GBM exhibits significant spatiotemporal heterogeneity owing to the interaction of the dynamic clonal evolution of tumour cells with their microenvironment. Genetic polymorphism is driven by high-frequency mitosis-induced subclonal differentiation, manifesting as spatiotemporal heterogeneity of genomes across different anatomical regions and within the same tumour [28]. This heterogeneity is regulated by genetic mutational stochasticity and microenvironmental selective pressures, leading to the formation of subclonal ecological niches conferring competitive growth advantages [29, 30]. Notably, each subclone may modify the TME to enhance adaptability through mechanisms such as angiogenesis induction, immune checkpoint modulation, and extracellular matrix remodelling. Multi-omic sequencing has revealed significant dynamic changes in the genetic composition of GBM across anatomical regions and temporal dimensions [31]. Accordingly, we primarily focused on GBM heterogeneity. Through proteomic sequencing, we screened for differentially expressed proteins and emphasised that cancer treatment should employ strategies to mitigate tumour heterogeneity by reducing lactate levels within the microenvironment. Concurrently, we intervened in the metabolic processes of tumour cells in the microenvironment to prevent potential future tumour progression.

Mitochondria, as the core organelles of cellular energy metabolism, play a pivotal role in the G2/M phase of the cell cycle and in DNA repair through their oxidative respiratory function [32, 33]. Increasing evidence indicates that tumour cells rely on glycolysis while reactivating oxidative phosphorylation to meet the repair and survival demands arising during anticancer therapy [34, 35]. Mitochondria are vulnerable to damage under various stress conditions, potentially leading to dysfunction, reduced ATP synthesis, and increased production of reactive oxygen species—changes closely associated with the pathophysiology of numerous diseases [36]. Moreover, mitochondria regulate nuclear gene expression through retrograde signalling, facilitating cellular adaptation to metabolic stress [37]. Consequently, targeting mitochondrial retrograde signals—such as mitochondrial reactive oxygen species, tumour metabolites, and mitochondrial calcium ions—has emerged as a potential anti-cancer strategy [38, 39]. In our cellular model, the reduced expression of ATP5A, ND1, and SDHB was influenced by the interaction between SUCLG2 and LMNA, along with the acetylation modification of lysine residue K470. This interaction resulted in diminished mitochondrial membrane potential, decreased ATP production, and degradation of MFN1, subsequently altering the cell cycle and triggering mitochondrial apoptosis. Furthermore, the reduced levels of L-lactic acid slow tumour progression through histone H4K16 lactylation and regulate the expression of the *BEST1*, *GRAMD4*, and *MBD6* genes, with this mitochondrial effector serving as an apoptotic signal induced by E2F1.

A mitochondrial protein may interact with multiple other proteins to perform various functions. Previous studies have reported that isoforms of the same mitochondrial protein are allocated to different mitochondrial sub-compartments, with certain proteins potentially exhibiting multiple cellular localisations to function efficiently [40]. Our findings demonstrated that SUCLG2 interacts with LMNA, impairing oxidative phosphorylation, whereas its interaction with DLAT reduces lactate levels in the TME. This further elucidates the protein contact sites involved in metabolic regulation with temporal and spatial specificity, introducing a novel complementary mechanism in tumour metabolism. Under these conditions, SUCLG2/LMNA interactions significantly compromise mitochondrial integrity, whereas SUCLG2/DLAT interactions in certain mitochondria diminish lactate-regulated H4K16la expression and exacerbate mitochondrial stress damage. However, further research is required to elucidate this mechanism.

Therapeutic strategies targeting tumour-cell metabolism have shown immense potential. Disrupting pathways such as glycolysis, the electron transport chain, glutamine metabolism, branched-chain amino acid metabolism, and fatty acid oxidation and synthesis has provided new perspectives for cancer treatment [41]. In hypoxic microenvironments, tumour cells produce substantial lactate by upregulating LDH activity, with LDHA catalysing the conversion of pyruvate to lactate to support hypoxic metabolism. LDHB promotes the reconversion of lactate to pyruvate under aerobic conditions [42, 43]. Mechanistic studies indicate that LDHA-mediated glycolysis drives GBM cell proliferation, survival, and resistance to radiotherapy and chemotherapy [44]. Additionally, clinical data have shown a positive correlation between serum lactate levels and glioma malignancy [45]. Our findings indicate that lactate levels in GBM tissues correlate with the WHO grading. We further validated lactate distribution for tumour treatment through clinical nuclear magnetic resonance spectroscopy. Additionally, SUCLG2 interacts with DLAT to promote the conversion of pyruvate to acetyl-CoA, while influencing the activity of the LDHA and LDHB enzymes through the substrate pyruvate, thereby gradually metabolising lactate and leading to a significant reduction in lactate levels. In vitro assays further demonstrated that L-Lac treatment markedly enhanced malignant tumour progression, whereas silencing of SUCLG2 led to a notable decrease in lactate levels, inhibiting tumour growth. Consequently, we propose a promising strategy facilitating the conversion of pyruvate to acetyl-CoA through the interaction between SUCLG2 and DLAT, while simultaneously modulating the activity of LDHA and LDHB to convert L-lactic acid to pyruvate, a process that plays a crucial role in inhibiting the proliferation of GBM cells.

Histone lactylation, a novel epigenetic modification, is regulated by intracellular lactate levels and directly stimulates gene transcription in chromatin [13]. Histone lactylation is crucial in biological processes such as macrophage polarisation and cellular reprogramming [14]. Moreover, it is closely associated with tumour development [46]. In head and neck squamous cell carcinoma, VEGF-A promotes tumour proliferation by upregulating BEST1 expression in monocytes and enhancing the secretion of cytokines such as IL-6 and IL-8 [47]. Accordingly, we showed that lactic acid inhibited *BEST1* expression by downregulating histone H4K16la, promoting stress injury in cells within the TME. Conversely, the downregulation of *GRAMD4* inhibited tumour progression by inducing mitochondrial apoptosis. Additionally, *MBD6*, a non-catalytic component of the polycomb repressive deubiquitinating enzyme complex, inhibited cancer cell growth by regulating cell proliferation and survival. Lactate plays a significant role in inhibiting GBM through metabolic reprogramming by acting as a key regulator of histone acetylation modification.

The core region of GBM is characterised by elevated SUCLG2 expression and an L-lac-rich microenvironment, which promotes malignant-tumour progression. Our findings indicate that SUCLG2 induces the acetylation modification of LMNA, causing a decrease in MFN1 expression, reducing oxidative phosphorylation levels, and triggering mitochondrial apoptosis. Additionally, interactions involving DLAT weaken lactate-mediated chromatin accessibility regulation, inhibiting GBM cell proliferation and regulating apoptosis. These findings suggest that SUCLG2 plays a central role in lactate-metabolism regulation and epigenetic regulation in GBM, offering new theoretical directions for targeted therapy and providing a solid theoretical foundation for multidimensional treatment strategies based on the metabolic–epigenetic axis.

## Materials and methods

### Study population and human specimens

This study included patients who underwent GBM resection in our institution between 2020 and 2023. GBM diagnosis was based on clinical presentation, laboratory tests, and imaging findings and was confirmed histologically by at least two senior pathologists blinded to the clinical diagnosis. Fresh GBM tissues were collected during surgical resection and sequenced for proteomic analysis. Twenty-four human GBM samples were collected, along with eight brain tissues from patients with traumatic brain injury and six normal brain tissue samples from patients who underwent fistula surgery. Paraffin-embedded tissues were subjected to IHC, protein blotting, and RT-qPCR.

### Cell culture and reagents

HA, U87MG, U251MG, LN229, and U118 glioma cell lines were obtained from the Chinese Academy of Sciences Cell Bank (Shanghai, China). U-251MG and LN229 cells were cultured in a high-glucose Dulbecco’s modified Eagle’s medium (PM150210; Pernos Life Sciences, Wuhan, China) in a 4:1 medium containing 10% foetal bovine serum (164210; Pernos Life Sciences) and 1% antibiotic mixture (PB180120; Pernos Life Sciences). Cells were grown in a humidified environment at 37 °C and 5% CO_2_. Hypoxia was induced at an oxygen concentration of 1%, and the cells were grown for 72 h at 37 °C and 5% CO_2_ in a humidified environment.

### Lentiviral transfection

SUCLG2 lentivirus was purchased from and synthesised by HANBIO (www.hanbio.net, Shanghai, China). The oligonucleotide-containing shRNA sequences were: shRNA-NC: 5′-TTCTCCGAACGTGTCACGTAA-3′, shRNA-1: 5′-siRNA1 sequence: CAGGCAGTTCAATTAACCTCCAGAA-3′, shRNA-2: 5′-CAATAGTGGTTTGAAAGGAGGTGTT-3′, shRNA-3: 5′-CGAAGCTGTATAATCTCTTCCTGAA-3′, and LMNA shRNA: 5′-CTGACTTCCAGAAGAACA-3′. Subsequently, these shRNA oligonucleotides were inserted into the recombinant vector pgmlv-hu6mcs-cmv-mscarlett-pgk-puro to form the shRNA for SUCLG2 lentiviral particles generated with PEI (BMS1003-A; Invitrogen, Carlsbad, CA). The cells were seeded in six-well plates at 5 × 10^5^ cells/well. After 24 h and 40–50% fusion, lentiviral infections were performed according to the manufacturer’s provided multiplicity of infection. Lentiviruses containing SUCLG2 shRNA (50 nM) and shRNA-NC (50 nM) were added as controls. After incubation for 72 h at 37 °C, cells were passaged and inoculated into new six-well plates. After lentiviral transfection and passaging for 36 h, stable cell lines were screened using puromycin (2 µg/mL, CM00466).

The DNA coding sequence of mature LMNA (amino acid 470) was cloned in tandem into CV702, resulting in an expression construct driven by a CMV enhancer and containing a 3FLAG tag and puromycin resistance gene. Next, a K470R mutation was introduced into the LMNA sequence using the Mut Express II Rapid Mutagenesis Kit V2 (Vazyme, Nanjing, China).

### siRNA transfection

The siRNA sequences of the target gene markers were synthesised by RiboBio. The cells were seeded in six-well plates at 5 × 10^5^ cells/well. After 24 h and 70–80% fusion, siRNA transfection was performed using RNAiMAX (13778030; Invitrogen) according to the manufacturer’s instructions. After incubation for 20 min, the culture medium in each well was replaced with a complete culture medium containing 10% foetal bovine serum, and incubation was repeated for 48 h. The siRNA sequences were: SUCLG2 siRNA1 forward: 5′-GGTACAATCTAGCGACAAA-3′; SUCLG2 siRNA2 forward: 5′-CAACGCAGAATTCCGACAA-3′. CMV enhancer-MCS-3FLAG-SV40-Puromycin, NM_170707(K470R).

### RT-qPCR

Different grades of glioma tissues and HA, U87, U251, and LN229 cell lines were collected using TRIzol reagent (Thermo Fisher Scientific, Waltham, MA), and RNA was extracted using a Total RNA Extraction Kit (Tiangen Biochemistry Technology, Beijing, China). cDNA was synthesised using the FastKing cDNA First Strand Synthesis Kit (Tiangen Biochemical Technology) according to the manufacturer’s instructions. cDNA was amplified via PCR using SYBR Green PCR Master Mix (Q331-02; Vazyme), and PCR amplification was performed on a CFX96 Touch Real-Time PCR Detection System (Bio-Rad, Hercules, CA) for RT-qPCR detection. The relative mRNA expression levels were visualised using the 2-ΔΔCT method. The primer sequences were: SUCLG2, F5′-ACTTCTTGGATCTTGGAGGTGGTG-3′ and R5′-TTGGCAATGATGGCACAGTTGAC-3′; GAPDH, F5′-CACCCACTCCTCCACCTTTGAC-3′ and R5′- GTCCACCACCCTGTTGCTGTAG-3′.

### ChIP assay and ChIP-qPCR

LN229 and U251 cells were harvested and fixed in PBS using 1% formaldehyde for 10 min. Subsequently, 1.25 mol/L glycine was added to halt the cross-linking process. After washing, the fixed cells were resuspended in a lysis buffer (1% SDS, 50 mmol/L Tris–HCl pH 8.1, 5 mmol/L EDTA, and protease inhibitor) for 1 h. To generate chromatin fragments of approximately 300 bp, the cells were sonicated and centrifuged for 10 min at 4 °C and 4000×*g*. The lysates were diluted in a buffer containing 1% Triton-X-100, 20 mmol/L Tris–HCl (pH 8.1), 150 mmol/L NaCl, 2 mmol/L EDTA, and protease inhibitors.

Rabbit anti-IgG (2 µg) or rabbit anti-H4K16la (2 µg) was introduced into the diluted chromatin and placed in constant rotation overnight at 4 °C. The following day, 30 µL of Dynabeads™ Protein G (Invitrogen) was added and incubated for 4 h at 4 °C. After washing, the input and pull-down chromatin complexes were unchained for 12 h at 65 °C. Subsequently, the DNA obtained from the pull-down was purified using the MinElute PCR purification kit (Qiagen, Hilden, Germany). ChIP-qPCR was performed on a QuantStudio 5 real-time PCR system using PerfectStart Green qPCR SuperMix (TransGen Biotech, Beijing, China). The primer sequences for ChIP-qPCR included: MBD6, F5′-TCAGAGGAGGACATGACCAAGC-3′ and R5′-AGGAGAAGAGTGTGAGCAGGTG-3′; GRAMD4, F5′-CCGACTGGTACTCCGTCTACAC-3′ and R5′-GGCGATGAGGTAATTGAGGGATAAC-3′; Best1, F5′-CCTGTTGGGCTGTGGATGAGATG-3′ and R5′-CCTCCTCGTCCTCCTGATTGG-3′.

### Western blot analysis

Total proteins were extracted from glioma cell lines and tissues using a radioimmunoprecipitation assay buffer containing protease inhibitors (Beyotime, Jiangsu, China). Protein concentration was determined using the bicinchoninic acid method (Thermo Fisher Scientific) after sufficient centrifugation and boiling denaturation. Proteins were separated via sodium dodecyl sulfate–polyacrylamide gel electrophoresis and transferred onto polyvinylidene difluoride membranes. The membranes were closed with 5% skimmed milk solution for 2 h at room temperature, incubated in appropriate primary antibodies overnight at 4 °C, and in a secondary antibody solution for 1 h the next day. Membranes were washed thrice with Tris-buffered saline containing Tween 20 and visualised using an enhanced chemiluminescence reagent. The following antibodies were used: SUCLG2 (A8976, 1:1 200, ABclonal, Wobum, MA), Bax (60267-1-Ig, 1:1000, Proteintech, Rosemont, IL), PCNA (HRP-60097, 1:1 000, Proteintech), Caspase-3 (68773-1-Ig, 1:1000, Proteintech), Bcl-2 (12789-1-AP, 1:1000, Proteintech), β-actin (11313-2-AP, 1:1 500, Proteintech), Cyclin D1 (60186-1-Ig, 1:1500, Proteintech), DLAT (ab172617, 1:1000, Abcam, Cambridge, UK), LMNA (ab172617, 1:1000, Abcam), L-Lac (PTM-1401RM, 1:1000, Jingjie Bio, Nanjing, China), D-Lac (PTM-1429RM, 1:1000, Jingjie Bio), HIF-1α (H1alpha67, 1:1000, Abcam), H4K16la (PTM-122, 1:1000, Jingjie Bio), H4 (PTM-1015RM, Jingjie Bio), Total oxidative phosphorylation Rodent Antibody Cocktail (ab110413, 1:1000, Abcam), BEST1 (1:1000, ab259836, Abcam); GRAMD4 (1:1000, 24299-1-AP9, Proteintech), MBD6 (1:1000, ab204403, Abcam); MFN1 (A21293, 1:1200, ABclonal); MFN2 (A19678, 1:1200, ABclonal); DR1 (A13298,1:1200, ABclonal); IL-6 (A26791, 1:1000, ABclonal); IL-8 (RP00052, 1:1000, ABclonal); anti-rabbit IgG (H + L) (ab205718, 1:5000, Abcam), and anti-mouse IgG (H + L) (ab205719, 1:5000, Proteintech).

### CCK-8 assay

To determine the proliferative response of LN229-NC, LN229-3, U251-NC, and U251-3 cells, 2000 treated GBM cells/well were inoculated into 96-well plates and cultured for 24, 48, 72, and 96 h. L-Lac (10 nM) was added to the LN229 and U251 cell blank and knockdown groups. After incubation for 24 h, the cells were incubated with 10 µL CCK-8 solution (C0037, Biyuntian, China) for 2 h at 37 °C. The absorbance was measured at 450 nm using an enzyme marker (Tecan Trading AG, Männedorf, Switzerland).

### Subcutaneous xenografts and drug treatment

LN229 cells (5 × 10^7^) were suspended in 100 mL of high-glucose Dulbecco’s modified Eagle’s medium and injected subcutaneously into the backs of BALB/c nude mice (Lanzhou Veterinary Medical Research Institute). In the L-lac experiment, BALB/c nude mice were randomly divided into four groups (*n* = 4/group): (1) LN229NC control; (2) LN229NC treatment; (3) LN229-3 control; and (4) LN229-3 treatment. Nude mice with subcutaneous tumours were randomly divided to receive daily intraperitoneal injections of sodium lactate (120 mg/kg) or an equivalent amount of saline. Tumour diameters were measured with callipers, and the measured tumour volume was estimated using the formula: 0.5 × length × width^2^. Nude mice were sacrificed when tumour growth in the control group peaked, per Institutional Animal Care and Use Committee guidelines. Nude mice were euthanised after the treatment. Subsequently, the tumours were excised, weighed, photographed, and immediately fixed for subsequent IHC. All nude mice were drug- and test-primed before the experiment.

Female BALB/c nude mice (4-week-old) were provided by the Lanzhou Veterinary Research Institute, and eight 4-week-old female SUCLG2 mice were used as tumour models. For the intracranial tumour model, mice were anesthetised with isoflurane (RWD, Shenzhen, China). Following surgical anaesthesia, the mice were placed in a digital stereotaxic device (RWD). After complete exposure of the skull at the coordinates of the injection site (+0.7 mm anterior, −2.0 mm lateral distance from bregma), LN229/LN229sh-3 cells (2 × 10^5^, 5 µL) were slowly injected at a depth of 3 mm and allowed to stand for 10 min. The mice were sacrificed on Day 19 after injection, and the brain tissue was extracted. All magnetic resonance imaging experiments on intracranial tumour formation were performed on a 30-cm aperture horizontal 9.4 T system (UMR9.4T, Union Life Sciences Instruments, Wuhan, China). Nude mice were intracranially implanted with LN229-NC or LN229-3 cells, anesthetised on Day 17, and placed in a fixed system for imaging. The following sequence parameters were used: repetition time = 3000 ms; echo time = 49.28 ms; flip angle = 180°; matrix size = 208 × 208; field of view = 20 × 20 mm.

### Enzyme-linked immunosorbent assay

The cellular lactate content was determined using a human enzyme-linked immunosorbent assay kit (E-BC-K044-M, Shanghai Enzyme-linked Biotechnology Co., Ltd., Shanghai, China), according to the manufacturer’s instructions. Briefly, 100 µL of cell lysates from the indicated LN229NC or LN229 knockdown group were collected and incubated for 60 min at 37 °C. The lactate detection antibody, streptavidin–horseradish peroxidase, and tetramethylbenzidine were added, and the colour intensity was measured at 450 nm using a spectrophotometer.

### IHC and immunofluorescence

IHC was performed through 4% paraformaldehyde fixation, paraffin embedding, and sectioning. A primary antibody against SUCLG2 (A8976, 1:100, ABclonal) was used. After incubation with primary and secondary antibodies, horseradish peroxidase-labelled streptavidin solution was added to the samples and incubated for 15 min. Staining with 3,3’-Diaminobenzidine showed immunocomplexes, and haematoxylin counterstained cell nuclei. Images were obtained using a microscope (Ax10 Axio; Zeiss, Oberkochen, Germany).

Cells were incubated with an L-Lac antibody (PTM-1401RM, 1:1000, Jingjie Bio, China) overnight at 4 °C, followed by incubation with a secondary antibody (1-AP, Proteintech) for 1 h. The cell nuclei were stained with 4,6-diamidino-2-phenylindole (Solarbio, Beijing, China C0065) for 10 min. Images were acquired using a ZeissLSM880 confocal system and analysed with ImageJ. A fluorescein (FITC) Tunel Cell Apoptosis Detection Kit (G1501-50T, Servicebio, Wuhan, China) was used to evaluate apoptosis according to the manufacturer’s instructions. Images were obtained using a fluorescence microscope (Ax10 Axio, Zeiss) and analysed with ImageJ software.

### Flow cytometry

The apoptosis rate was assessed through Annexin V-FITC and propidium iodide staining using an Annexin V-FITC Apoptosis Detection Kit (C1062S, Biyun Tian, China). Using the Cell Cycle Staining Kit (C1052, Biyuntian, China), cells were resuspended and fixed with 70% cryoethanol overnight at 4 °C for cell cycle analysis and stained with propidium iodide. Subsequently, flow cytometry was performed to detect the cell cycle distribution. All flow cytometric analyses were performed using CytoFLEX S (BD FACSAria Calibur, USA). All foetal calf serum data were analysed with FlowJoV10 or ModFitLT.

### EdU assay

The EdU assay was performed using the BeyoClick™ EdU-555 Cell Proliferation Assay Kit (Beyoncé Biotech, C0075S), following the manufacturer’s instructions, to analyse the proliferative capacity of each cell group after treatment with 10 nM L-Lac. Cells were seeded in six-well plates at 1 × 10^4^ cells/well and cultured in a complete medium containing 10% foetal bovine serum for 24 h. L-Lac was added after 24 h, and the cells were placed in a normoxic incubator for an additional 24 h of culturing. Next, the cells were incubated with L-Lac. Subsequently, the EdU labelling solution was added to the cells and incubated for 2 h. Images were captured using an Olympus IX71 microscope (Olympus Corp., Tokyo, Japan).

### Cell metabolism measurements

U251, LN229 control, and sh-3 stable cell lines were inoculated into 96XF-specific 96-well plates and incubated overnight or with 10 nM L-Lac. Subsequently, the medium was replaced with Seahorse XF Dulbecco’s modified Eagle’s medium in the absence of CO_2_ for 1 h. Changes in cellular metabolism were monitored in vitro using a Seahorse XFe96 Pro analyser (Agilent, China) according to the manufacturer’s instructions. To assess the real-time glycolysis rate (ECAR), 10 mM glucose, 1 mM oligomycin, and 80 mM 2-deoxyglucose were injected. Additionally, to assess mitochondrial respiration (OCR), 1 mM oligomycin, carbonyl cyanide 4-(trifluoromethoxy) phenylhydrazone (FCCP), and 2 mM antimycin A and fisetinone were sequentially introduced. ECAR and OCR were obtained by normalising to the total protein content and reported as pmol/min (OCR) and mpH/min (ECAR). All reagents were purchased from Agilent Technologies.

### Co-IP assay

Co-IP assay was performed using Co-IP magnetic beads, eluent (RM00022, ABclonal), and cell lysate (Beyotime) by adding 40 µL proteinA/G agarose (RM09008, ABclonal) with 2 µL antibody (A8976, SUCLG2, ABclonal: ab226198, LMNA, Abcam) and 1 µg IgG (AS126, ABclonal) at 4 °C. The coated beads were incubated and spun overnight, resuspended with 1 mL of cell lysate, and spun again overnight, according to the manufacturer’s instructions. Bead-bound proteins were released and analysed via western blot analysis.

### JC-1 mitochondrial membrane potential assay kit

Logarithmic growth-phase LN229-NC, LN229-3, U251-NC, and U251-3 cells were uniformly inoculated in six-well plates. When the cell density reached 30–45%, the solution was changed, and the cells were inoculated with SUCLG2 siRNA. After 72 h, the complete medium was aspirated. After removing the cell culture medium, 1 mL of JC-1 staining buffer was added, and the cells were washed twice. Next, 1 mL of cell culture medium and 1 mL of JC-1 staining working solution were mixed in the dark and incubated in a CO_2_ incubator for 30 min. The supernatant was aspirated, and the cells were washed twice with JC-1 staining buffer. Subsequently, 2 mL of JC-1 (1×) staining buffer was added to each well, and images were obtained using a fluorescence microscope. The fluorescence intensity was analysed using ImageJ.

### Electron microscopy

Cell precipitates were collected from log-phase U251-NC, 251-3, LN229-NC, and LN229-3 cells and fixed with 2.5% glutaraldehyde overnight at 4 °C. Subsequently, the cells were fixed with 2% osmium tetroxide for 1.5 h at room temperature. The cells were embedded and stained with uranyl acetate and lead citrate. Observations were made using a transmission electron microscope (JEM-1230; JEOL, Tokyo, Japan) at 60 kV.

### Molecular docking

The molecular docking of SUCLG2 main protease (PDB: 6WCV) with LNNA was conducted using HADDOCK. The protein structure was protonated at pH 7.4 with PDB2PQR, whereas the ligand was energy-minimised in Avogadro 1.2.0 using the MMFF94 force field. Docking grid dimensions (20 × 20 × 20 Å) were centred on the catalytic dyad (His41 and Cys145). From 10 generated poses, the top-scoring conformation (binding energy = −9.8 kcal/mol) was chosen for molecular docking simulations.

### MS-based label-free quantitative acetylation proteomics

Label-free quantitative acetylation proteomics was performed by Genechem (Shanghai, China). SDT buffer (4% SDS, 100 mM Tris–HCl, pH 7.6) was added to the LN229 cells transfected with shNC and shSUCLG2. The lysate was sonicated (this step could be skipped for protein solution) and boiled for 15 min. After centrifugation at 14,000×*g* for 15 min, the supernatant was quantified with a bicinchoninic acid protein assay kit (P0012, Beyotime). The sample was stored at −80 °C. Subsequently, 20 µg of proteins for each sample were mixed with 6× loading buffer and boiled for 5 min. The proteins were separated on a 12% gel via sodium dodecyl sulfate-polyacrylamide gel electrophoresis. Protein bands were visualised using Coomassie Blue R-250 staining. Next, 5 mg of proteins for each sample was reduced with 100 mM DTT for 5 min at 100 °C. Furthermore, the detergent, DTT, and other low-molecular-weight components were removed using a UA buffer (8 M Urea, 150 mM Tris–HCl, pH 8.5) through repeated ultrafiltration (Sartorius, 30 kDa). In addition, 100 mL of iodoacetamide (100 mM IAA in UA buffer) was added to block reduced cysteine residues, and the samples were incubated in the dark for 30 min. The filters were washed thrice with 100 μL of UA buffer and twice with 100 μL of 50 mM NH_4_HCO_3_ buffer. Finally, the protein suspensions were digested with 4 μg trypsin (Promega) in 40 μL of 50 mM NH_4_HCO_3_ buffer overnight at 37 °C, and the resulting peptides were collected as a filtrate. The peptide segment was desalted using a C18 column. The peptide content was estimated through ultraviolet spectral density at 280 nm using an extinction coefficient of 1.1 of 0.1% (g/l) solution that was calculated based on the frequency of tryptophan and tyrosine in vertebrate proteins. The peptide mixture was enriched for Kac peptides using Acetyl-Lysine Motif [Ac-K] Kit (Cell Signalling Technology, 13416S).

### Acetylation site analysis using tandem MS

As described above, eluted proteins were upsampled onto sodium dodecyl sulphate-polyacrylamide gel electrophoresis gels and stained with Coomassie Brilliant Blue. Samples of SUCLG2 bands were cut and subjected to tandem MS in a Q Exactive Mass Spectrometer (Thermo Fisher Scientific).

### Mass spectrometric data analysis

LN229 cells transfected with shNC and shSUCLG2 were analysed on a nanoElute (Bruker, Bremen, Germany) coupled to a timsTOF Pro (Bruker) equipped with a CaptiveSpray source. Peptides were separated on a 25 cm × 75 μm analytical column–1.6 μm C18 beads with a packed emitter tip (IonOpticks, Australia). The column temperature was maintained at 50 °C using an integrated column oven (Sonation GmbH, Germany). The column was equilibrated with four column volumes of 100% buffer A (99.9% MilliQ water, 0.1% formic acid) before sample loading (both steps were performed at 800 bar). Subsequently, the samples were separated at 300 nL/min using a linear gradient.

The timsTOF Pro (Bruker) was operated in PASEF mode with the following settings: mass range (100–1700*m*/*z*), 1/K0 (0.75–1.4 V s/cm^2^), ramp time (100 ms), lock duty cycle (100%), capillary voltage (1500 V), dry gas (3 L/min), and dry temperature (180 °C); PASEF settings: 10 MS/MS scans (total cycle time 1.16 s), charge range (0–5), active exclusion (0.5 min), scheduling target intensity (10,000), intensity threshold (2500), and Collision-Induced Dissociation collision energy (20–59 eV).

The MS data were analysed using MaxQuant version 1.6.17.0. MS data were searched against the database (determined by the project). An initial search was set at a precursor mass window of 6 ppm. The search followed an enzymatic cleavage rule of trypsin/P and allowed a maximum of two missed cleavage sites and a mass tolerance of 20 ppm for fragment ions. Carbamidomethylation of cysteines was defined as a fixed modification, whereas protein N-terminal acetylation and methionine oxidation were defined as variable modifications for database searching. The global false discovery rate cutoff for peptide and protein identification was set to 0.01. Protein abundance was calculated based on the normalised spectral protein intensity (LFQ intensity). Proteins with fold change >2 or <0.5 and *P*-value < 0.05 (Student’s *t*-test) were considered differentially expressed.

### Liquid chromatography (LC)–MS/MS

LC-MS/MS was conducted by Frasergen (Wuhan, China). Peptide samples from shNC and shSUCLG2 of LN229, as well as acetyl-CoA assay samples, were loaded onto a homemade packed capillary C18 column (25 cm length, 75 mm ID, particle size 1.9 µm, Dr. Maisch GmbH, Ammerbuch, Germany). LC–MS/MS was performed using an EASY-nLC 1200 UHPLC system (Thermo Fisher Scientific) coupled with a Q Exactive HF-X mass spectrometer (Thermo Fisher Scientific). The mobile phase A consisted of 0.1% formic acid (v/v) in water, whereas mobile phase B comprised 0.1% formic acid (v/v) in 80% acetonitrile. Peptides and acetyl-CoA were separated using a linear gradient of 10–90% mobile phase B (in mobile phase A), with durations of 60 and 15 min, respectively. Full-scan mass spectra were acquired in the *m*/*z* range using positive ion mode with an Orbitrap mass analyser, achieving a mass resolution of 60,000. MS/MS fragmentation was conducted in data-dependent acquisition mode, wherein the 20 most intense ions were selected for MS/MS at a resolution of 15,000 using the HCD collision mode.

### CUT&Tag

CUT&Tag was performed by Frasergen. LN229 control and experimental group cells were collected and analysed using a Hyperactive Universal CUT&Tag Analysis Kit (Illumina, Vazyme Biotechnology, TD903-01). Briefly, cells were collected and resuspended in 100 μL of rinse buffer. The cell suspension was incubated with activated Concanavalin A beads for 10 min at room temperature. The supernatant was discarded after instantaneous centrifugation, and the pellet was resuspended with 50 μL of a pre-cooled antibody buffer containing 1 μg of anti-H4Lys16 antibody (PTM-1015RM, Jingjie Bio). The cell suspension was incubated overnight at 4 °C. After discarding the supernatant, the precipitate was incubated for 60 min at room temperature with rotation in 50 μL of Dig-wash buffer containing a secondary antibody. Furthermore, the supernatant was removed, and the precipitate was incubated in 100 μL Dig-300 buffer containing 2 μl pA/G-Tnp for 60 min at room temperature. We aspirated the supernatant and incubated the precipitate in 50 μL Dig-300 buffer containing 10 μL 5× TTBL for 60 min at 37 °C to fragment the chromatin. Chromatin fragments were incubated with 5 μL of proteinase K, 100 μL of buffer L/B, and 20 μL of DNA extraction beads for 10 min at 55 °C. The supernatant was discarded, and the beads were washed twice with Buffers WA and WB. The supernatant was discarded, and the DNA was eluted with 22 μL of sterilised ultrapure water. Next, the library was amplified and sequenced on the Illumina Novaseq platform at Fraser Genetic Information Co., Ltd (Wuhan, China), and 150 bp paired-end reads were generated.

The CUT&Tag data analysis for H4Lys16 was performed as previously described. Raw fastq reads were first cleaned using fastp (version 0.20.0). The clean reads were compared with the reference genome using BWA (v0.7.12) memory. Only the uniquely mapped (MAPQ 13) and de-duplicated reads were used for further analysis. Peak calling was performed using MACS2 software (version 2.1.0), with peaks filtered at a *q*-value threshold of 0.05. Peak positions around transcription start sites were used to predict protein–gene interaction sites. Terminal next-generation sequencing was performed by Frasergen. Subsequent bioinformatics analyses aligned sequencing reads to the mouse mm10 genome and annotated the genomic peak regions. KEGG pathways of peak-associated genes were statistically enriched using KOBAS software. Integrated Genome Viewer was used to visualise peak distributions in protein–DNA binding-associated genomic regions.

### Statistical analysis

GraphPad Prism 9.4.1 (GraphPad Software Inc., CA, USA) was used for statistical analysis. All results from at least three independent experiments were reported as mean ± standard deviation. Comparisons between groups were performed using the two-tailed *t*-test, analysis of variance, or chi-squared test. Correlation analysis was performed using the Pearson correlation test. Univariate and multivariate Cox proportional risk regression models were used to identify survival-associated factors. Survival analyses were performed using Kaplan–Meier plots and log-rank tests. *P*-values < 0.05 indicated statistically significant differences.

## Supplementary information

Supplementary figure legend

Figure S1

Figure S2

Figure S3

Figure S4

Western blot images

Specimen number S1

Subcellular_Location

label-free quantitative acetylation proteomics

IP-MS protein list

Acetylation site analysis

## Data Availability

The datasets used and analysed during the current study are available from the corresponding author upon reasonable request.
