# Supplementary material for: Knockdown of SUCLG2 inhibits glioblastoma proliferation and promotes apoptosis through LMNA acetylation and the mediation of H4K16la lactylation
Source: Cell Death Discov. 2025 Nov 17;11:534. doi: 10.1038/s41420-025-02856-4 (PMC12623996; doi:10.1038/s41420-025-02856-4)
Supplement: Supplementary file 1 — Supplementary figure legend [file 41420_2025_2856_MOESM1_ESM.docx]

**Supplementary figure legends**

**Supplementary Figure 1.**

(S1A) Analysis of SUCLG2 expression in 163 cases of GBM compared with 207 normal brain tissues using the GEPIA database. The boxplot is presented. (S1B) The S1B GEPIA database was used to analyse SUCLG2 expression in most tumours.

**Supplementary Figure 2.**

(S2A) Magnetic resonance imaging of LN229 after intracranial *in situ* tumour implantation of shNC or shRNA to compare the tumour size. (S2B) Haematoxylin and eosin staining of tumour cross sections obtained after the intracranial tumour implantation of shNC or shRNA from LN229. (S2C) Comparison of survival time after the intracranial implantation of tumours with LN229 shNC vs LN229 shRNA. (S2D) Comparison of Ki-67 and SUCLG2 expression through the cross-section staining of LN229 shNC or shRNA. Immunohistochemical staining represents the figure. (S2E) Statistical graph of ATP5A, ND1, and SDHB protein expression in LN229 and U251 cells treated with shNC and shRNA. (S2F) Statistical graph of MFN1 protein expression in LN229 and U251 cells in shNC and shRNA. * *P*<0.05, ***P*<0.01, ****P*<0.001.

**Supplementary Figure 3.**

(S3A) GO analysis of upregulated proteins based on 4D acetylated proteomics sequencing. (S3B) Intracellular immunofluorescence co-localisation to determine the co-localisation of SUCLG2 and LMNA proteins in U251 and LN229 cells. (S3C) Cells were treated with acetylation inhibitors. Exogenous IPs of SUCLG2-HA, shLMNA, rLMNA-Flag, and rLMNA K470ac were performed, followed by western blotting. Cells were treated with TSA (10 mM) and NAM (10 mM) for 16 h. (S3D) Statistical graph of L-Lac, LDHA, and LDHB protein expression in LN229 and U251 cells with shNC and shRNA. (S3E) Statistical graph of PCNA, D1, Caspase3, Bax and Bcl-2 protein expression in LN229 and U251 cells with 10 nM L-lac added to shNC and shRNA. (S3F) Statistical graphs of ATP5A, ND1, and SDHB protein expression in LN229 and U251 cells with 10 nM L-lac added to shNC and shRNA. * *P*<0.05, ***P*<0.01, ****P*<0.001.

**Supplementary Figure 4.**

(S4A) In U251 and LN229 cells containing shNC and shRNA, 10 nM L-Lac was added for 6 hours, followed by JC-1 staining, and mitochondrial membrane potential was observed using a fluorescence microscope. (S4B) After adding 10 nM L-Lac to LN229 and U251 cells in shNC and shRNA, the expression of MFN1, MFN2, and DRP1 was analysed by Western blotting. (S4C) Statistical graph of L-lac and H4K16la expression in LN229 and U251 cells in shNC and shRNA. (S4D) Statistical graphs of BEST1, GRAMD4, and MBD6 expression in LN229 and U251 cells in shNC and shRNA. (S4E) Statistical graph of IL-6 and IL-8 expression in LN229 and U251 cells in shNC and shRNA. * *P*<0.05, ***P*<0.01, ****P*<0.001.
