# Supplementary figures and images for: Knockdown of SUCLG2 inhibits glioblastoma proliferation and promotes apoptosis through LMNA acetylation and the mediation of H4K16la lactylation
Source: Cell Death Discov. 2025 Nov 17;11:534. doi: 10.1038/s41420-025-02856-4 (PMC12623996; doi:10.1038/s41420-025-02856-4)

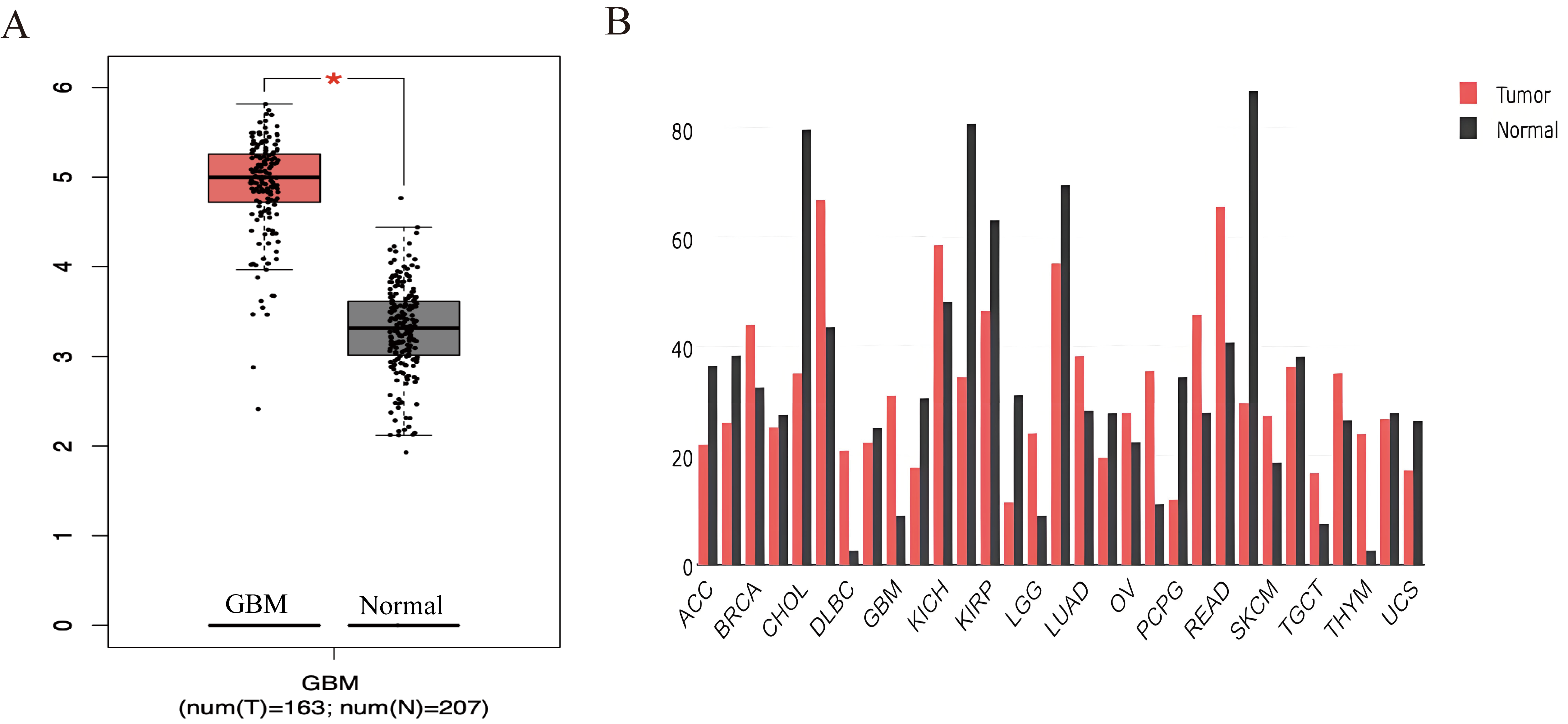

Supplement: Supplementary file 2 — Figure S1 [file 41420_2025_2856_MOESM2_ESM.tif]

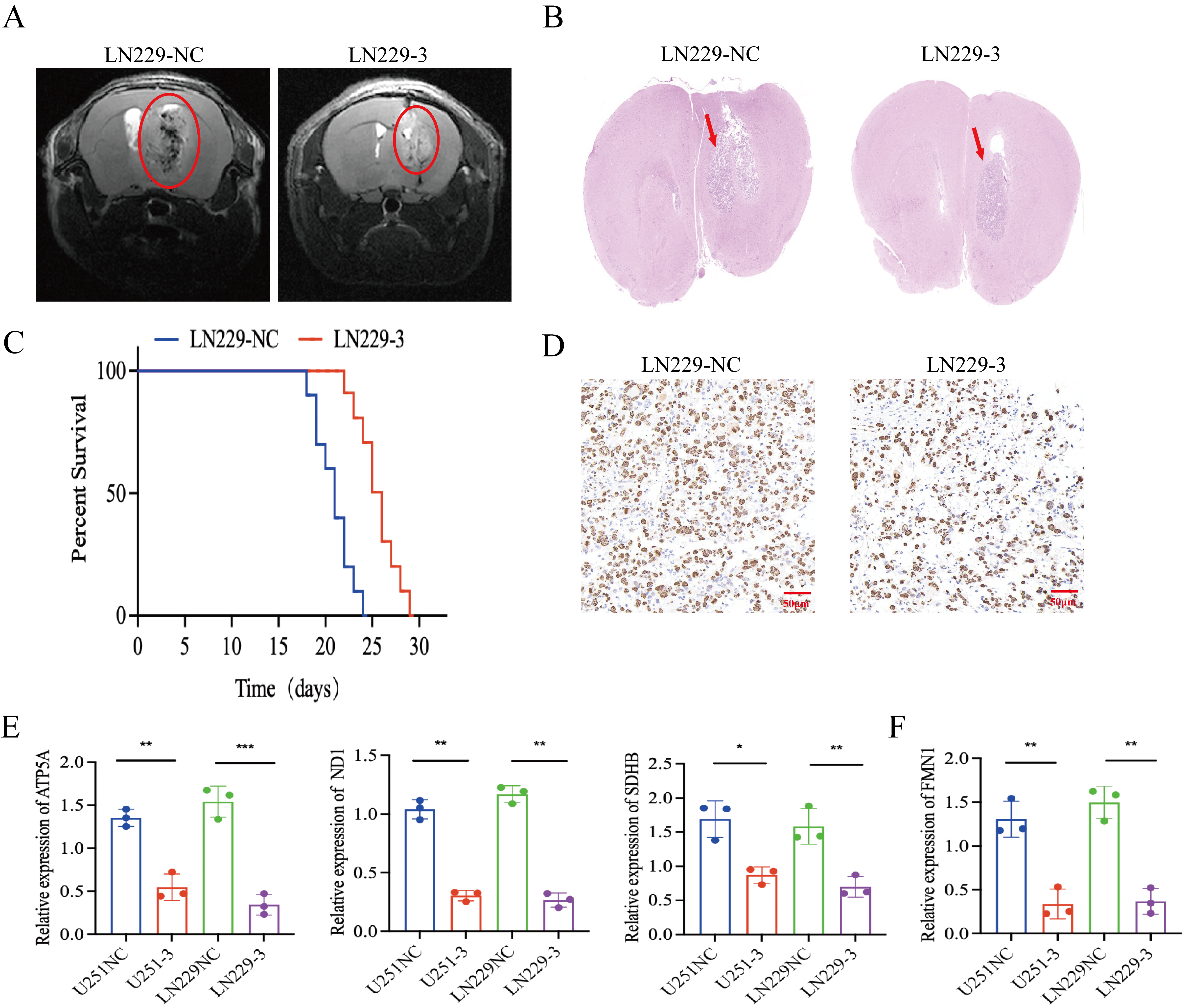

Supplement: Supplementary file 3 — Figure S2 [file 41420_2025_2856_MOESM3_ESM.tif]

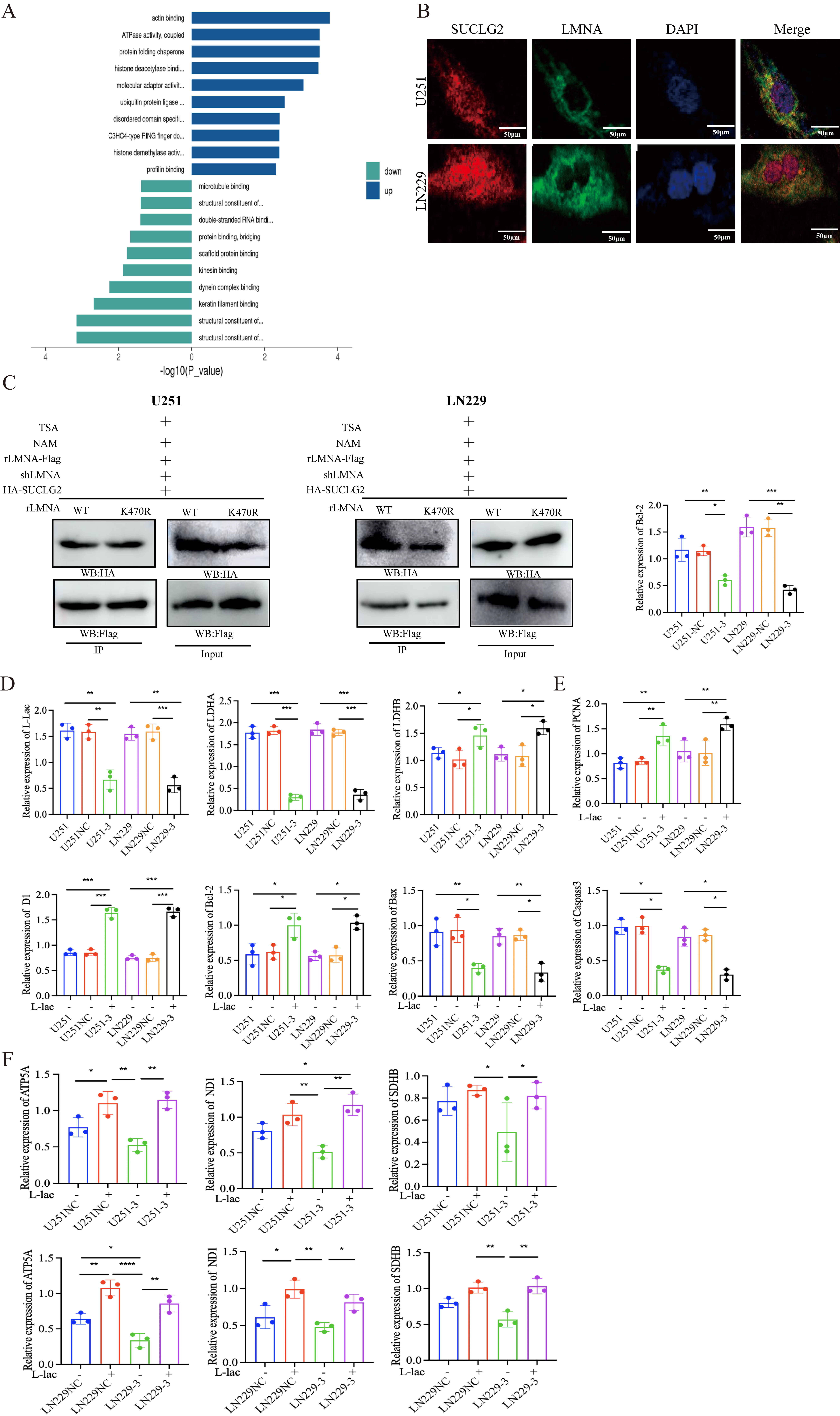

Supplement: Supplementary file 4 — Figure S3 [file 41420_2025_2856_MOESM4_ESM.tif]

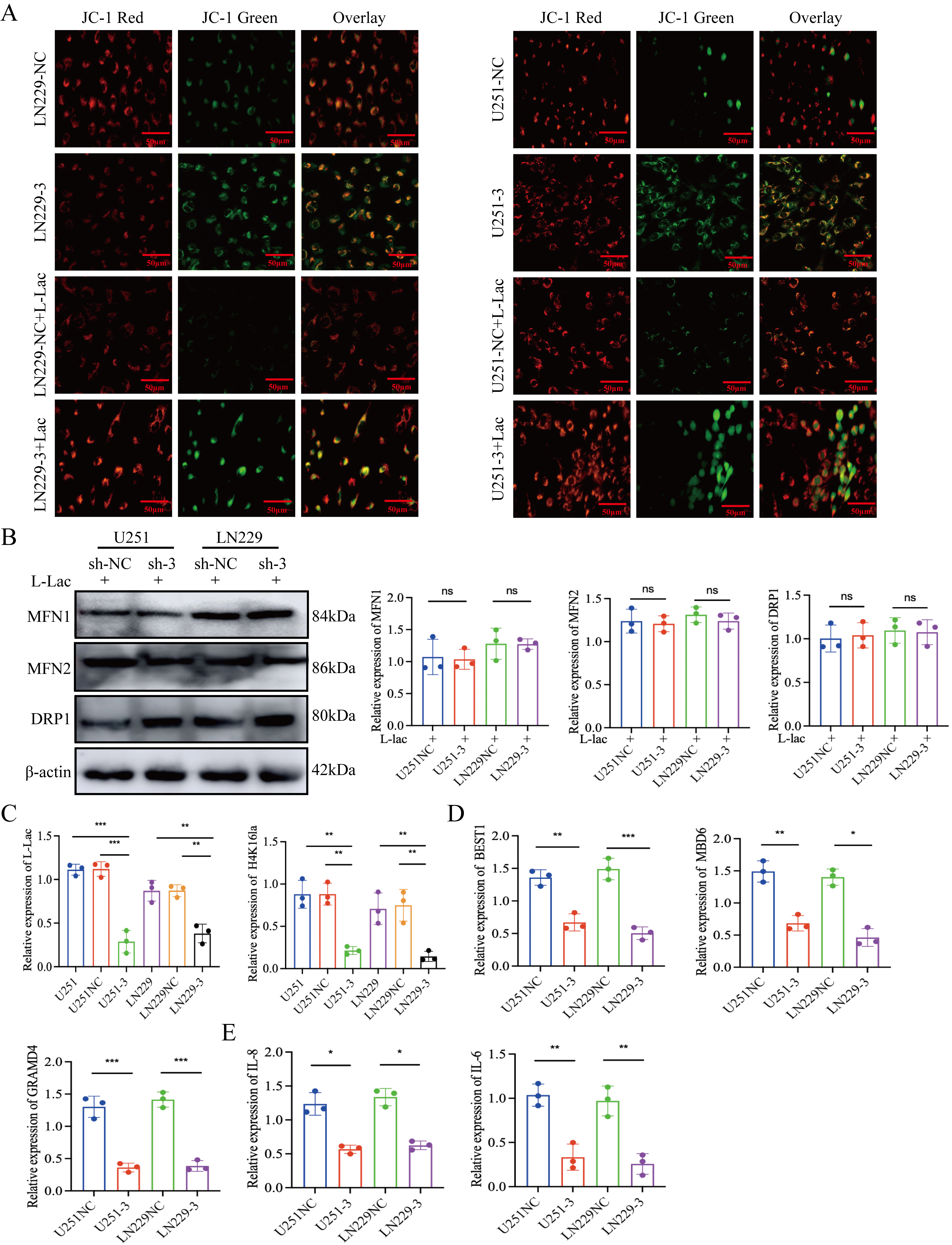

Supplement: Supplementary file 5 — Figure S4 [file 41420_2025_2856_MOESM5_ESM.tif]
