# Supplementary material for: Knockdown of SUCLG2 inhibits glioblastoma proliferation and promotes apoptosis through LMNA acetylation and the mediation of H4K16la lactylation
Source: Cell Death Discov. 2025 Nov 17;11:534. doi: 10.1038/s41420-025-02856-4 (PMC12623996; doi:10.1038/s41420-025-02856-4)
Supplement: Supplementary file 6 — Western blot images [file 41420_2025_2856_MOESM6_ESM.docx]

Raw strip data:

Organize WB strips:

SUCLG2:

Normal II III IV


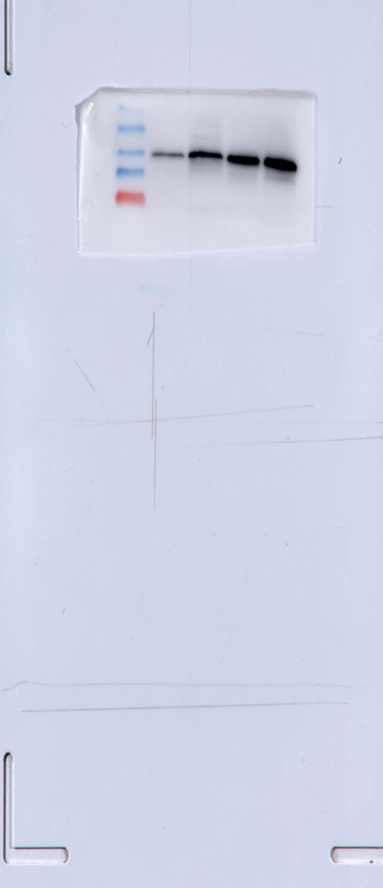

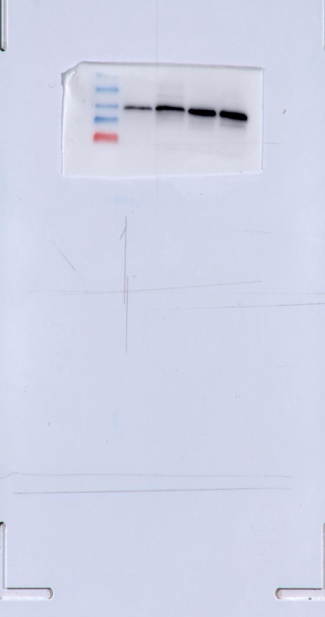

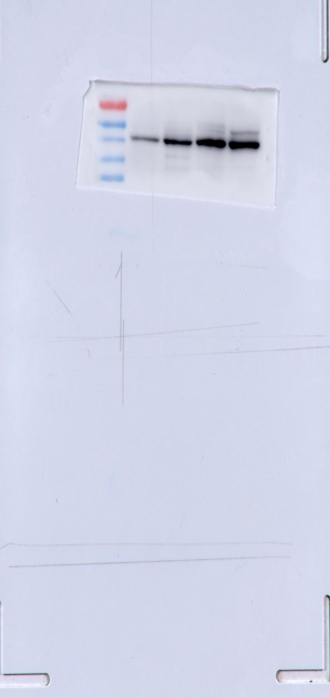


ACTIN: Normal II III IV


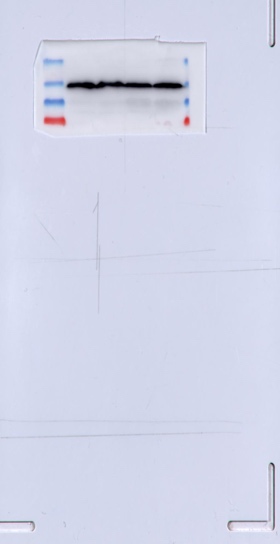

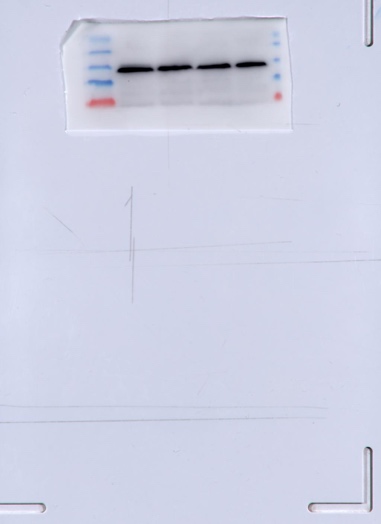

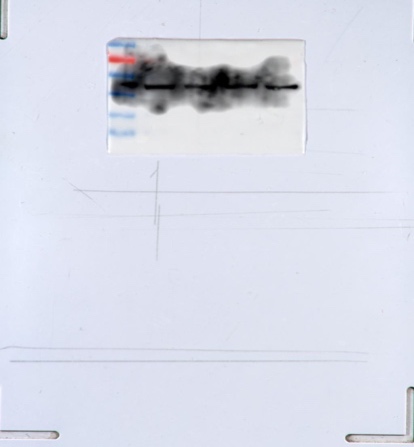


Cells：HA. U87. U251. LN229. U118


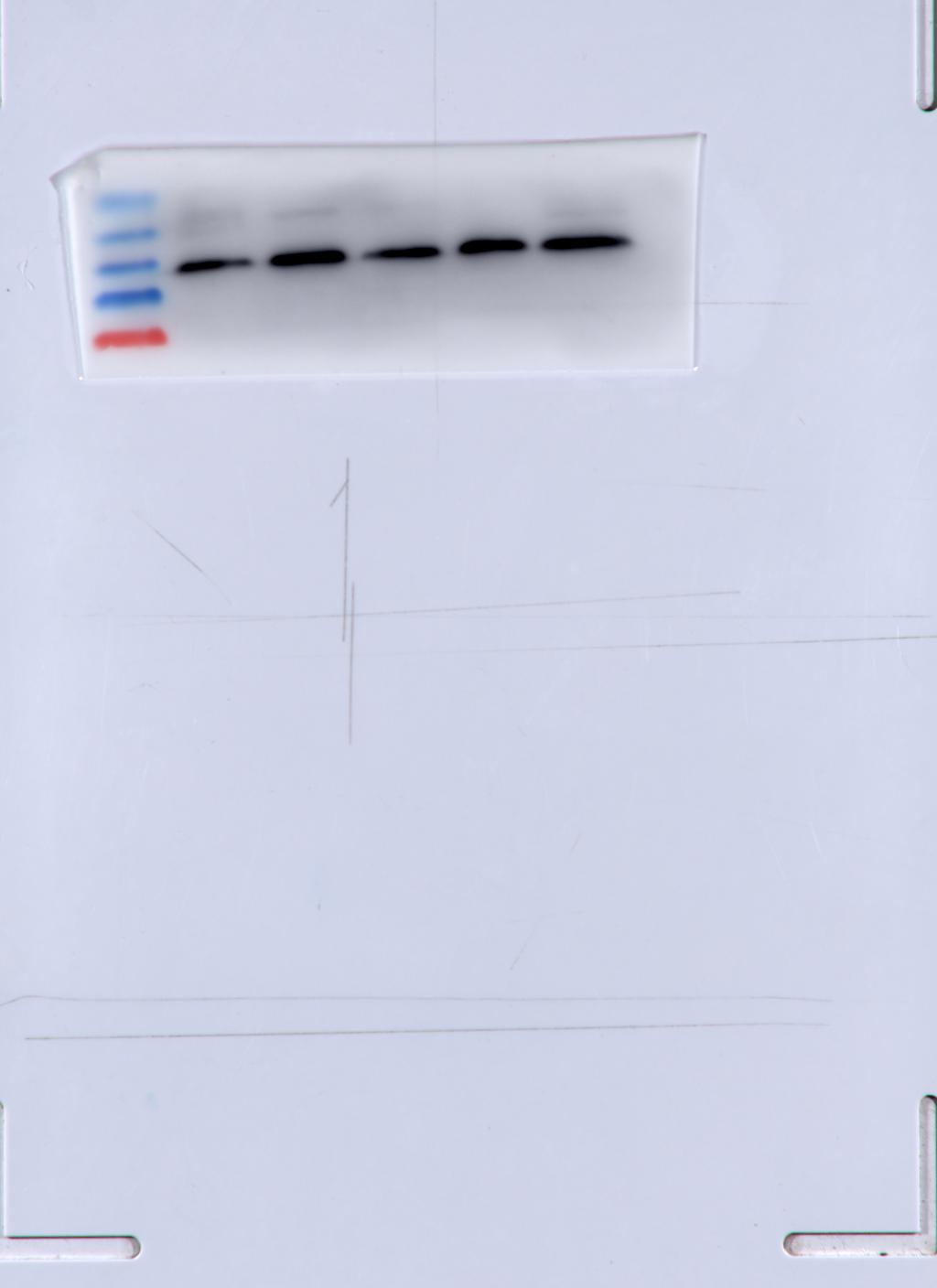

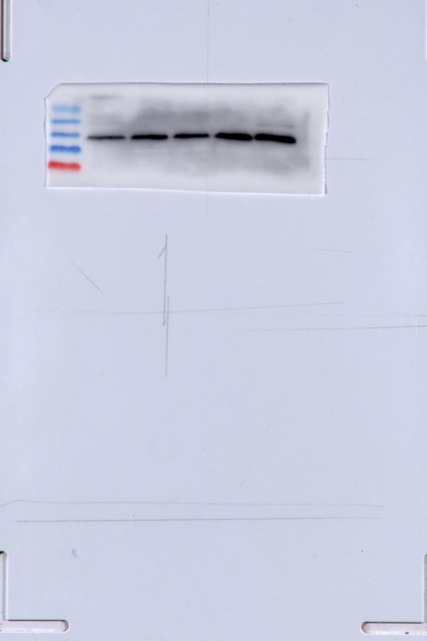

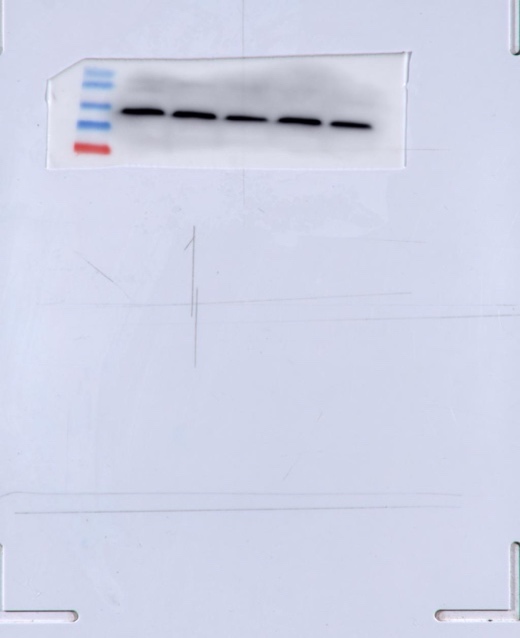


Actin：HA. U87. U251. LN229. U118


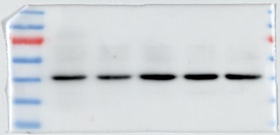

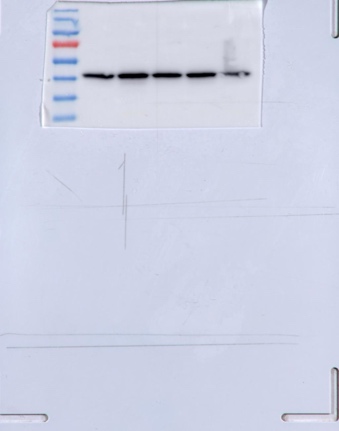

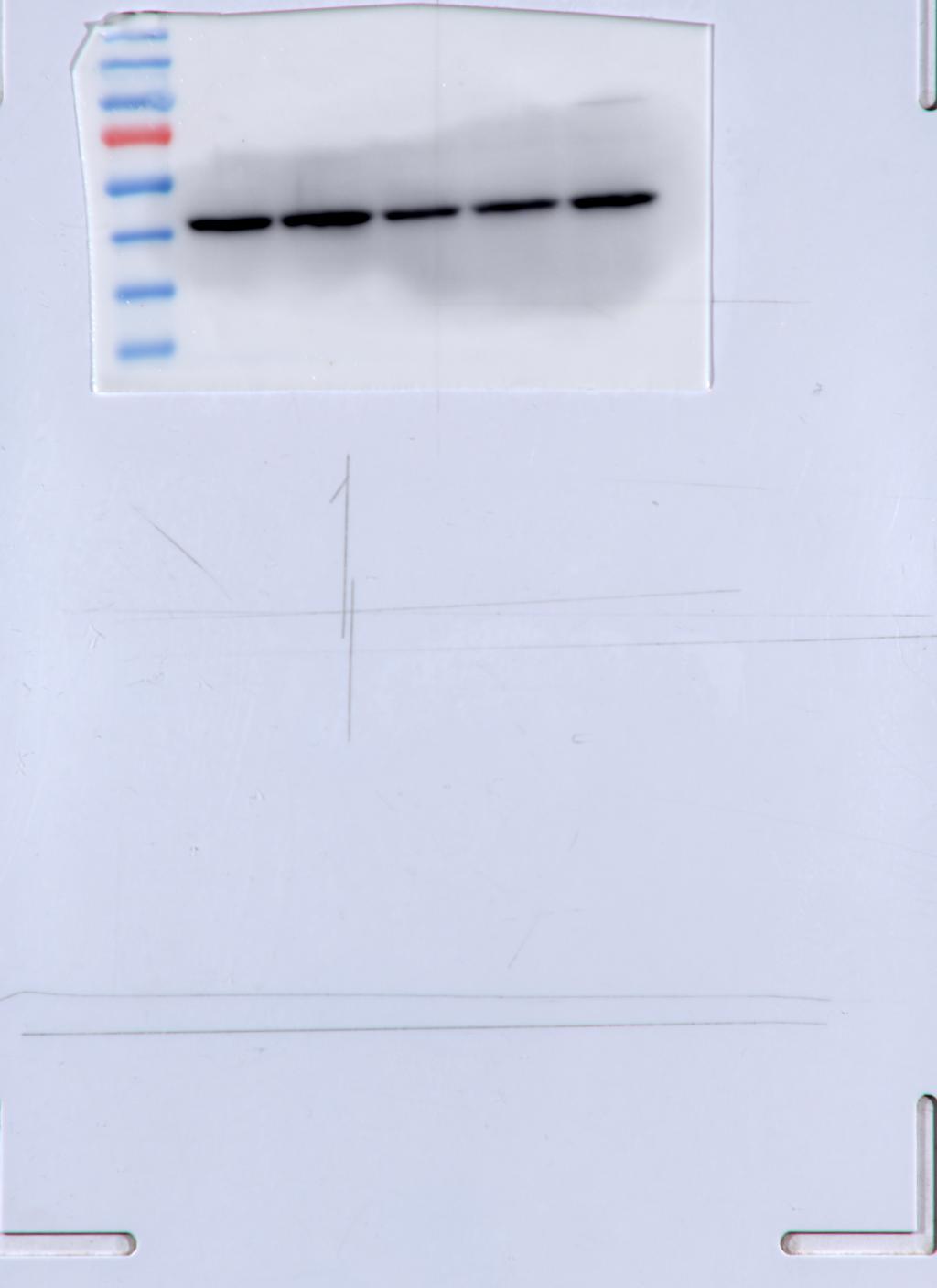


shSUCLG2：

U251： U251NC U251sh-1 sh-2 sh-3；


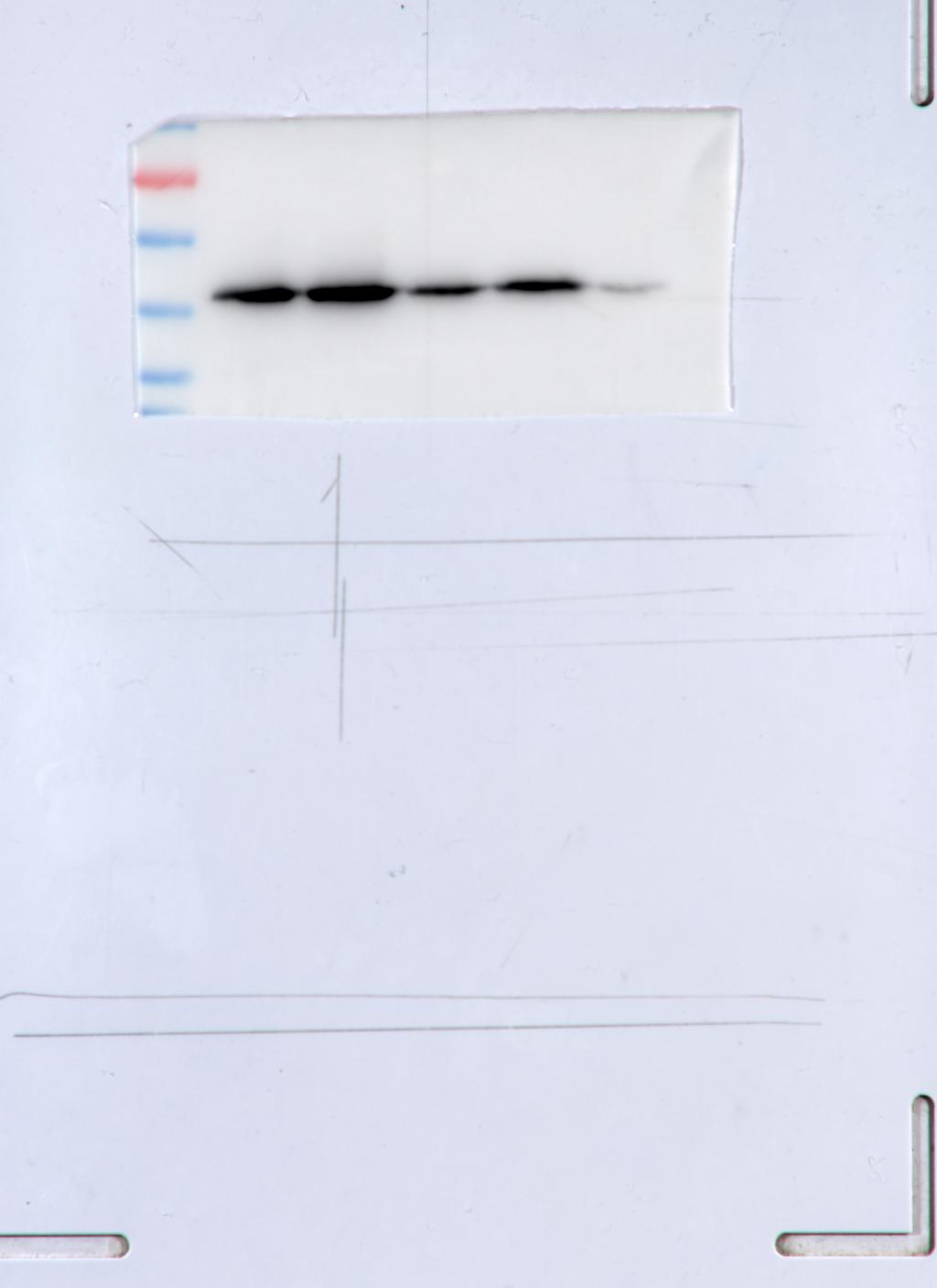

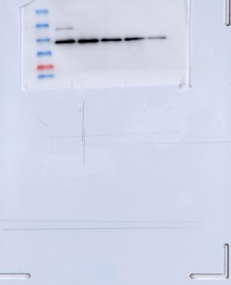

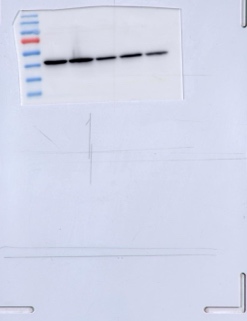


Actin：


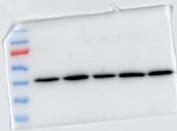

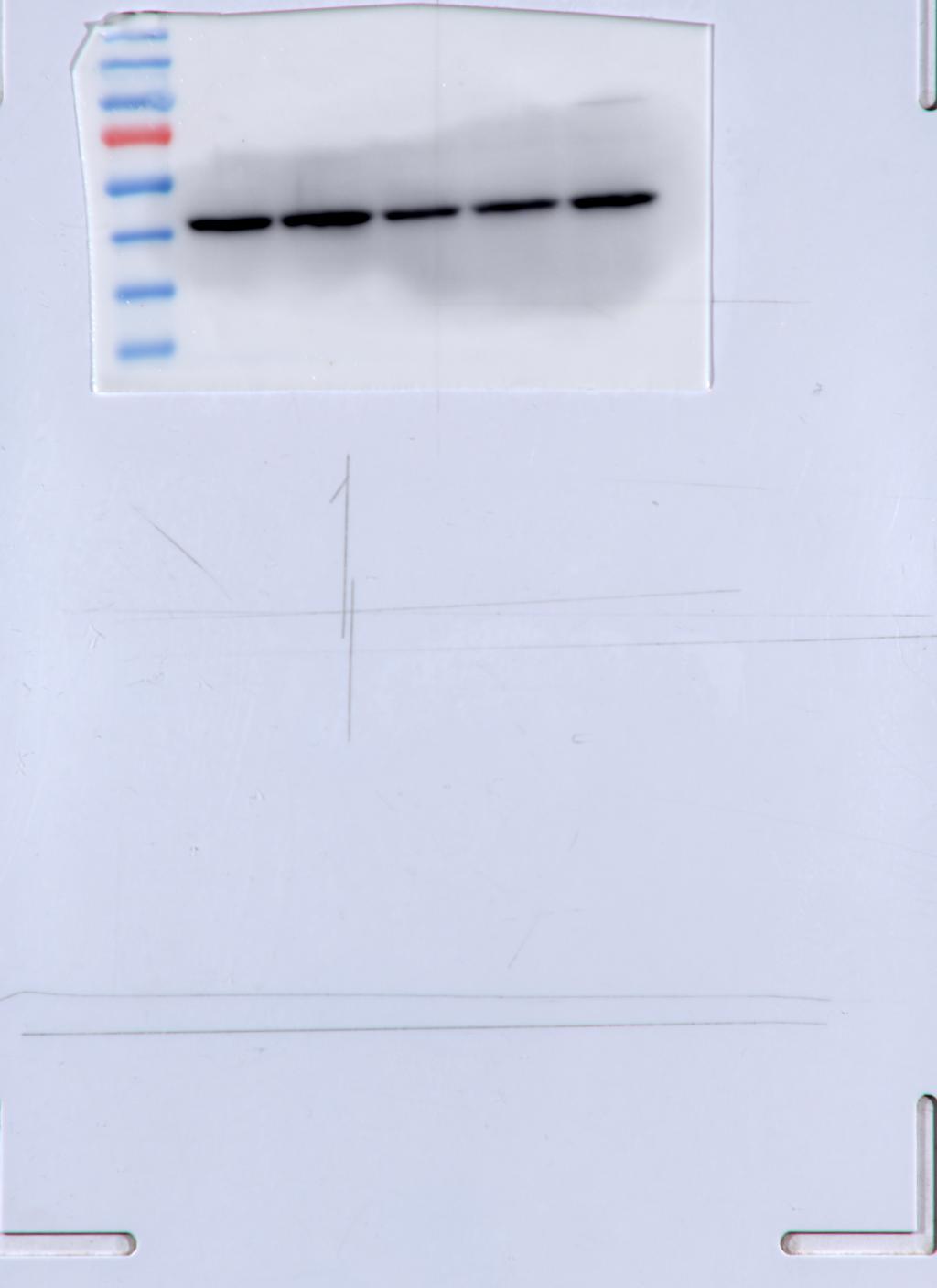

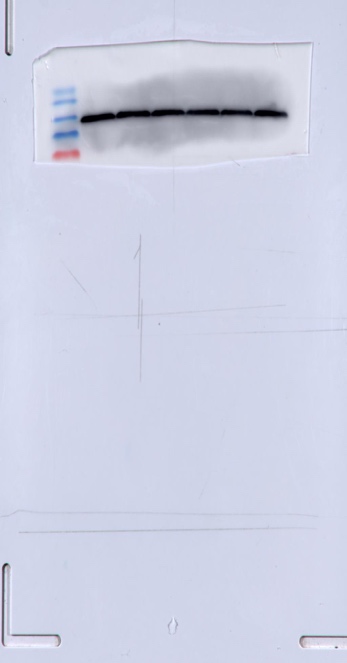


LN229： LN229NC 251sh-1 sh-2 sh-3


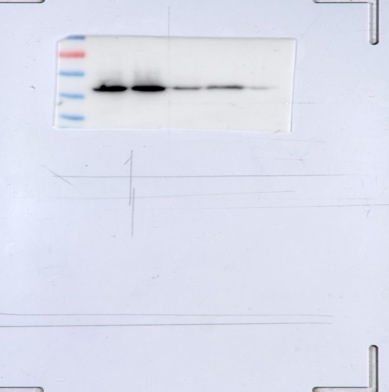

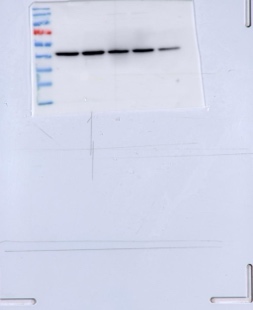

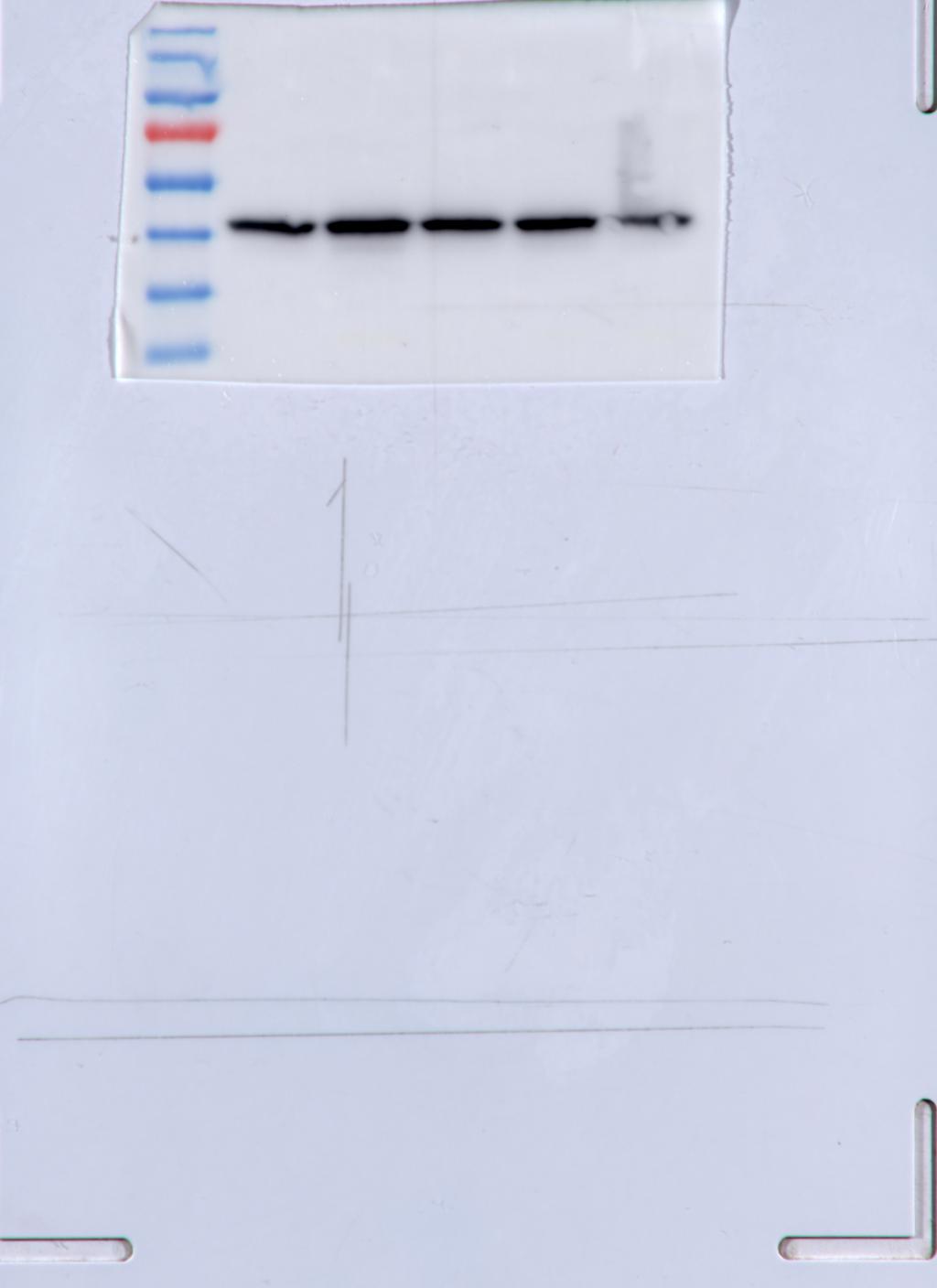


Actin


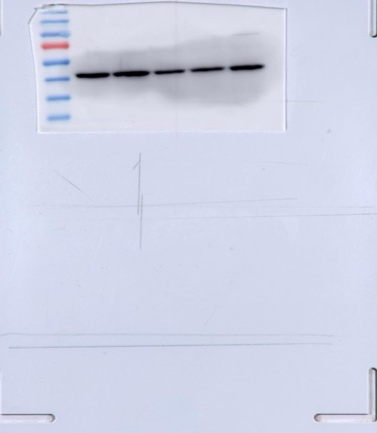

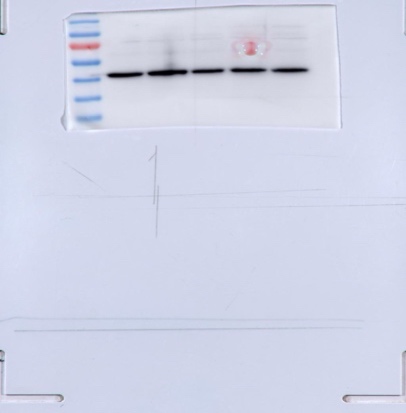

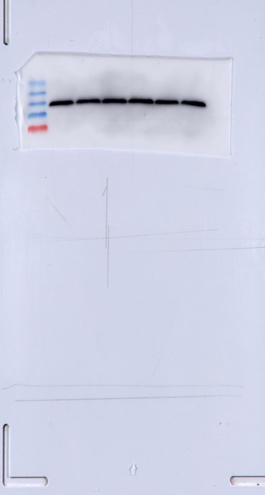


Caspase3:

251 251NC sh-3 LN229 LN229NC sh-3


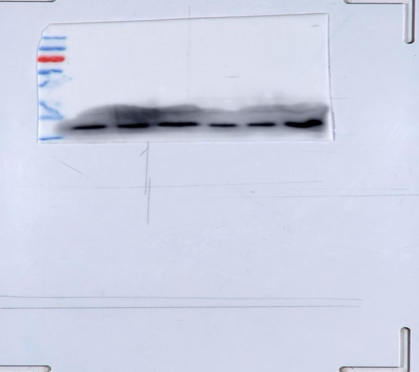

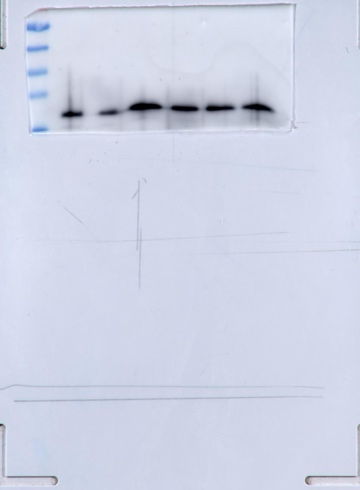

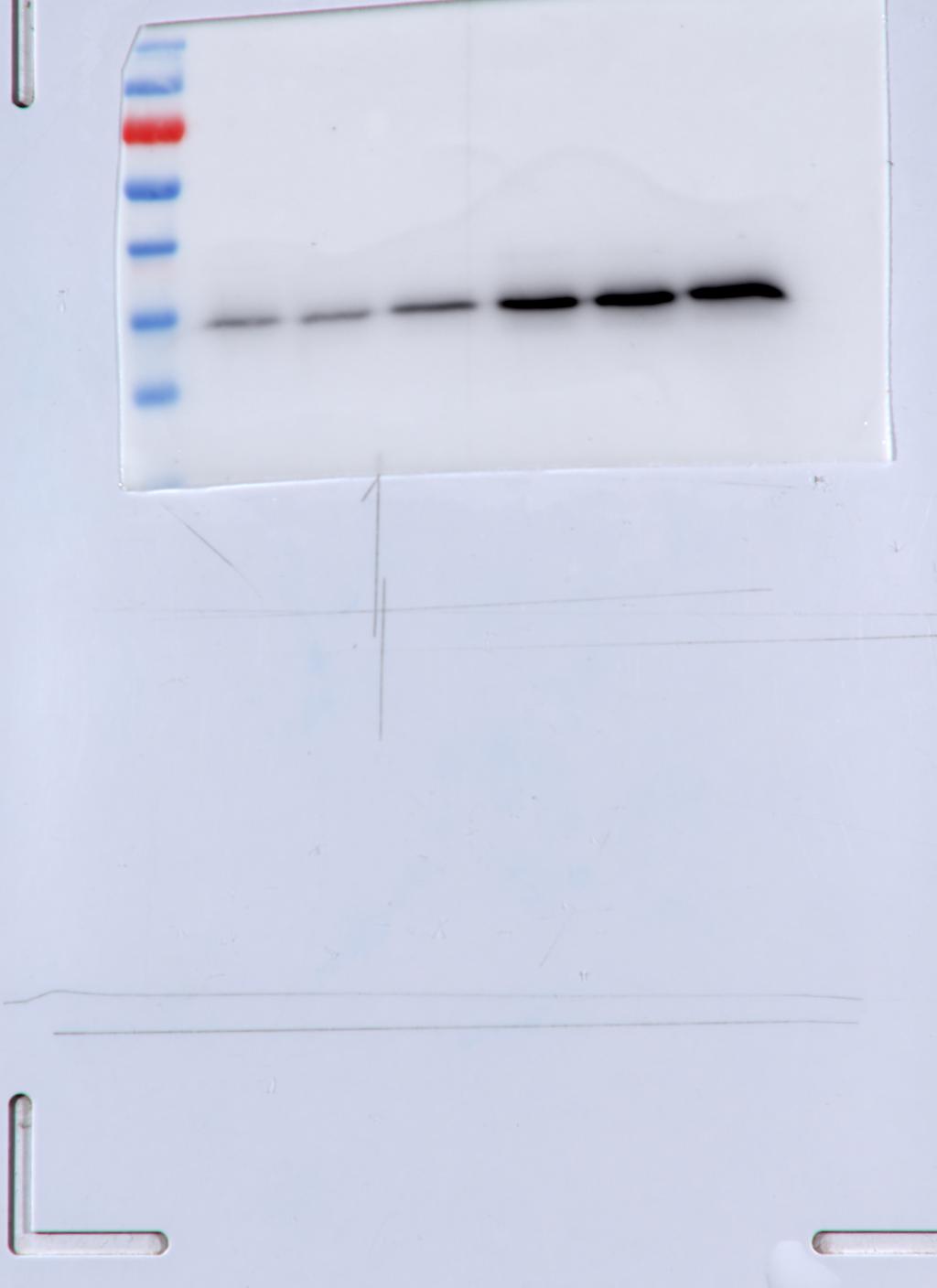


Bcl2 U251 251NC sh-3 LN229 LN229NC sh-3


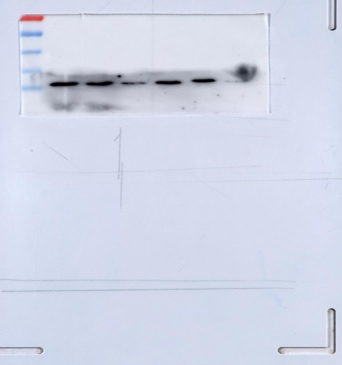

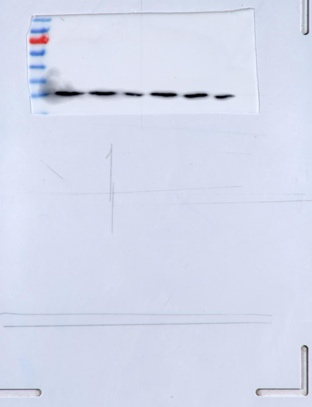

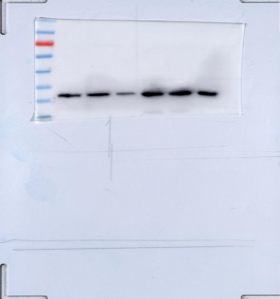


Bax：251 251NC sh-3 LN229 LN229NC sh-3


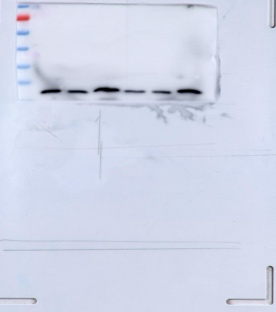

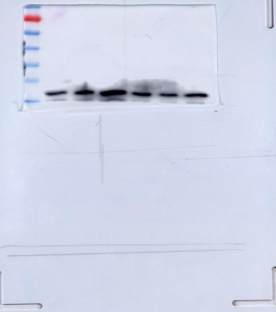

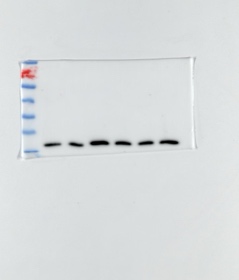


D1：251 251NC sh-3 LN229 LN229NC sh-3


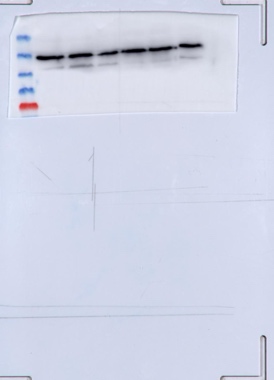

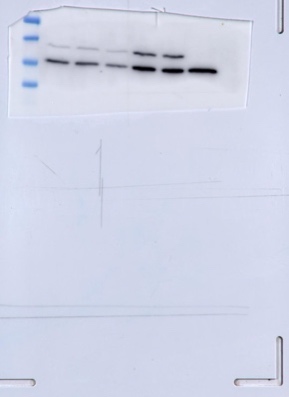

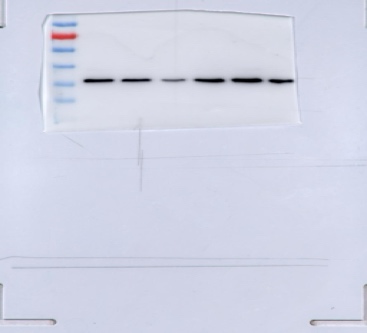


PCNA

U251 U251NC sh-3 LN229 LN229NC sh-3


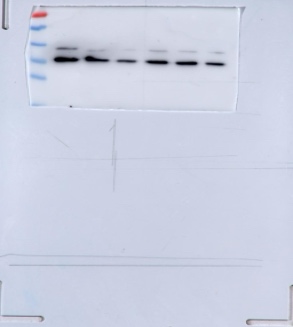

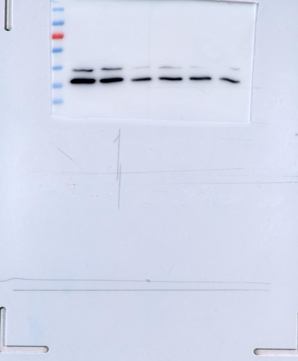

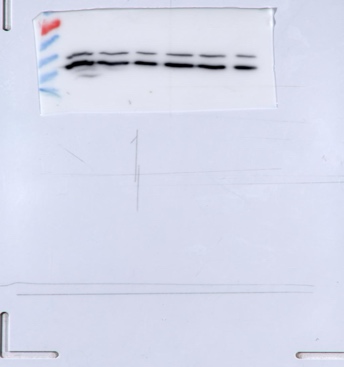


ACTIN：U251 U251NC sh-3 LN229 LN229NC sh-3
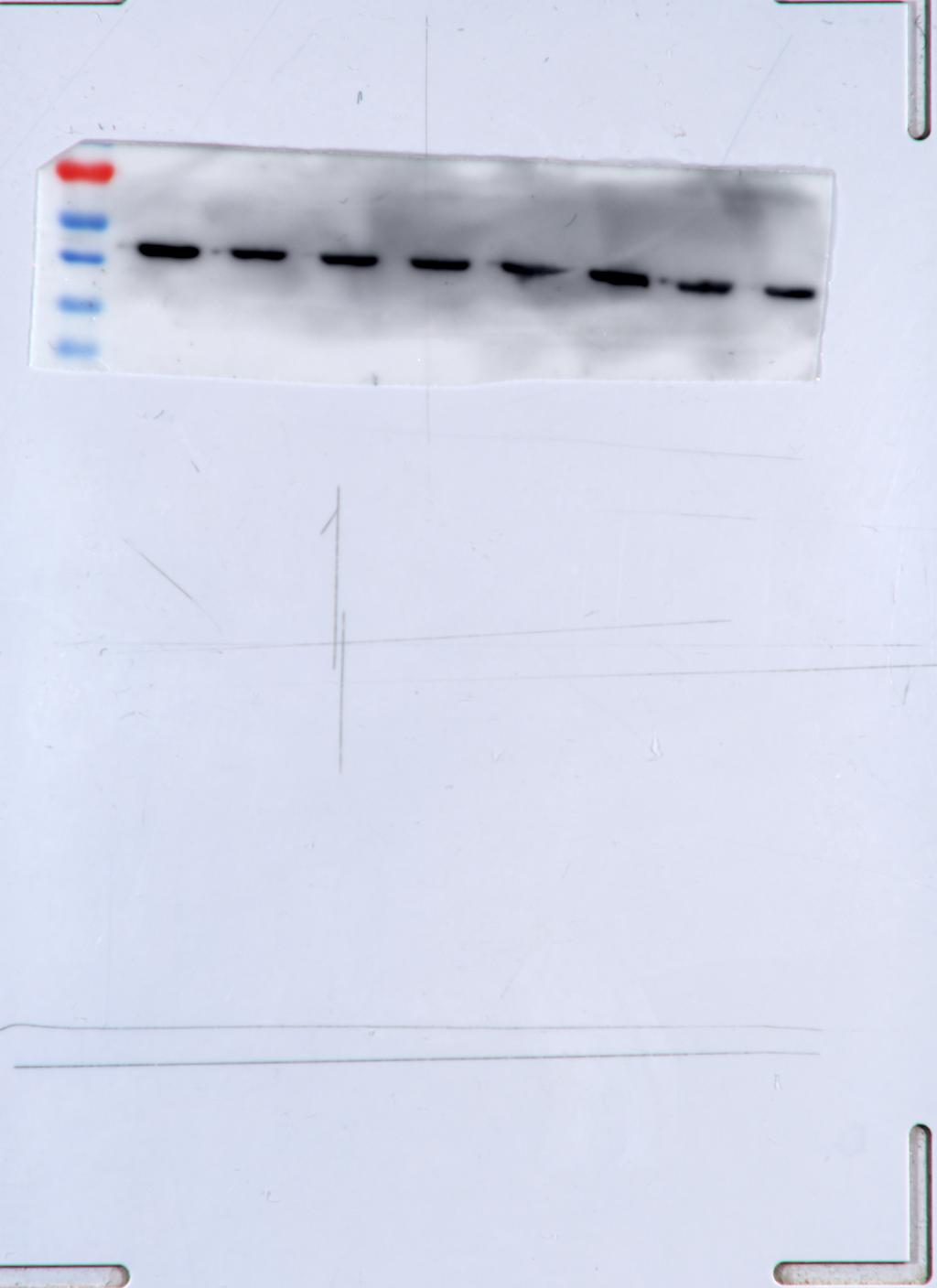

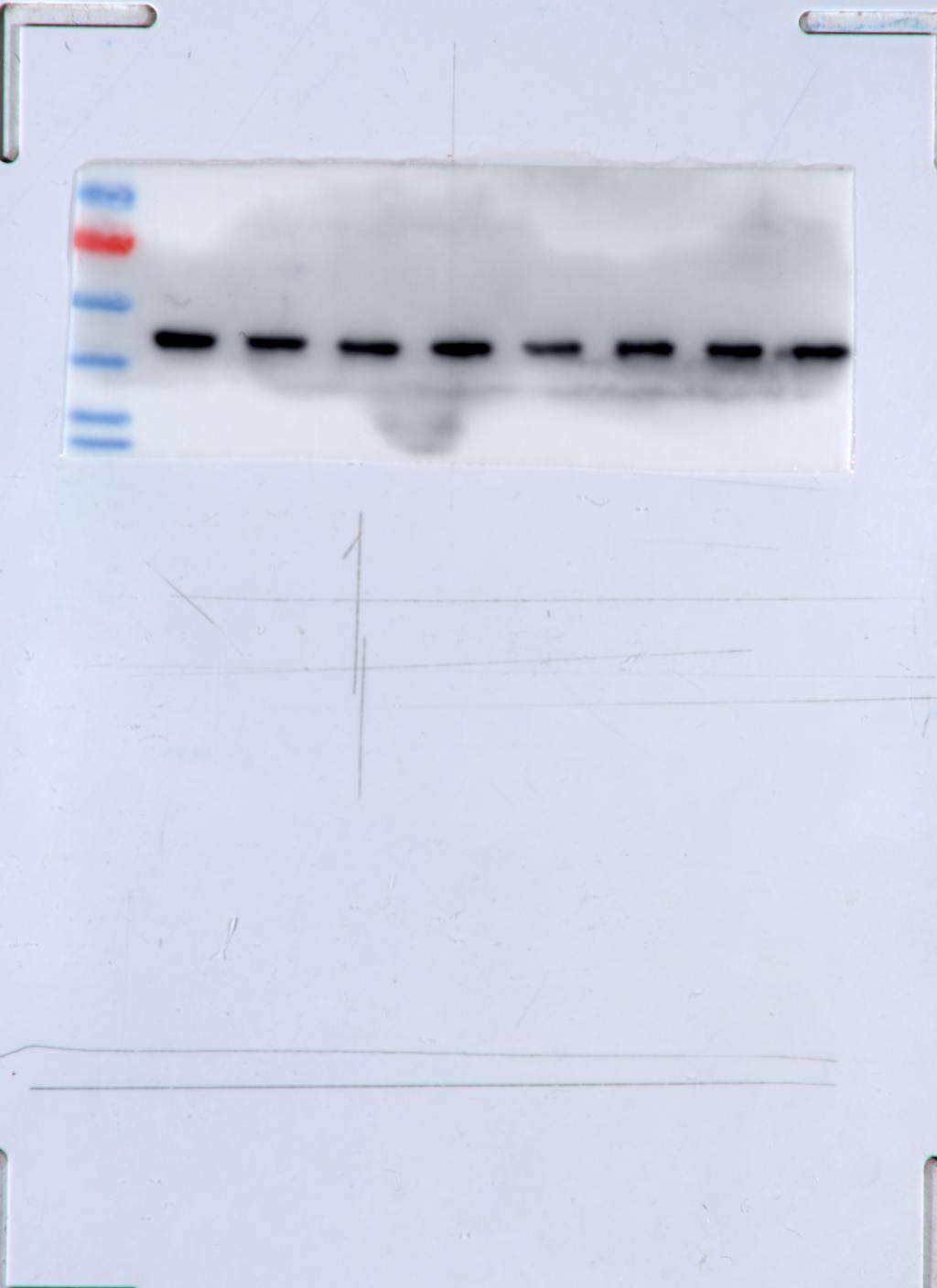

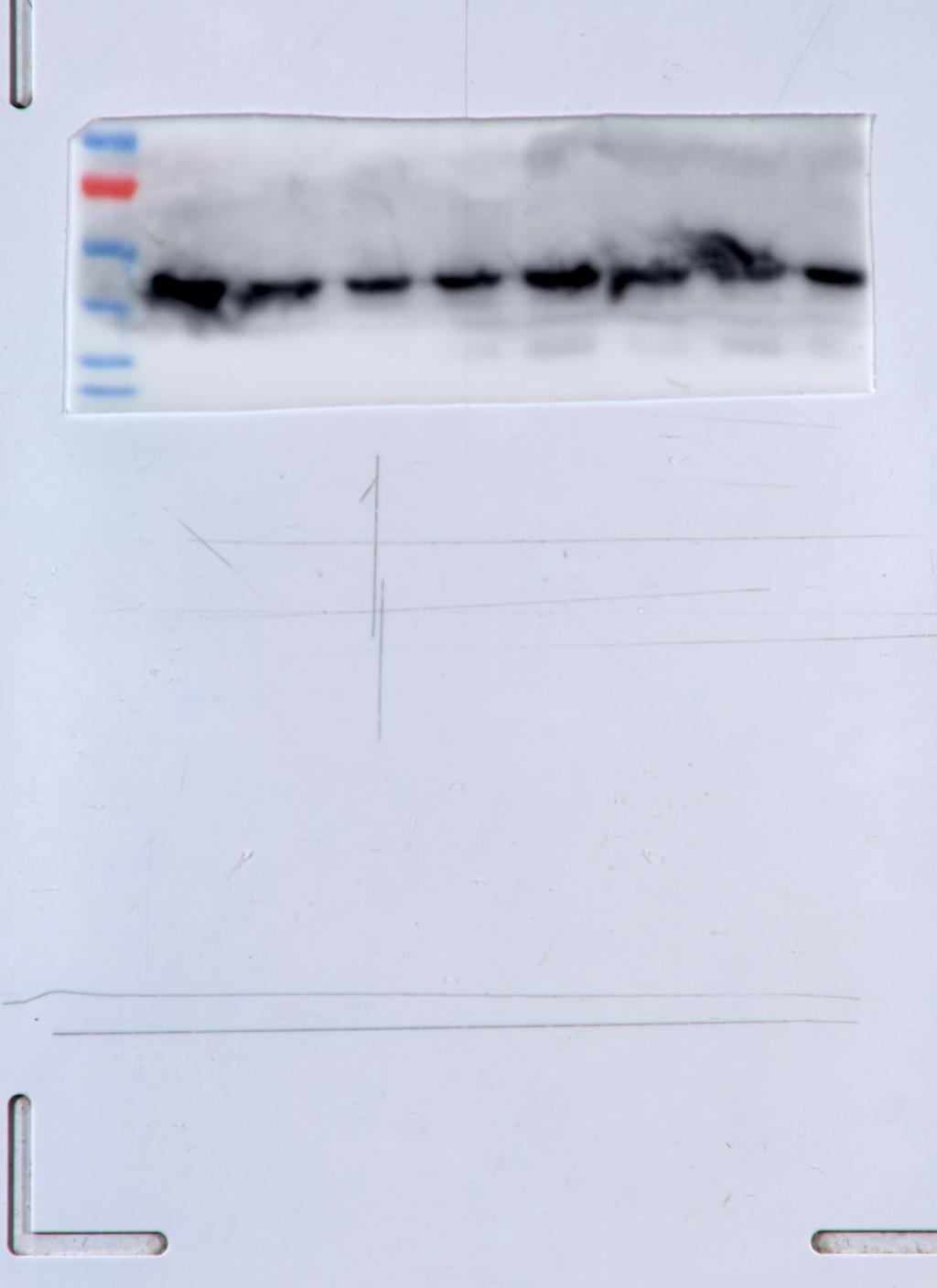


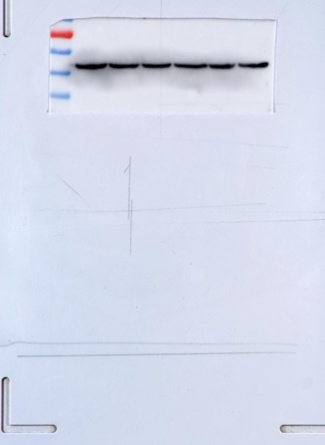

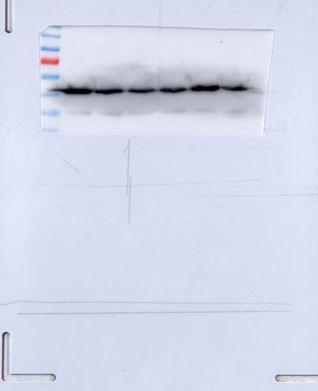

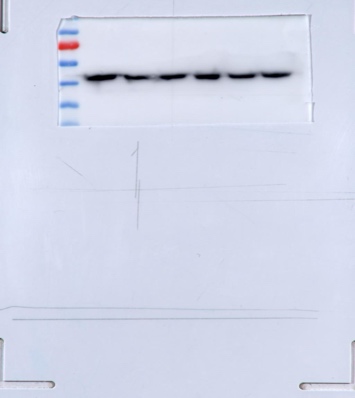


mitochondria：U251NC. U251-3. LN229NC. LN229-3

ATP5A：U251NC. U251-3. LN229NC. LN229-3


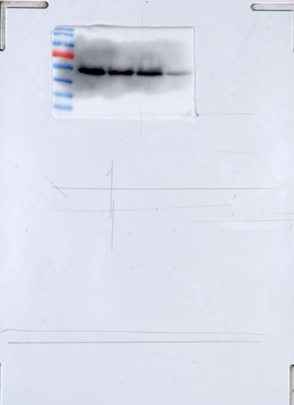

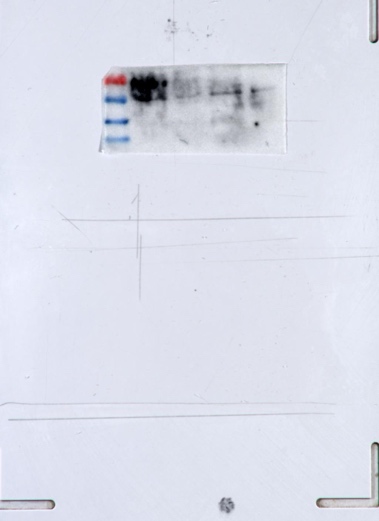

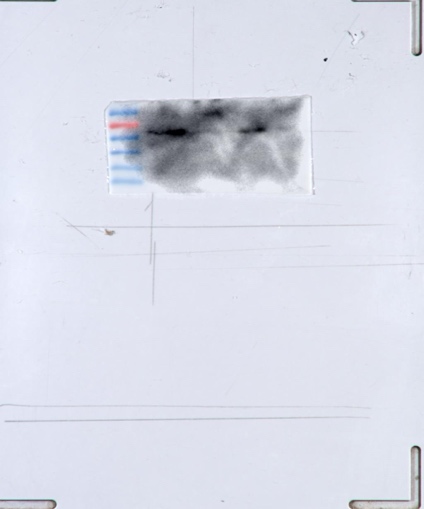


DR1 U251NC. U251-3. LN229NC. LN229-3


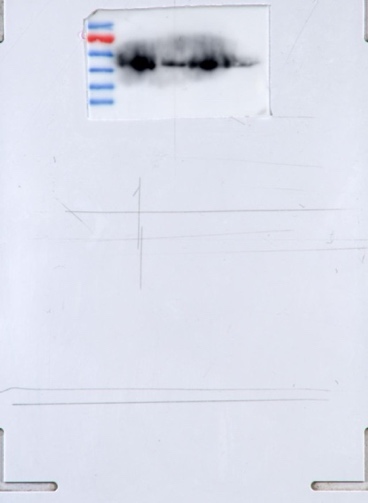

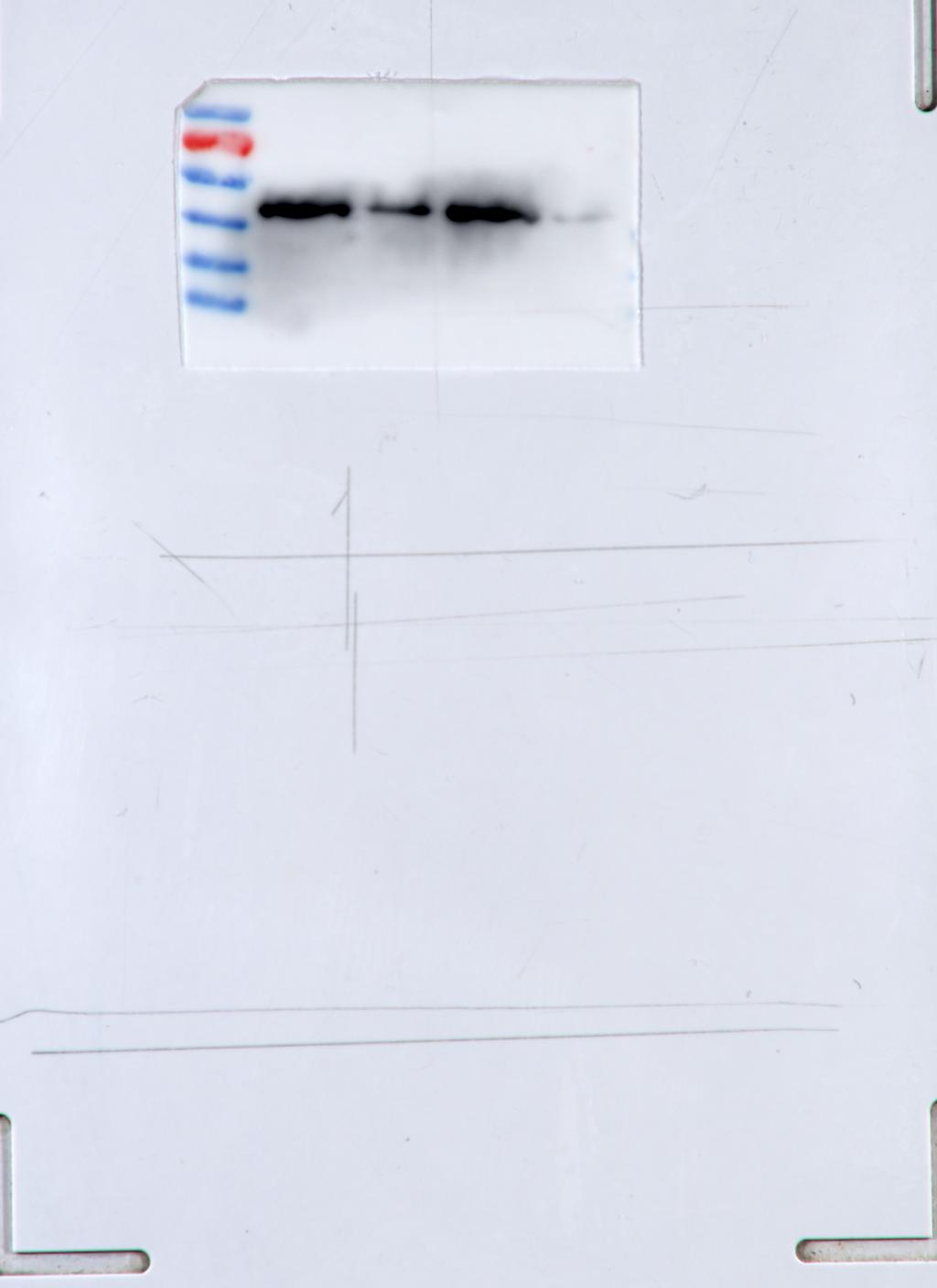

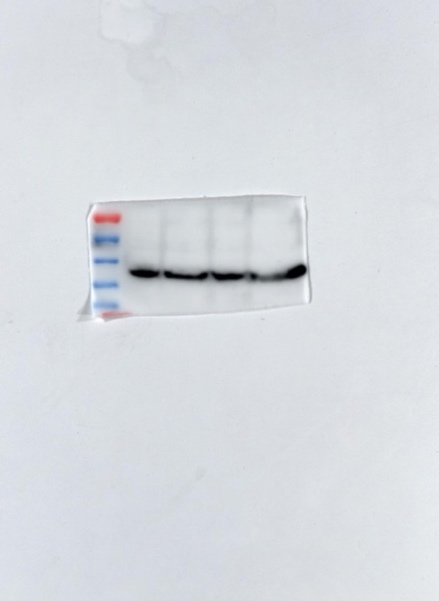


SDHB


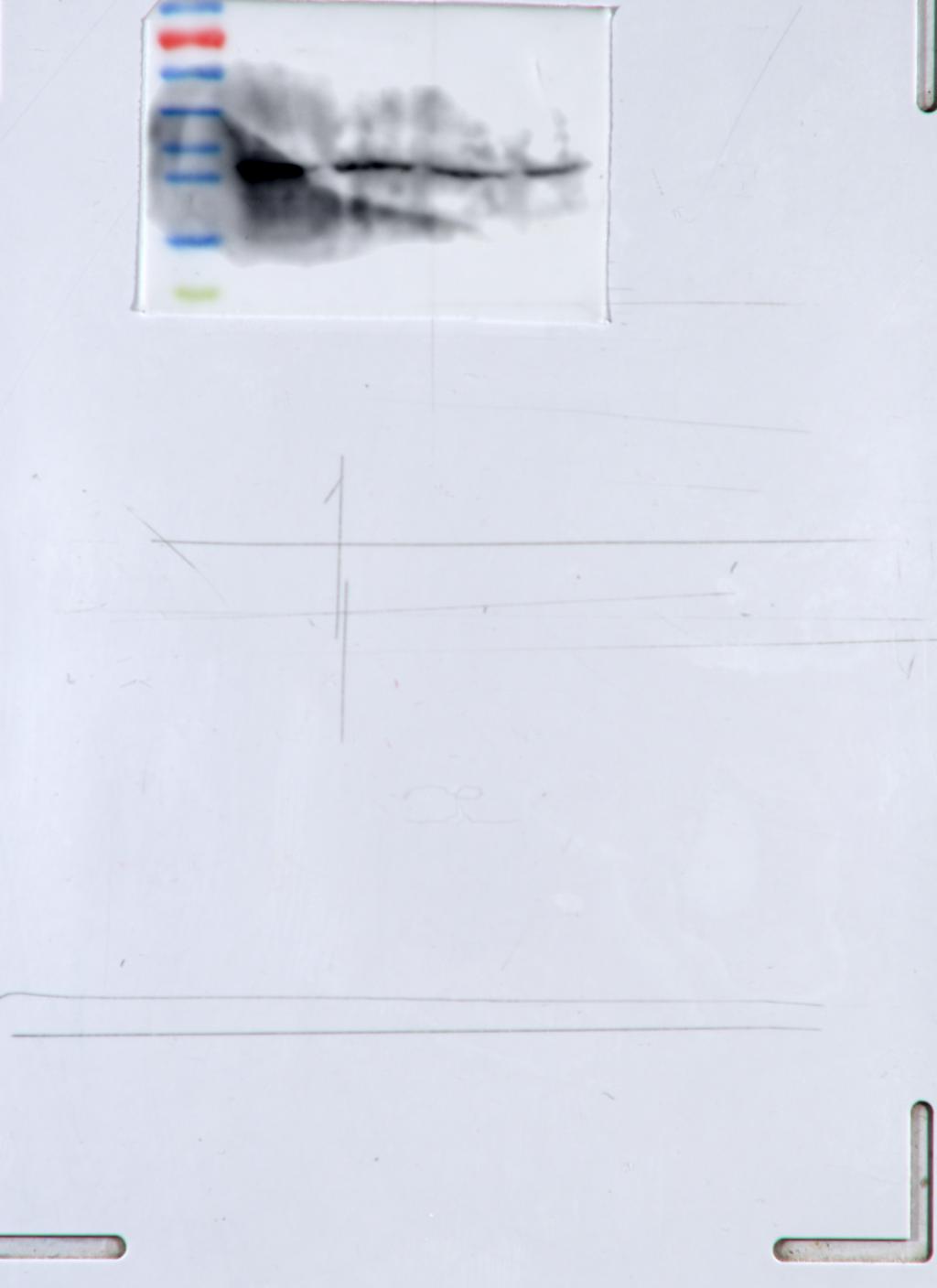

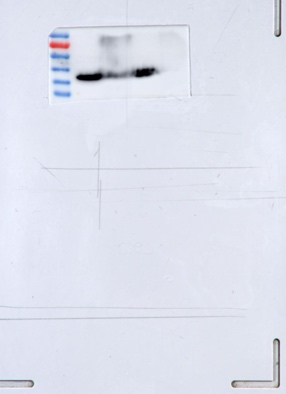

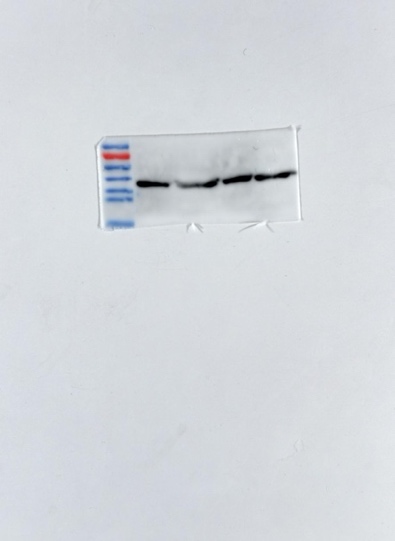


MFN1 U251NC 251-3. LN229NC LN229sh-3


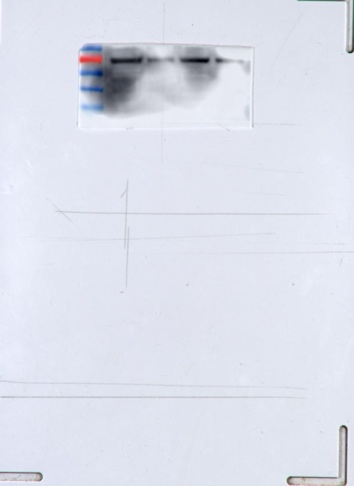

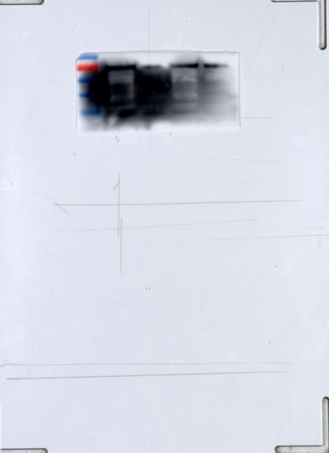

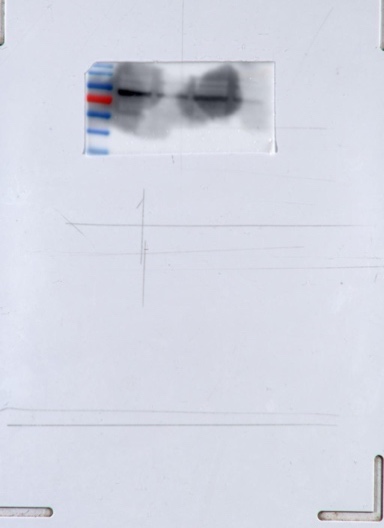

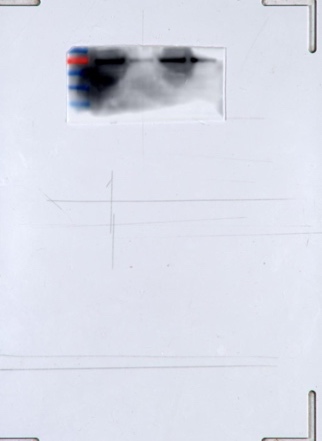


DRP1: U251NC 251-3. LN229NC LN229sh-3


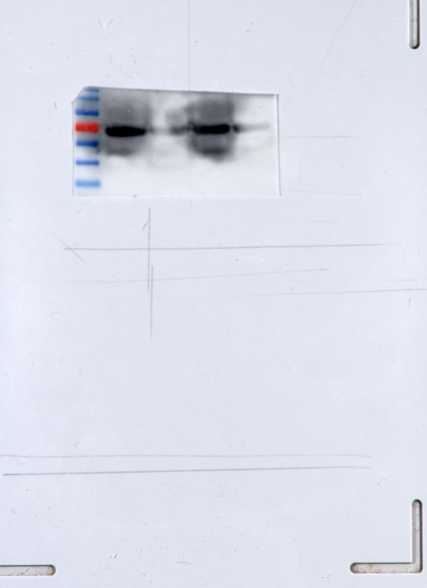

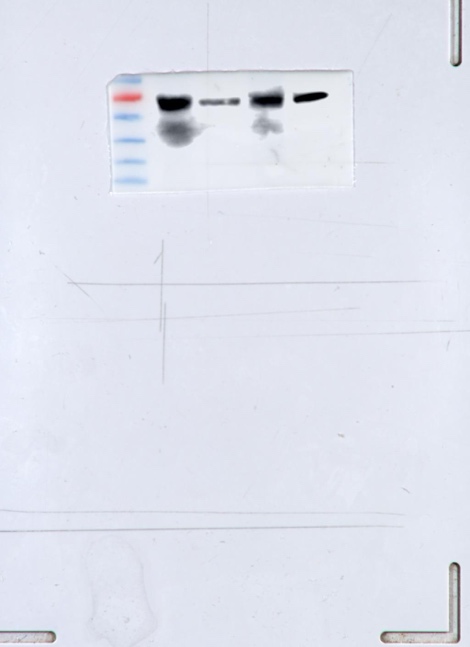

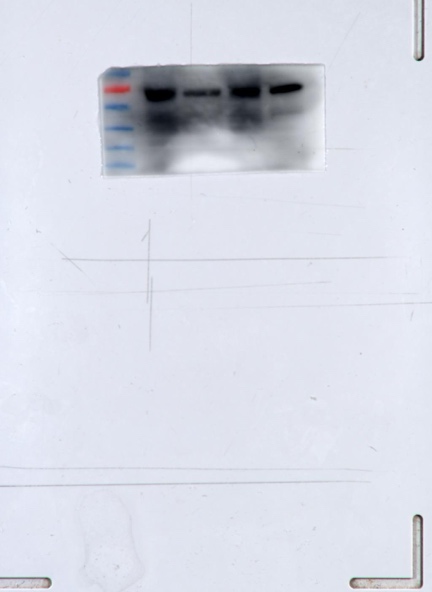


MFN2: U251NC 251-3. LN229NC LN229sh-3


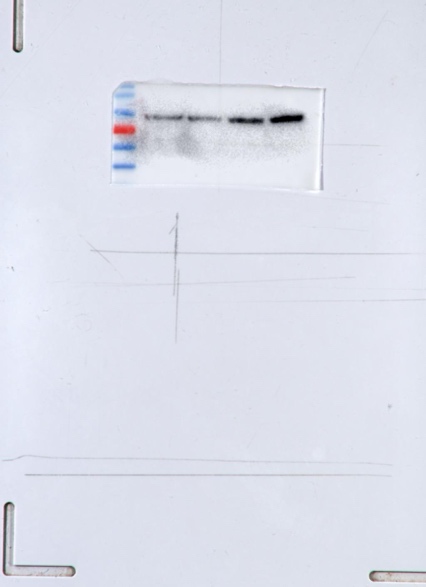

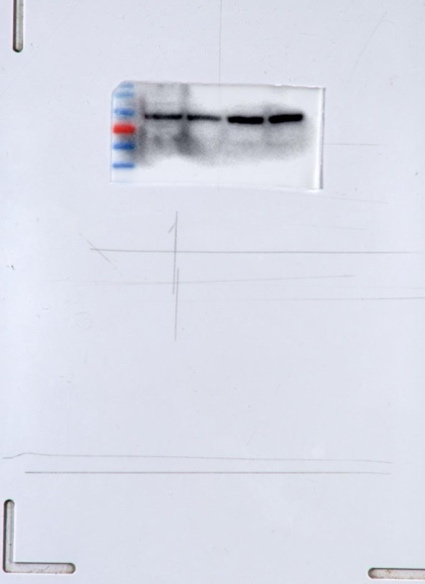

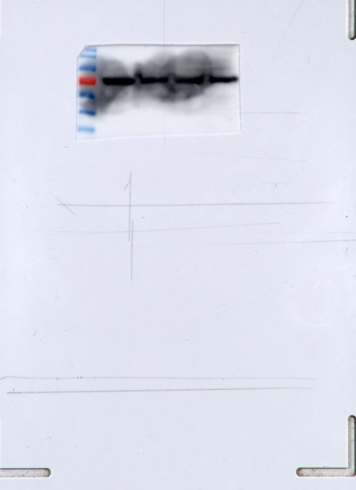

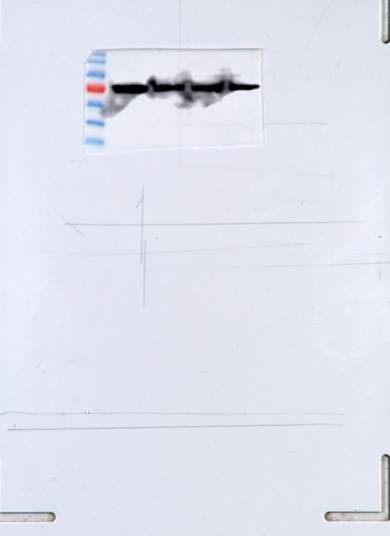


ACTIN:


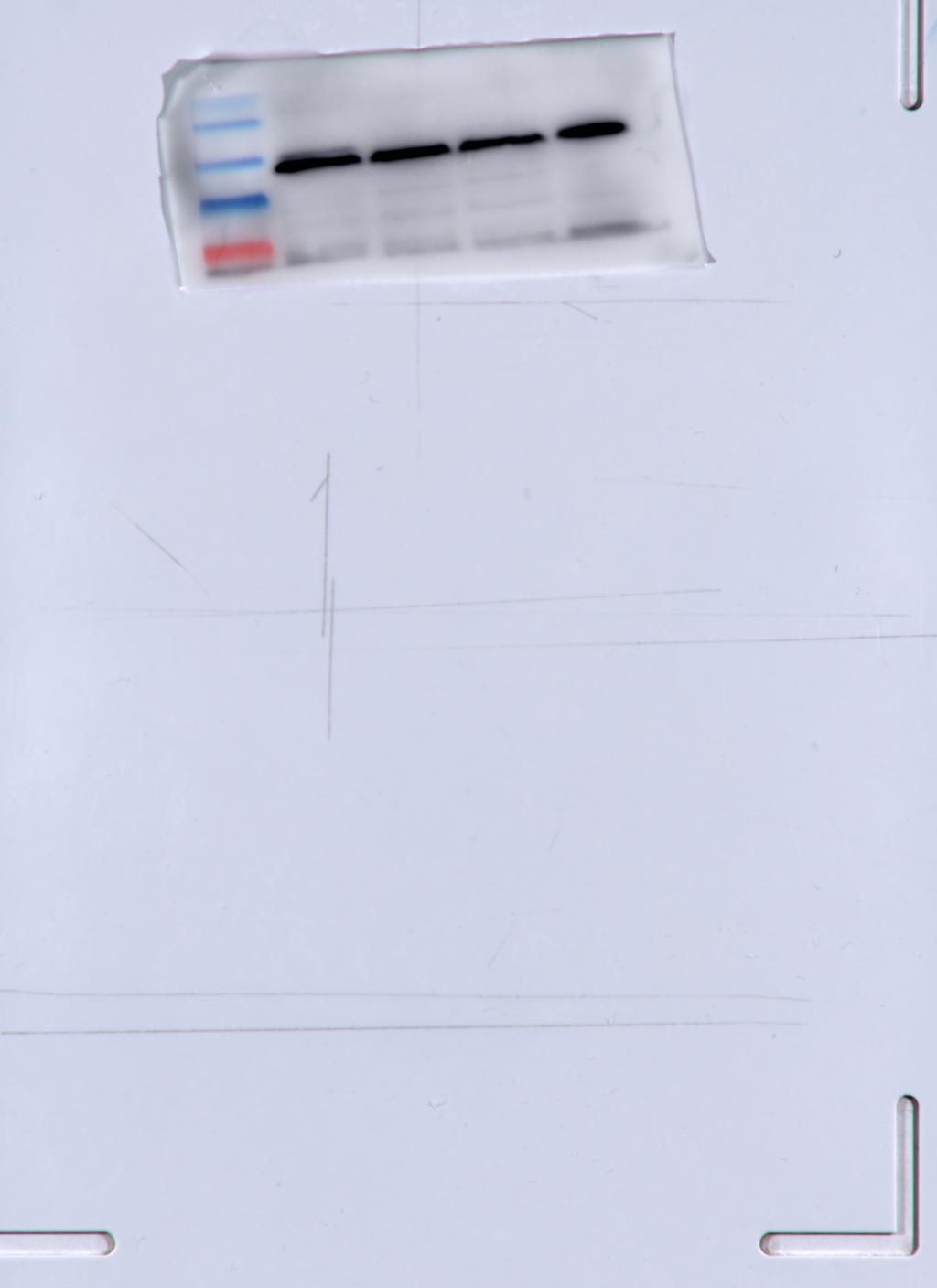

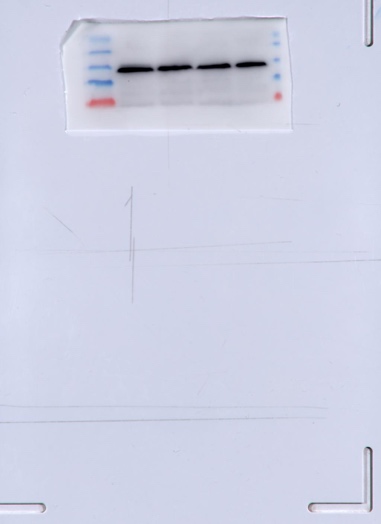

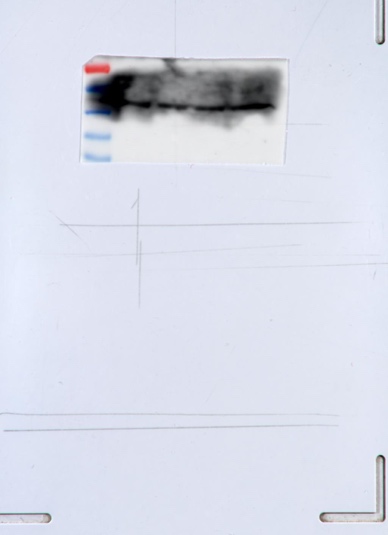

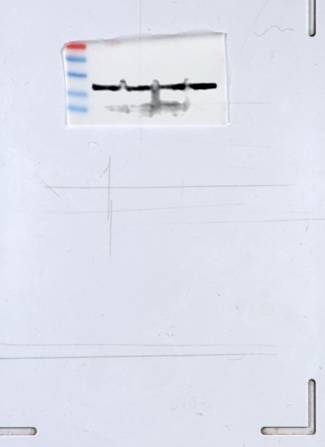


IP:

SUCLG2-LMNA

U251 IP：suclg2


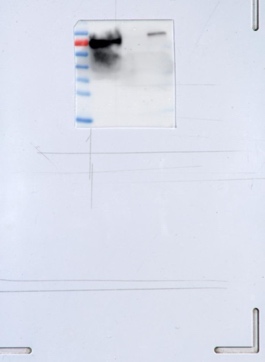

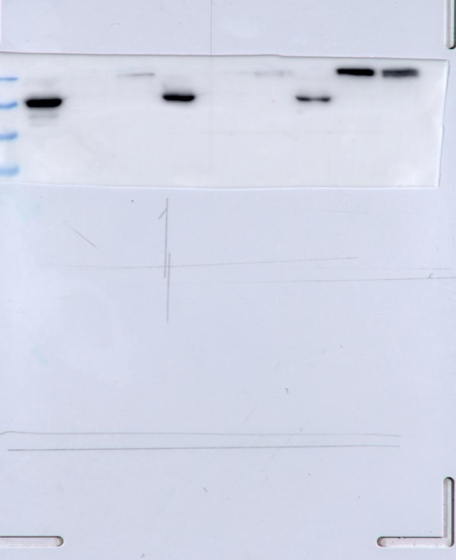

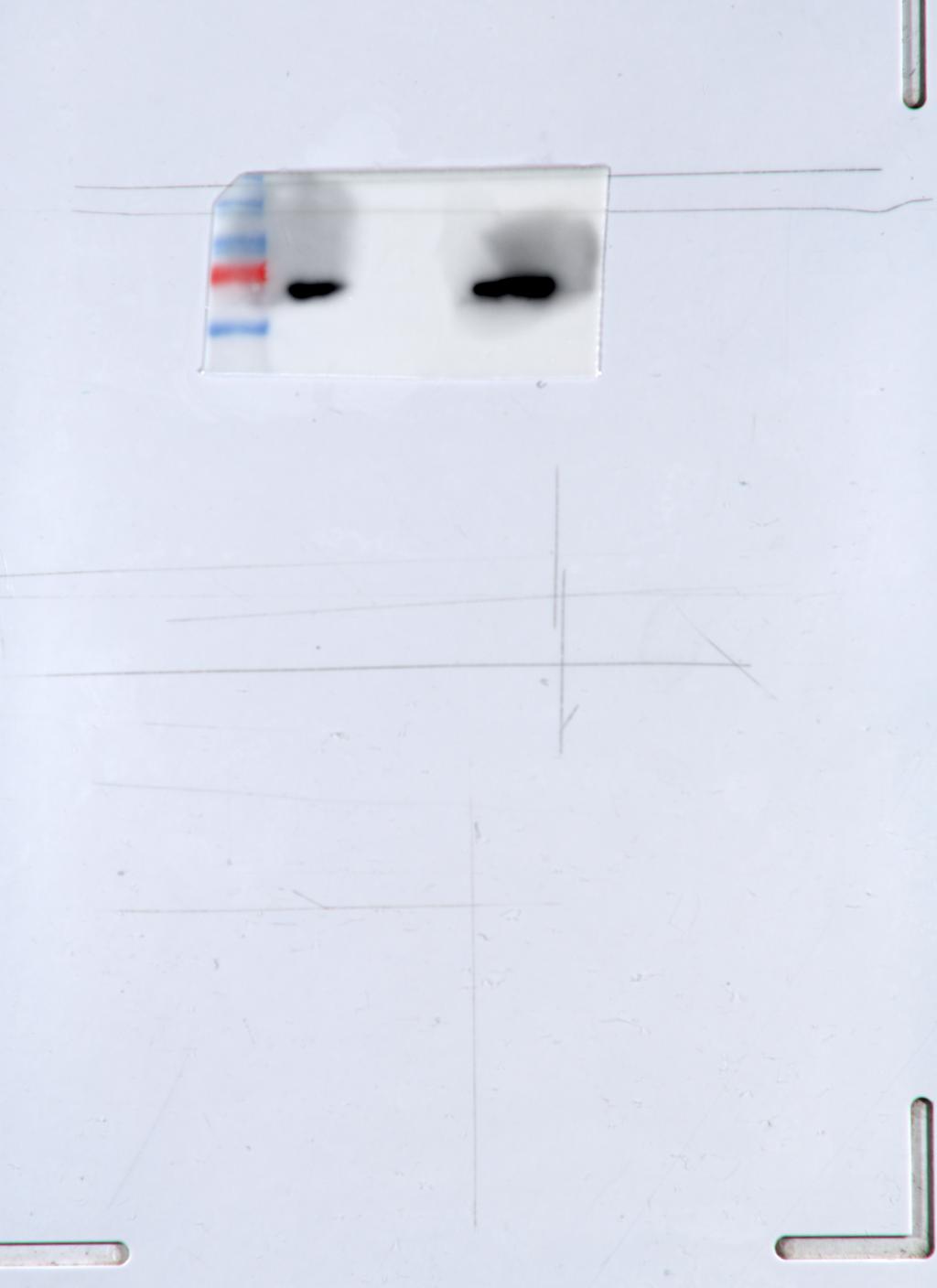

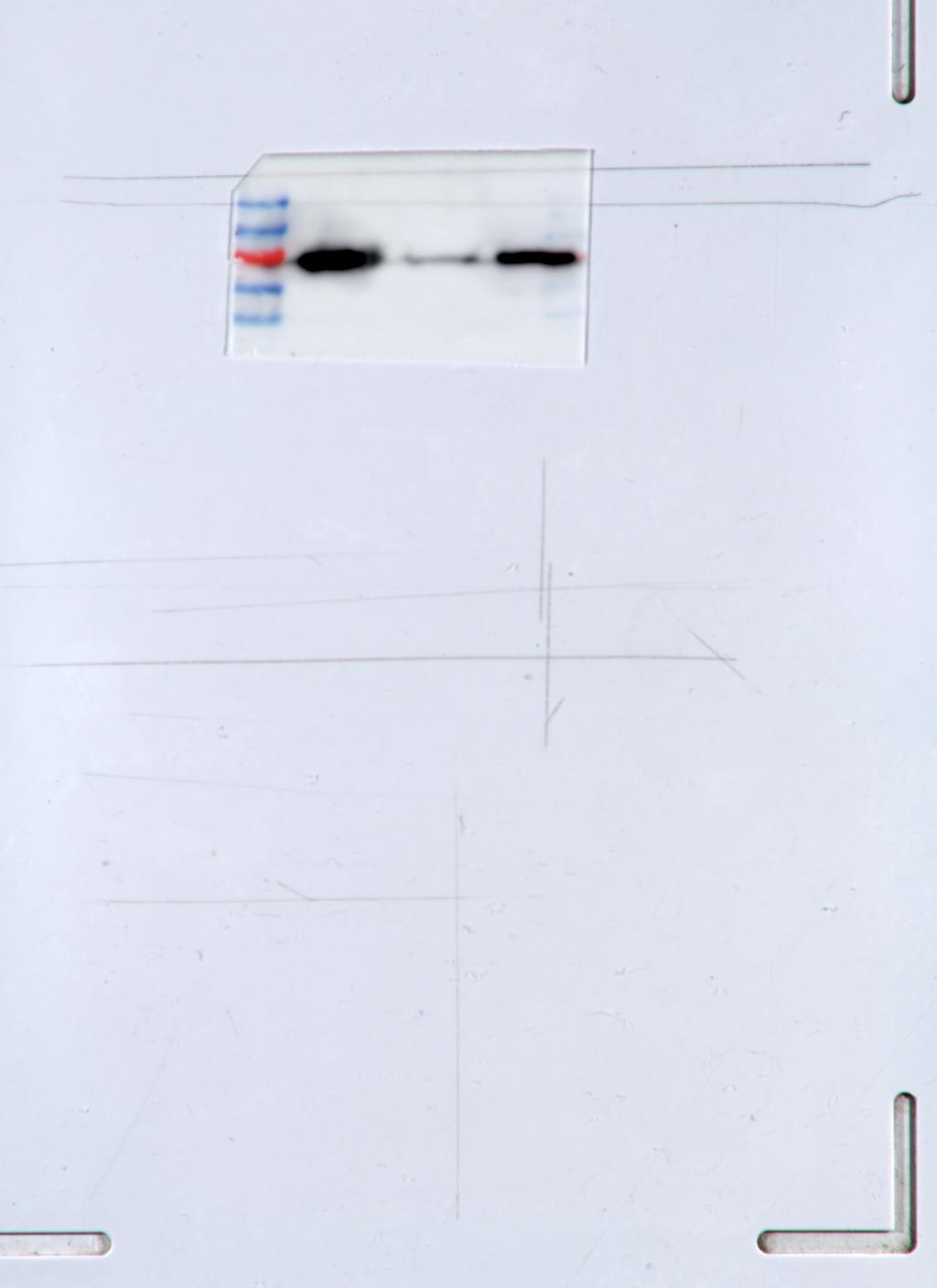
Input IgG suclg2 Input IgG suclg2 Input IgG suclg2 Input IgG LMNA Input IgGLMNA

LN229


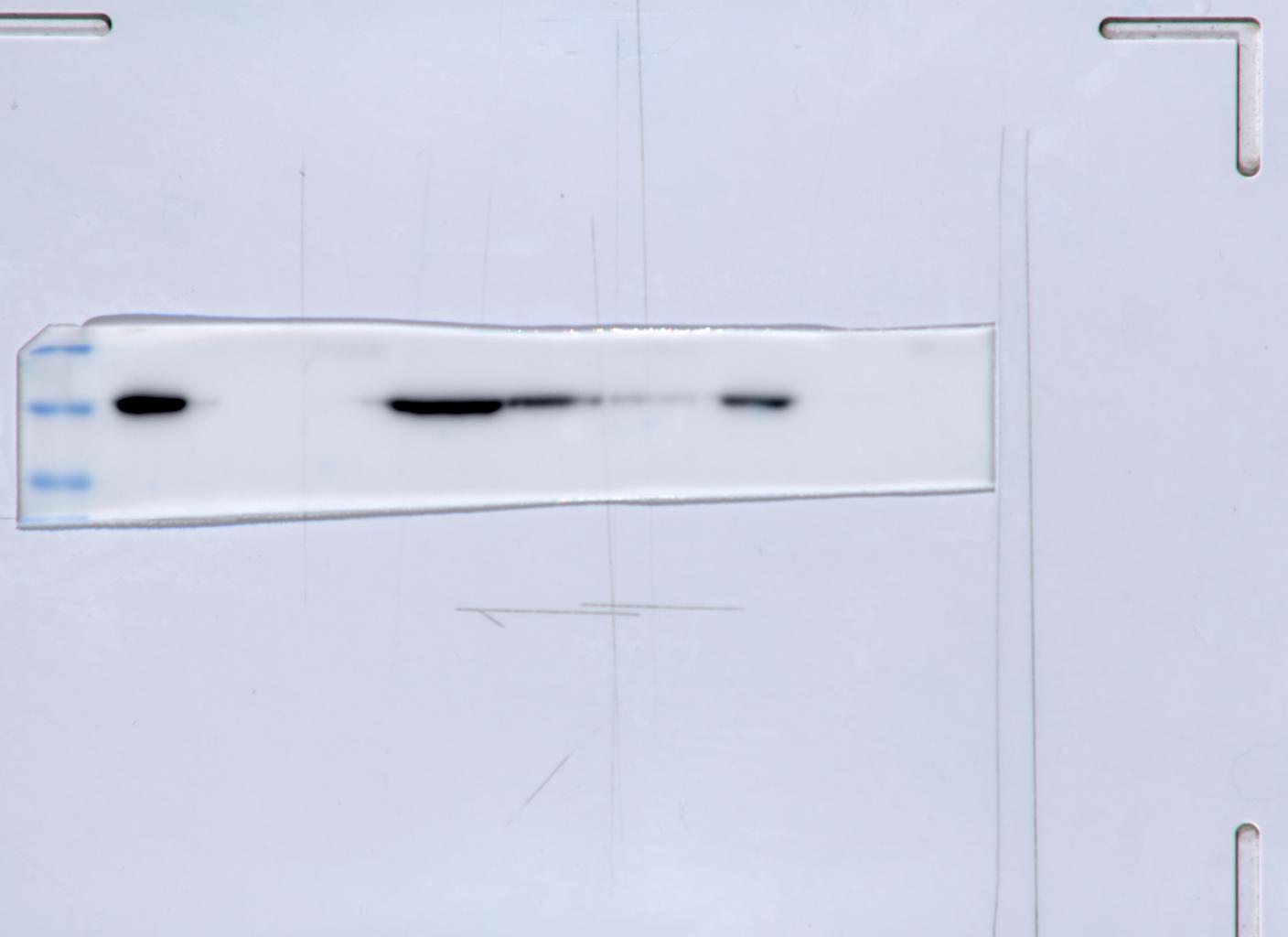

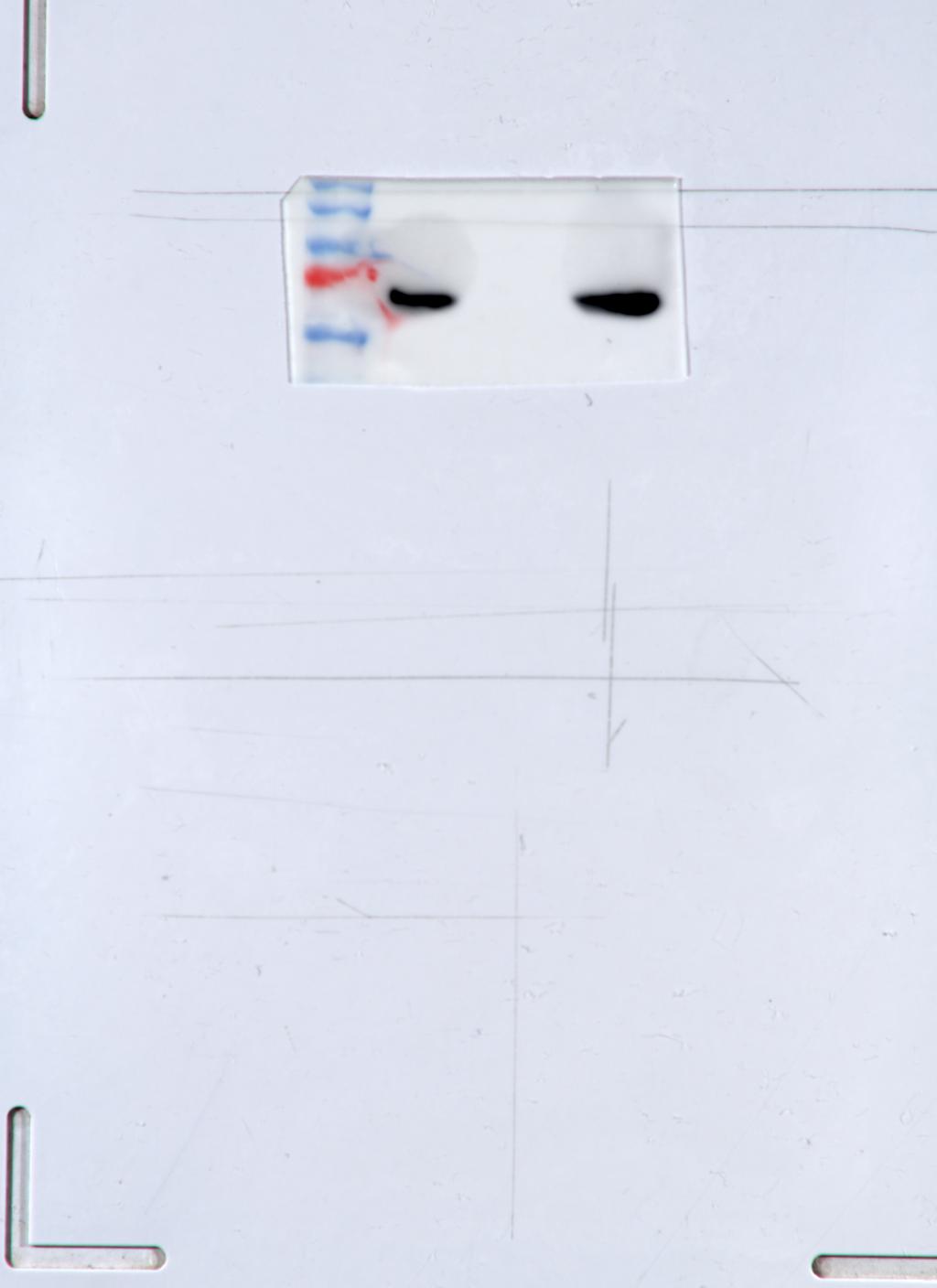

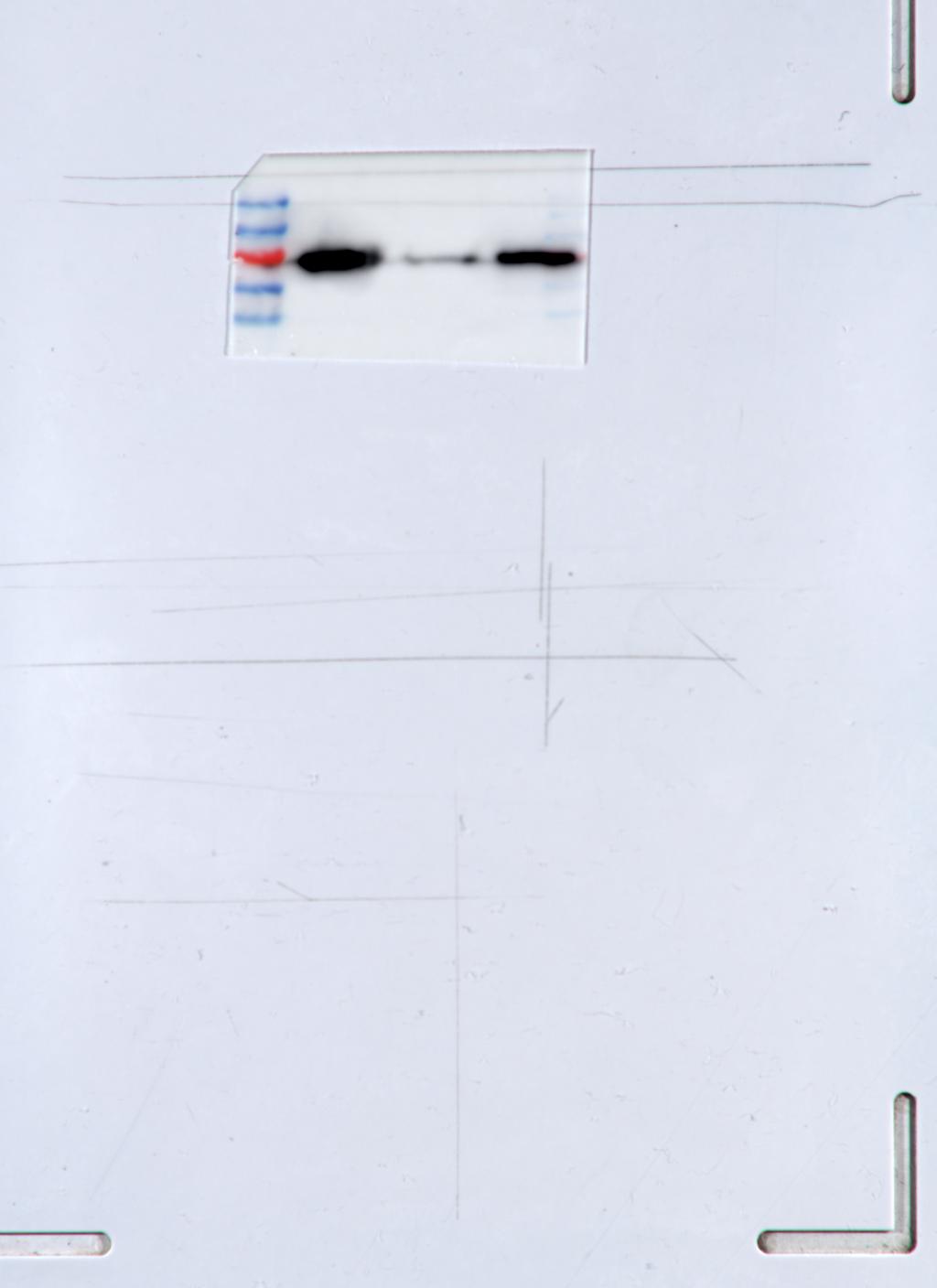
Input IgG suclg2. Input IgG suclg2. Input IgG LMNA Input IgG LMNA

Acetylation

Kac：TSA and NAM+/- LN229 and U251


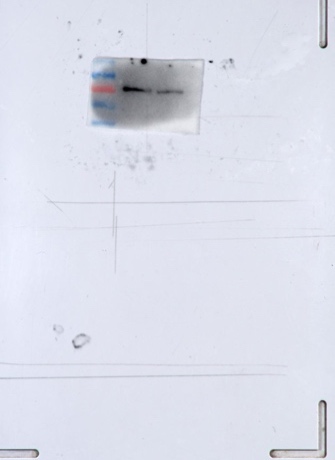

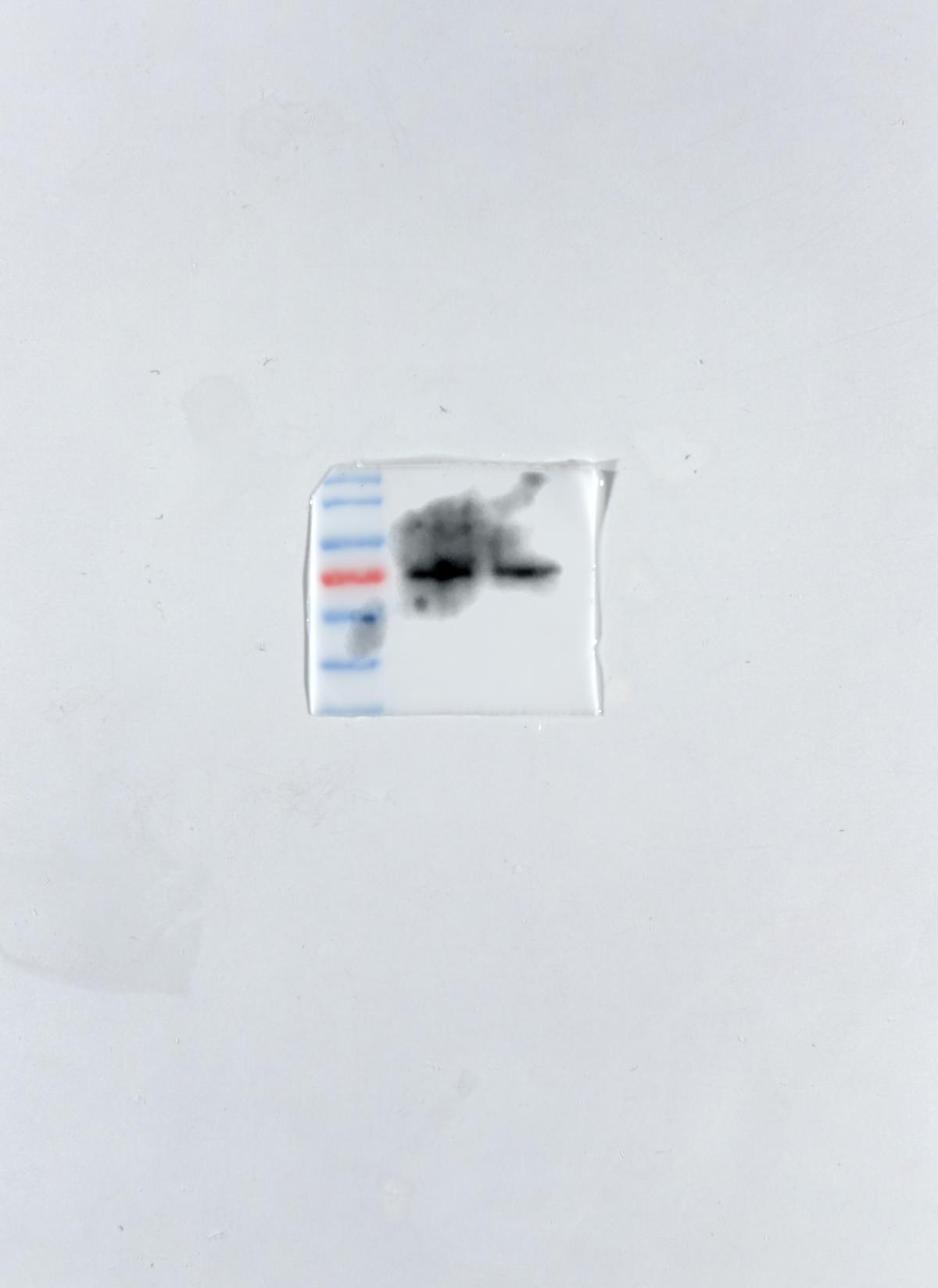

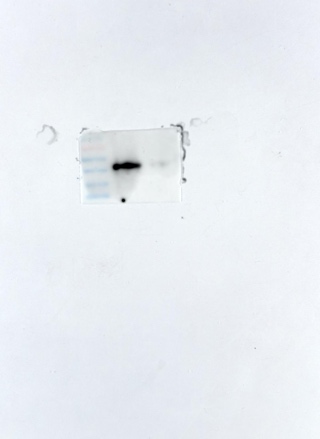

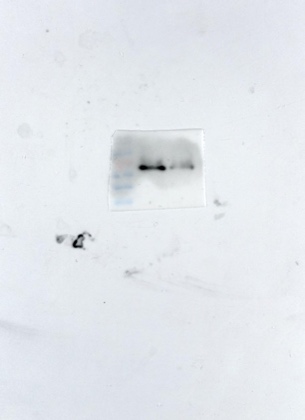

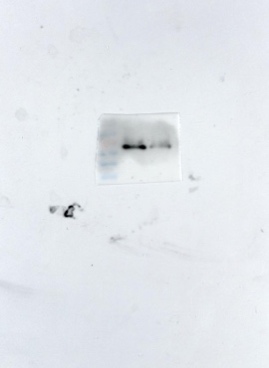

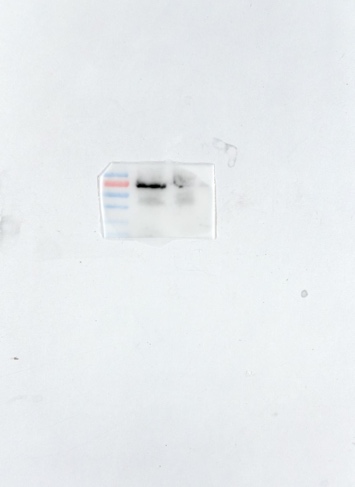

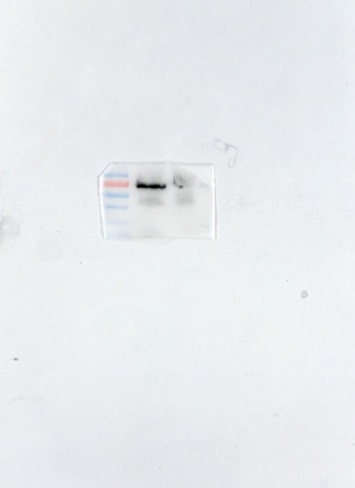

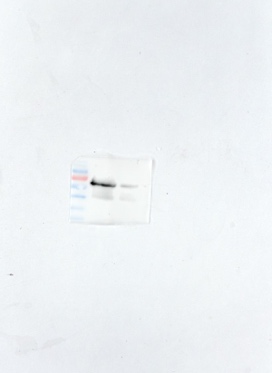

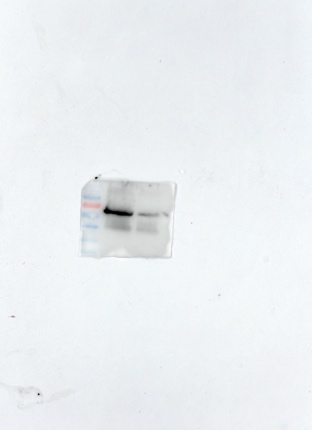


INPUT. TSA and NAM+/-


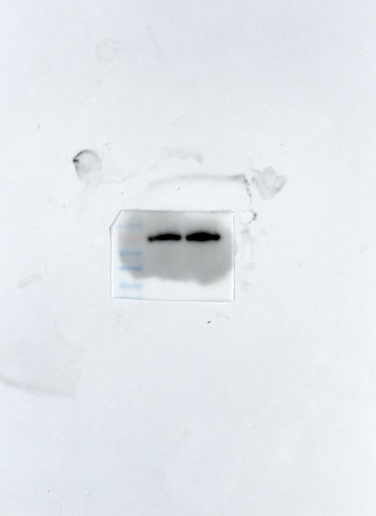

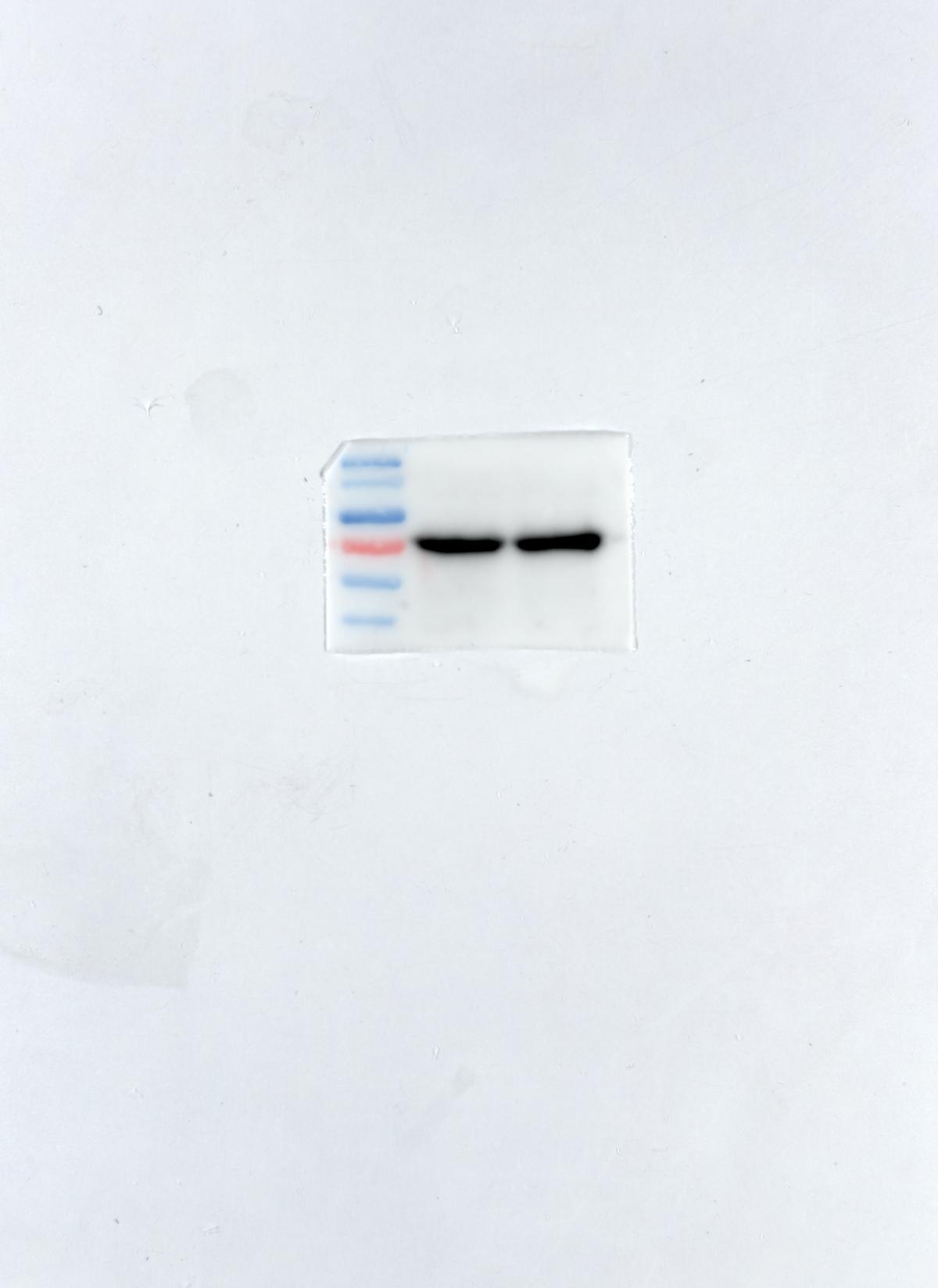

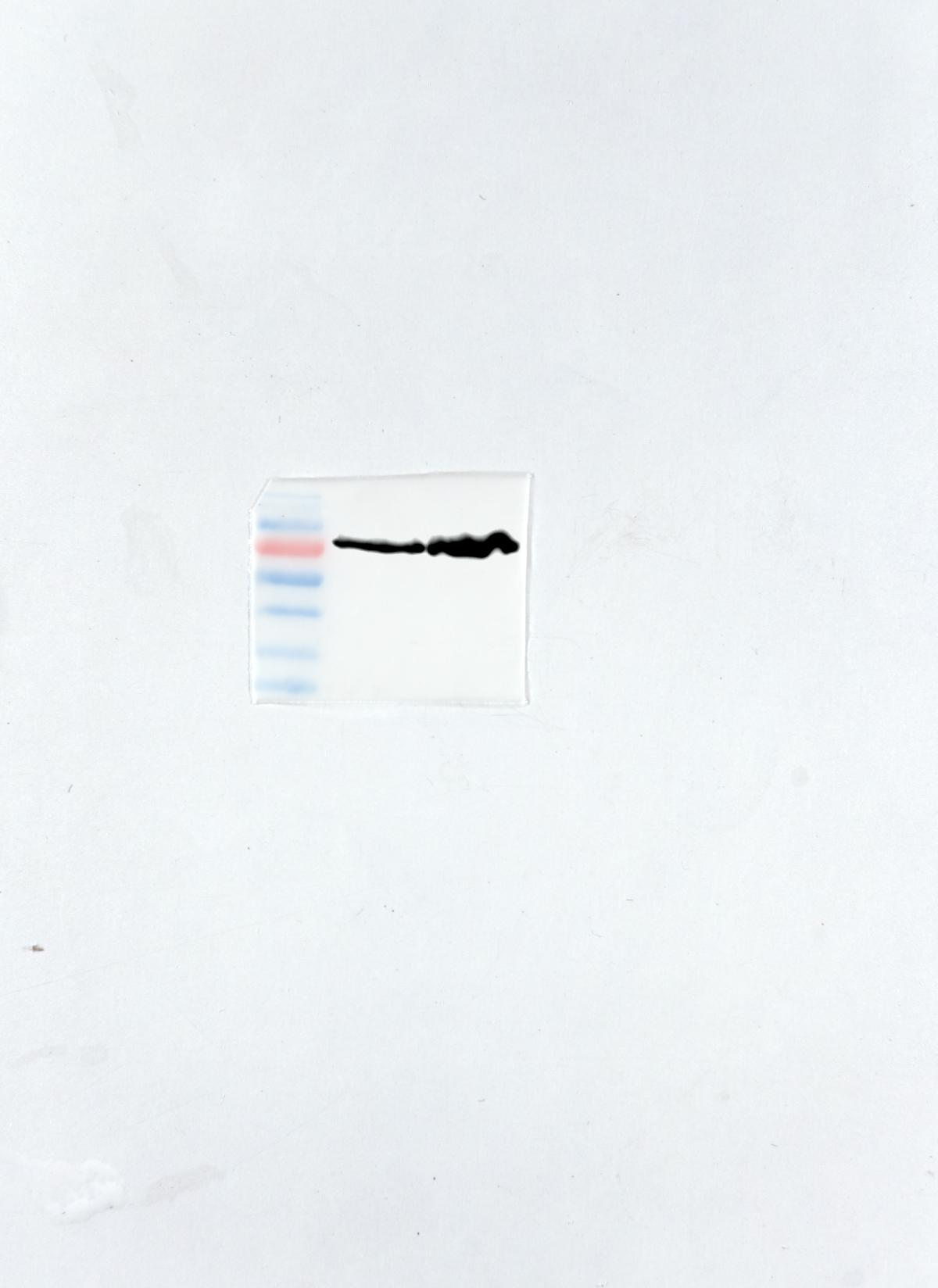


Acetylation site validation ：


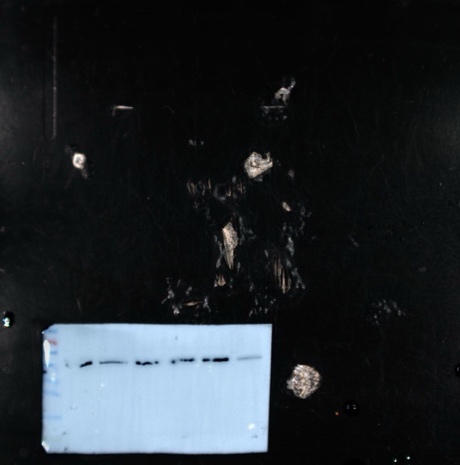

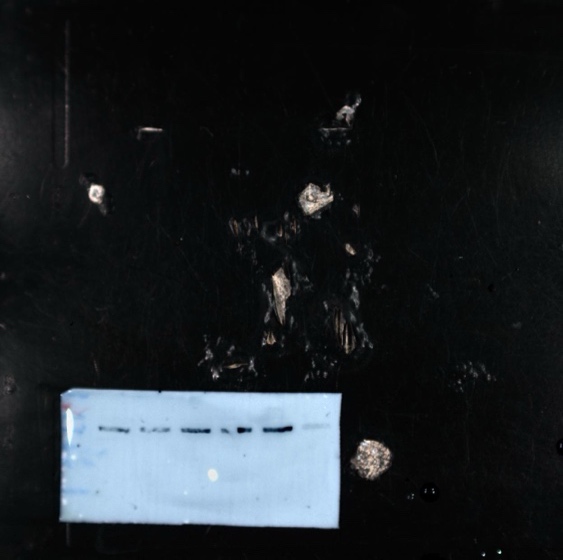


Validation of acetylation function

LMNA：LN229 and U251


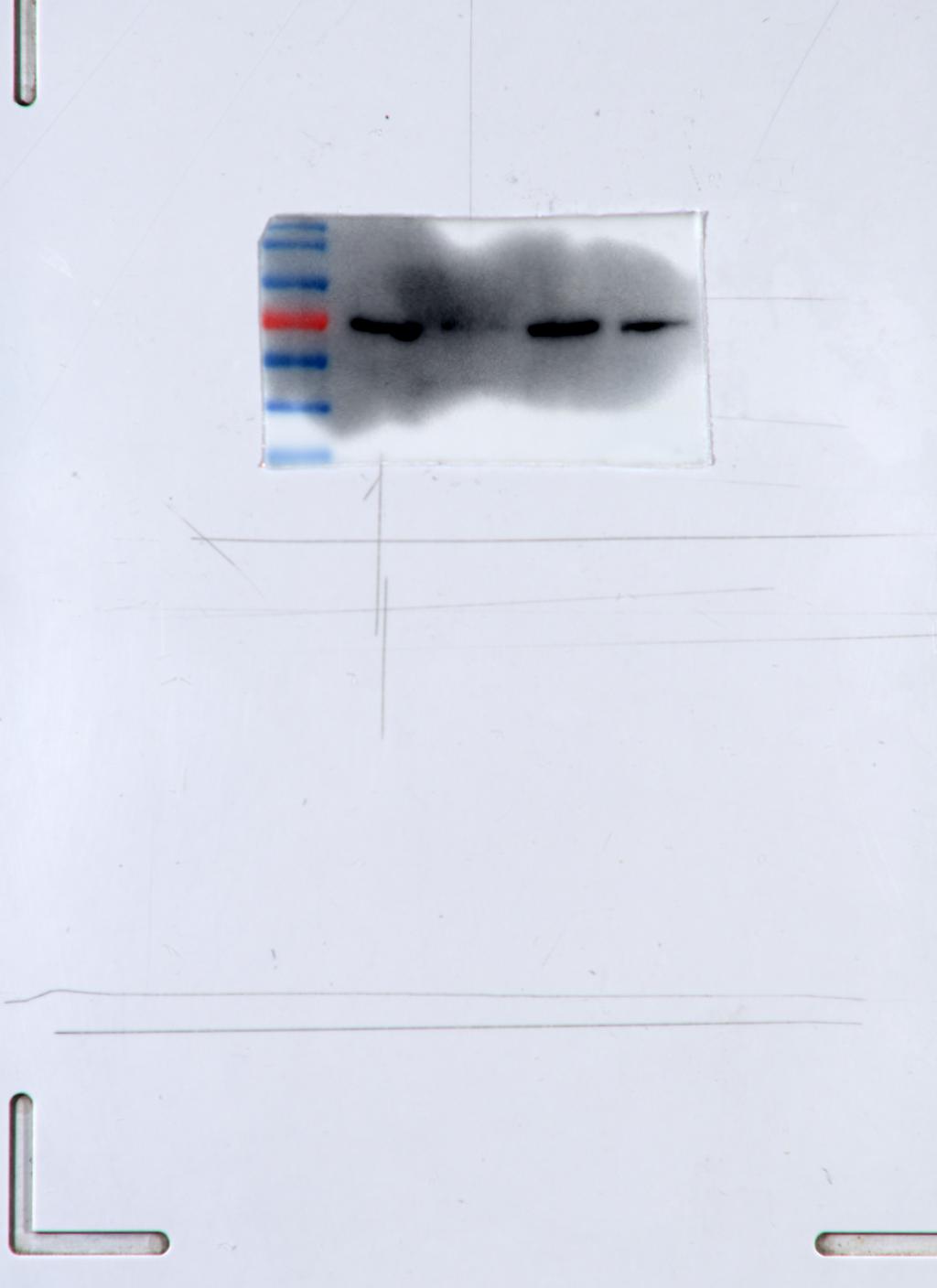

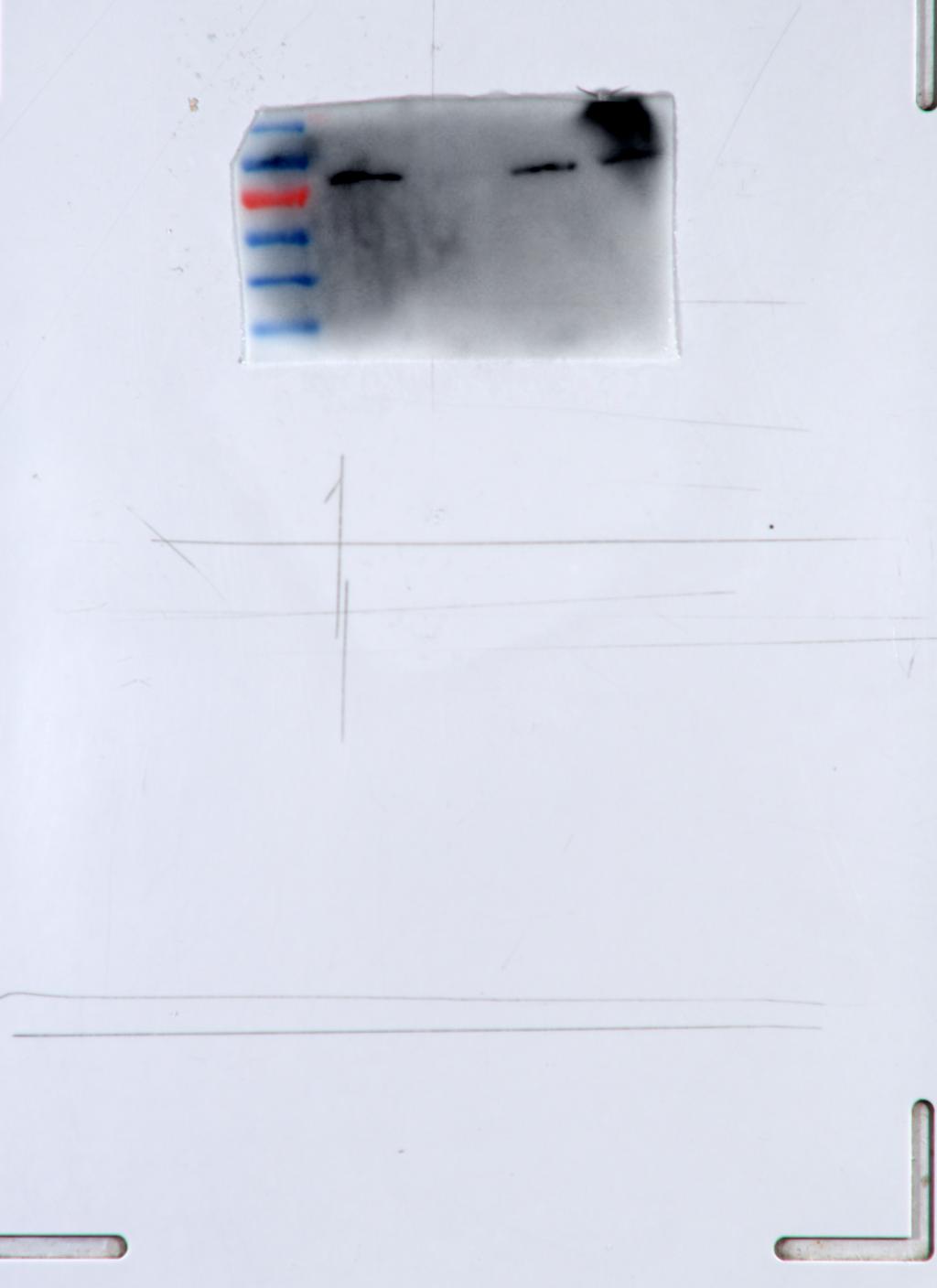

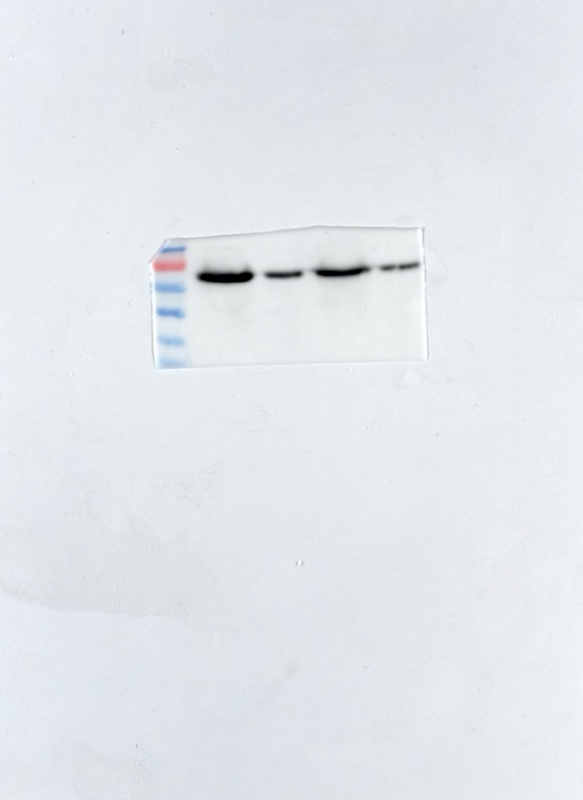


ATP5A


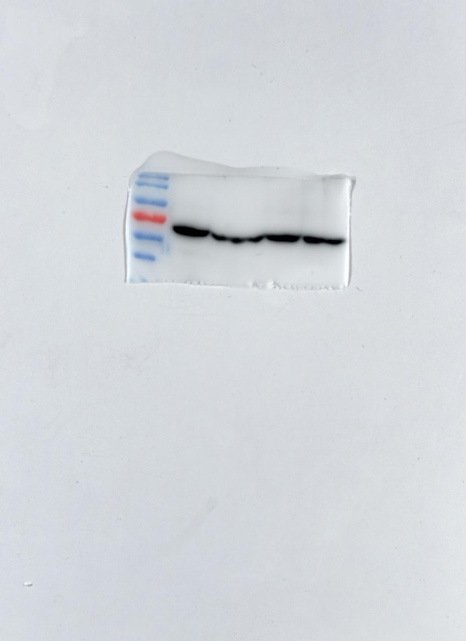

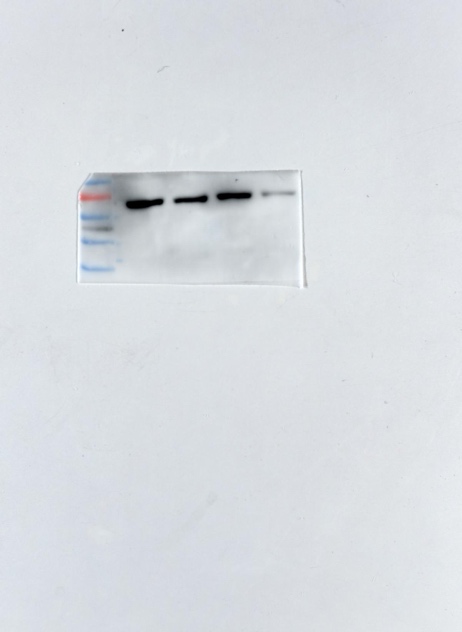

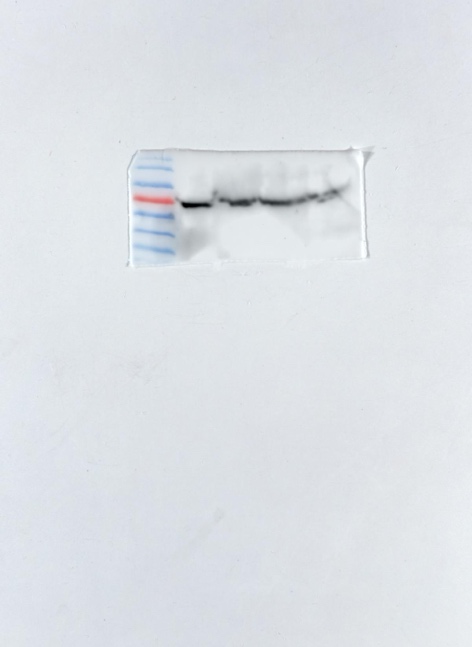

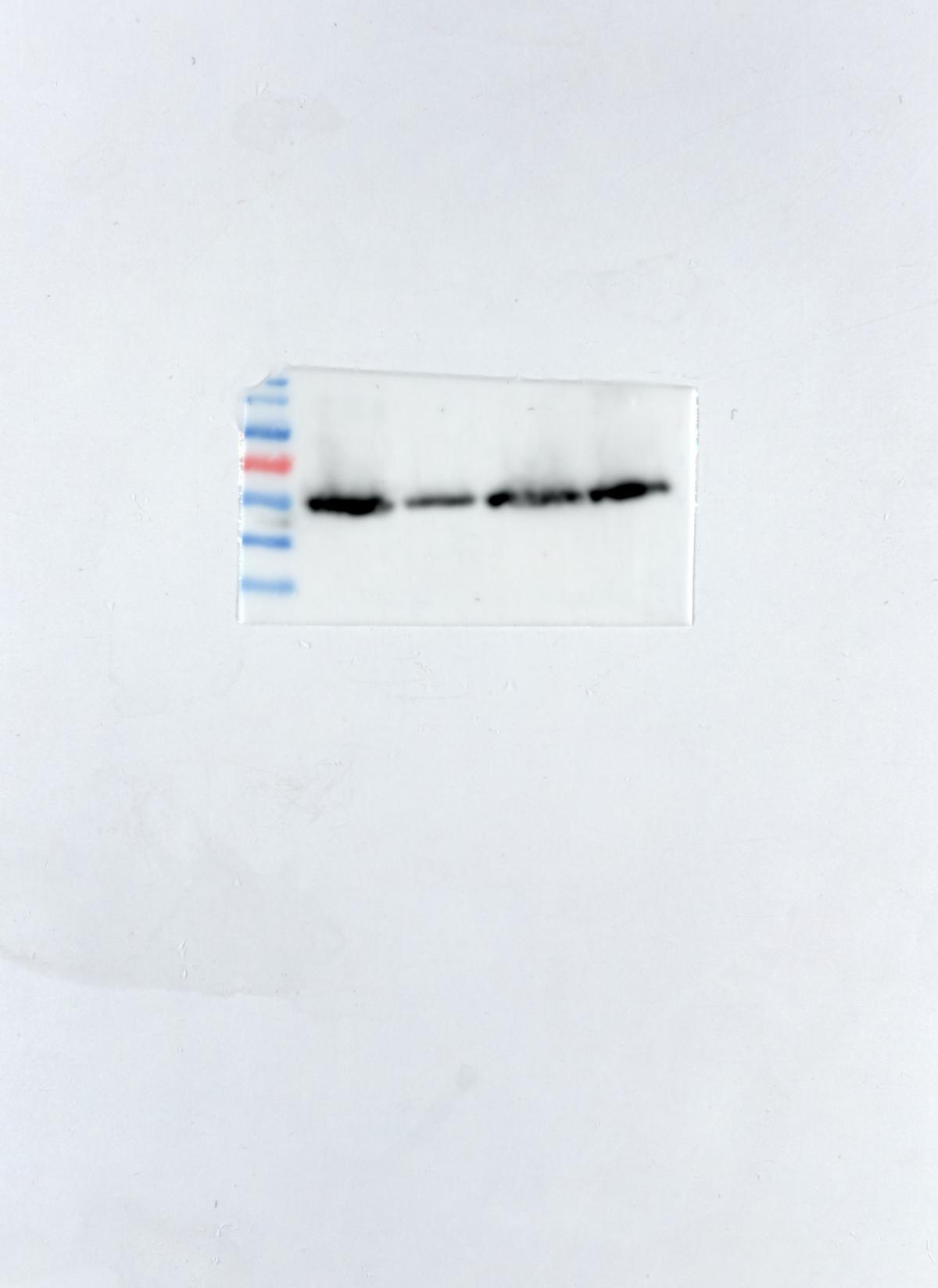


DR1


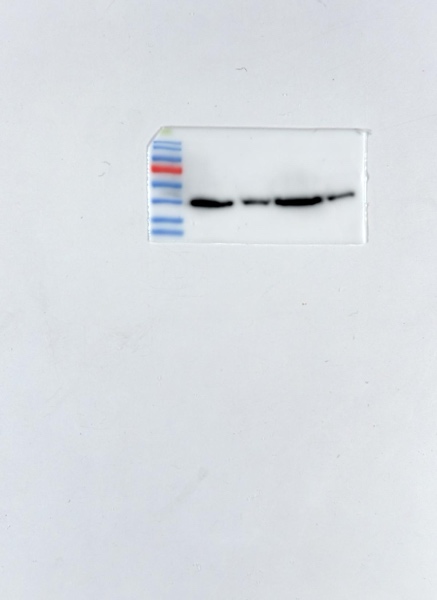

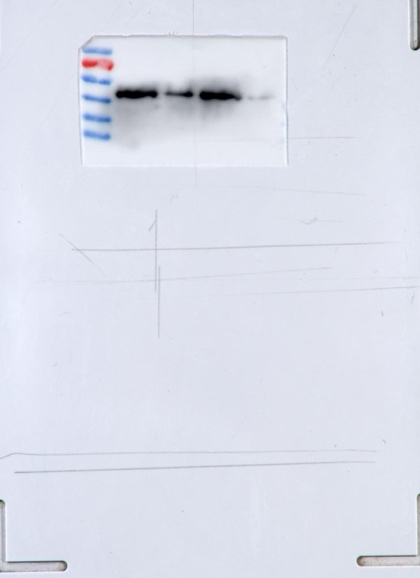

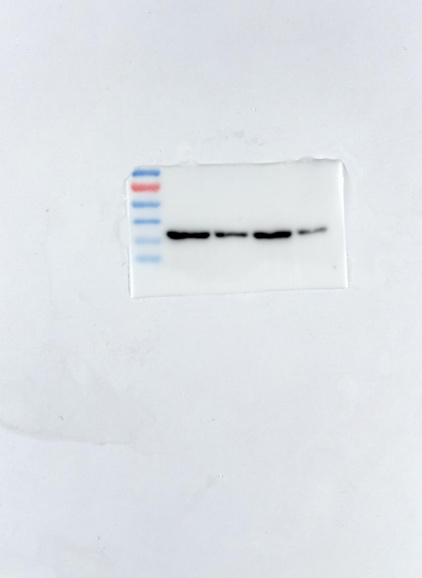


SDHB

Acetylation IP validation

IP

INPUT

Pull-down:

input

IP: SUCLG2-DLAT

U251

Input IgG suclg2 Input IgG suclg2 Input IgG suclg2

Input IgG suclg2 Input IgG suclg2 Input IgG suclg2

Input IgG DLAT Input IgG DLAT Input IgG DLAT

LN229

Input IgG suclg2 Input IgG suclg2 Input IgG suclg2

Input IgG suclg2 Input IgG suclg2 Input IgG suclg2

Input IgG suclg2 Input IgG suclg2 Input IgG suclg2

Input IgG DLAT Input IgG DLAT Input IgG DLAT

DLAT:

U251 NC sh-3 LN229 NC sh-3

Actin:

tumor tissue（peripheral- core）: P P P P C C C C.

HIF-1a P P P P C C C C.

L-Lac P P P P C C C C

actin P P P P C C C C

Lactic acid in tumor tissues of different grades：

Normal normal II II. III III. IV IV

Actin：

U251

HIF-1+ suclg2NC/sh +L-Lac （IN-3）

HIF-1

L-Lac

SUCLG2

actin

LN229 HIF-1.+ suclg2NC/sh +L-Lac （IN-3）

HIF-1

L-Lac

suclg2

actin

**Add lactic acid**：

U251NC. U251-3

Suclg2 L-Lac Control D-lac L-lac Control D-lac L-lac

L-Lac. Control D-lac L-lacControl D-lac L-lac

Actin Control D-lac L-lacControl D-lac L-lac

D-Lac. Control D-lac L-lac Control D-lac L-lac

Actin Control D-lac L-lacControl D-lac L-lac

LN229NC. LN229-3

Suclg2：

Control D-lac L-lac Control D-lac L-lac

L-Lac Control D-lac L-lac Control D-lac L-lac

D-Lac. Control D-lac L-lacControl D-lac L-lac

Actin：

Control D-lac L-lac Control D-lac L-lac

Rescue（ Add lactic acid）：

PCNA： U251 U251NC sh-3 LN229 LN229NC sh-3

BAX： U251 U251NC sh-3 LN229 LN229NC sh-3

Bcl-2 ：U251 U251NC sh-3 LN229 LN229NC sh-3

D1： U251 U251NC sh-3 LN229 LN229NC sh-3

caspase3 U251 U251NC sh-3 LN229 LN229NC sh-3

Actin： U251 U251NC sh-3 LN229 LN229NC sh-3

Add lactic acid：10nMol

0.5h 1 6h 12h 24h

Mitochondrial oxidative phosphorylation after addition of lactate：

LN229

L-Lac - + - +

MFN1

ATP5A

DR1

SDHB

U251

ATP5A

DR1

SDHB

Actin：

lactylation：

H4lys16： U251 U251NC sh-3 LN229 LN229NC sh-3

L-Lac. U251 U251NC sh-3 LN229 LN229NC sh-3

H4. U251 U251NC sh-3 LN229 LN229NC sh-3

LDHB U251 U251NC sh-3 LN229 LN229NC sh-3

LDHA U251 U251NC sh-3 LN229 LN229NC sh-3

Actin： U251 U251NC sh-3 LN229 LN229NC sh-3

MBD6： U251NC U251-3 LN229NC LN229-3

BEST1： U251NC U251-3 LN229NC LN229-3

GRAD4： U251NC U251-3 LN229NC LN229-3

IL-6： U251NC U251-3 LN229NC LN229-3

IL-8： U251NC U251-3 LN229NC LN229-3

ACTIN: U251NC U251-3 LN229NC LN229-3

Supplementary section：Add lactic acid

H4K16la: U251NC U251-3 LN229NC LN229-3

H4：U251NC U251-3 LN229NC LN229-3

ACTIN: U251NC U251-3 LN229NC LN229-3
