# Supplementary material for: Knockdown of SUCLG2 inhibits glioblastoma proliferation and promotes apoptosis through LMNA acetylation and the mediation of H4K16la lactylation
Source: Cell Death Discov. 2025 Nov 17;11:534. doi: 10.1038/s41420-025-02856-4 (PMC12623996; doi:10.1038/s41420-025-02856-4)
Supplement: Supplementary file 7 — Specimen number S1 [file 41420_2025_2856_MOESM7_ESM.docx]

| Gender | Age | Pathological grading | IDH1 | IDH2 | TERT | | MGMT | |
| --- | --- | --- | --- | --- | --- | --- | --- | --- |
| Man | 43 | Normal |  |  | |  | |  |
| Man | 41 | Normal |  |  | |  | |  |
| Man | 35 | Normal |  |  | |  | |  |
| Woman | 50 | Normal |  |  | |  | |  |
| Woman | 11 | II | WT | WT | | WT | | negative |
| Woman | 49 | II | WT | WT | | MT | | negative |
| Woman | 49 | II | WT | WT | | WT | | positive |
| man | 38 | II | MT | WT | | WT | | negative |
| Woman | 29 | III | MT | WT | | WT | | positive |
| man | 53 | III | WT | WT | | MT | | positive |
| man | 54 | III | MT | WT | | WT | | positive |
| man | 48 | III | WT | WT | | WT | | positive |
| man | 36 | IV | WT | WT | | WT | | 19q heterozygous deletion |
| Woman | 32 | IV | MT | MT | | MT | | positive |
| man | 61 | IV | MT | WT | | WT | | positive |
| Woman | 40 | IV | MT | WT | | WT | | positive |

WT: wild type; MT: mutant type
